# AOP Report: Decreased ALDH1A (RALDH) activity leading to decreased fertility via disrupted meiotic initiation of fetal oogonia

**DOI:** 10.1016/j.crtox.2025.100257

**Published:** 2025-09-04

**Authors:** Monica K. Draskau, Cassy M. Spiller, Eleftheria M. Panagiotou, Johanna Zilliacus, Anna Beronius, Pauliina Damdimopoulou, Josephine Bowles, Terje Svingen

**Affiliations:** a National Food Institute, Technical University of Denmark, Kgs. Lyngby, Denmark; b School of Biomedical Sciences, University of Queensland, St. Lucia, Brisbane, Australia; c Department of Women’s and Children’s Health, Karolinska Institutet, Stockholm, Sweden; d Department of Gynecology and Reproductive Medicine, Karolinska University Hospital, Stockholm, Sweden; e Institute of Environmental Medicine, Karolinska Institutet, Stockholm, Sweden

**Keywords:** Meiosis, Retinoic acid, Toxicity mechanisms, Female fertility, Ovary, Oocytes

## Abstract

This report describes a novel adverse outcome pathway (AOP) highlighting how the inhibition of aldehyde dehydrogenase 1A (ALDH1A) enzymatic activity can lead to female infertility in mammals through disrupted meiotic entry of fetal germ cells (AOP-Wiki 398). In mammals, all-trans retinoic acid (atRA) can induce germ cell meiosis; during fetal life in females, germ cells enter meiosis prophase I. Reduced levels or absence of atRA disrupts this process, impairing germ cell development and leading to a reduced ovarian reserve in postnatal ovaries. The synthesis of atRA from vitamin A precursors involves an intermediate catalytic conversion of retinal by ALDH1A. Evidence for this AOP, particularly the upstream events, is primarily derived from mouse studies (both genetic models and exposure studies, including explanted ovaries). Human evidence, especially for downstream events, corroborates that the ovarian reserve directly impacts fertility. In reproductive toxicity studies (both animal studies and human epidemiology), fertility is a critical endpoint for chemical safety assessments. Although infertility has multiple causes, this AOP specifically captures events of perturbed meiosis due to reduced atRA signaling during development, thus supporting the use of *in silico* and *in vitro* data on nuclear receptor activity of the retinoic acid and retinoid X receptors (RAR/RXR) and atRA synthesis/expression to predict potential *in vivo* effects.

## Highlights

• Description of a novel AOP relevant for disrupted retinoid signaling.
• ALDH1A inhibition is linked to decreased fertility via disrupted germ cell meiosis.
• The AOP supports the use of *in silico* and *in vitro* data to predict *in vivo* effects.

## Graphical abstract

**Figure:**
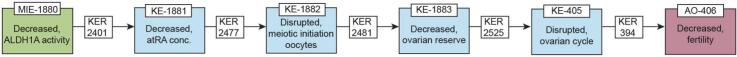

## Introduction and Background

Retinoic acid (RA) is an essential morphogen involved in various developmental processes, ranging from cell differentiation to body axis specification and organogenesis (Kam et al., 2012, Ghyselinck and Duester, 2019). The primary active form of RA, all-trans RA (atRA), is synthesized from dietary vitamin A precursors: retinol, retinyl esters and carotenoids (Paik et al., 2001, Harrison, 2012, Shannon et al., 2017, Cunningham and Duester, 2015). In target tissues, atRA acts as a ligand for the retinoic acid and retinoid X receptors (RAR and RXR), which influence gene regulation (Shannon et al., 2017). Although atRA is involved in numerous biological processes, the following discussion focusses on its role in gonad development and germ cell differentiation in mammals, particularly in the ovary, and the consequences on fertility that manifest in adulthood (Damdimopoulou et al., 2019).

In mammals, the differentiation of the two sexes relies on a genetic switch during early gonad differentiation. Initially, the gonads of XY and XX fetuses are morphologically indistinguishable and diverge into testes or ovaries based on genetic cues. XY gonads differentiate into testes in response to the Y-linked gene *Sry/SRY (Sex-determining region on the Y)*, while XX gonads differentiate into ovaries through a separate genetic pathway activated in the absence of *Sry* (Svingen and Koopman, 2013, Nicol and Yao, 2014, Wilhelm et al., 2025). Following gonadal sex determination, both testes or ovaries develop into multicellular and compartmentalized structures required for their dual function throughout life: sperm or oocyte production and sex hormone synthesis.

Similarly to the undifferentiated gonads, primordial germ cells are initially 'bipotential' and follow either a male or female fate depending on cues from the surrounding somatic environment. In fetal testes, germ cells enter a quiescent state until puberty, while in developing ovaries, they enter meiosis prophase I. In mice, retinoid signaling, specifically atRA, acts as a meiosis-inducing factor, playing a key role in sexual dimorphic germ cell programming (Bowles et al., 1979, Koubova et al., 2006). Thus, fetal ovaries require atRA for meiosis initiation, whereas fetal testes need the absence of atRA to avoid meiosis and enter cell cycle arrest (Spiller and Bowles, 2019).

The site of atRA production during gonadal development varies between species. In fetal mice, the mesonephros adjacent to the gonads is likely the primary site for atRA synthesis, expressing the enzymes ALDH1A2 (also known as RALDH2) and ALDH1A3 (RALDH3) (Bowles et al., 2018, Niederreither et al., 1999). The ovary also expresses ALDH1A1 (RALDH1), indicating potential for local atRA synthesis (Bowles et al., 2016, Mu et al., 2013). In humans, all three ALDH enzymes (ALDH1A, ALDH1B, and ALDH1C) are expressed in both testes and ovaries, suggesting *de novo* atRA synthesis capacity (Childs et al., 2011, Jørgensen and Rajpert-De, 2014, Le Bouffant et al., 2010). In human fetal ovaries, *ALDH1A1* expression peaks at meiotic onset, underscoring the time-dependent role of atRA in inducing meiosis (Le Bouffant et al., 2010). The requirement for atRA to induce the pre-meiotic factor STRA8 (Stimulated by retinoic acid 8) is conserved between rodents and humans, with atRA being actively produced to induce meiosis in human ovaries (Spiller and Bowles, 2019).

Various mouse studies suggest a broader role for retinoid signaling in fetal gonads. For instance, deletion of the atRA degrading enzyme *Cyp26b1* induces ectopic atRA in the fetal testis, disrupting steroidogenesis and partially feminizing male reproductive tracts (Bowles et al., 2018). Regulatory pathways involving factors such as *Stra8*; *Aldh1a*, and *RAR* potentially overlap with other somatic regulators and pathways, including *Ppar*, *Vdr*, *Tr*, *AhR*, *Fgf9*, and *Dax1* (Bowles et al., 2018, Bowles et al., 2010, Chambon, 1996, Murphy et al., 2007). This complex crosstalk between regulatory pathways highlights the intricate role of atRA in gonad development, impacting specific cells directly or indirectly and potentially affecting both somatic and germ cell niches with varied outcomes.

The OECD highlighted the retinoid signaling pathway in 2012 as a potential target for endocrine-disrupting chemicals (EDCs) (OECD, 2012). This pathway was one of seven identified as susceptible to environmental endocrine disruption, with measurable endpoints in new or existing OECD Test Guidelines. The European Commission and OECD supported the development of a Detailed Review Paper (DRP) on the retinoid system, which was adopted by the National Coordinators of the OECD Test Guideline Programme in April 2021 (OECD, 2021). Despite these efforts, there are still no endorsed adverse outcome pathways (AOPs) for RA signal disruption in the AOP-Wiki. The DRP on *‘the State of the Science on Novel In vitro and In Vivo Screening and Testing Methods and Endpoints for Evaluating Endocrine Disruptors’* identified the RA signaling pathway as a key area for potential endocrine disruption, with relevant endpoints measurable under OECD Test Guidelines. Continued support from the European Commission and OECD underscores the importance of understanding RA signaling in the context of endocrine disruption (OECD, 2021). Notably, a number of AOPs relevant for retinoid signaling have been proposed and published in the open literature, with focus on skeletal (Pierro et al., 2022), teratogenic (Menegola et al., 2021, Metruccio et al., 2020, Phan et al., 2024, Tonk et al., 2015), ocular (Cho et al., 2021), neuronal (Chen et al., 2020), and cardiovascular (Knudsen and Kleinstreuer, 2011) effects, with related AOPs currently under development in AOP-Wiki: cardiovascular (AOP-436), ocular (AOP-297), neuronal (AOP-532, −520, −523, −533), teratogenic (AOP-434), and reproductive (AOP-398, −400).

With this report, we provide a high-level overview of AOP-398 linking inhibition of ALDH1A enzymatic activity with female infertility (Text Box 1). A summary of the scientific evidence is included herein, whereas detailed information about empirical evidence, biological plausibility, uncertainties, and overall assessment of the AOP are provided under individual KE, KER and AOP pages on AOP-Wiki (aopwiki.org). A time-stamped snapshot of all included elements is also provided as Supplementary Data 1.

Text Box 1 **Adverse Outcome Pathway (AOP) ID.•Formal AOP title: Decreased ALDH1A (RALDH) activity leading to decreased fertility via disrupted meiotic initiation of fetal oogonia•AOP authors: M.K. Draskau, C.M. Spiller, E.M. Panagiotou, J. Zilliacus, A. Beronius, P. Damdimopoulou, J. Bowles, and T. Svingen•AOP numbers: 398•OECD work plan project number: 1.97•List of key events (KEs):**

**Table:** 

**ID**	**Title**	**linked AOPs**

1880	Decreased ALDH1A (RALDH) enzyme activity	398, 436
1881	Decreased, all-trans retinoic acid (atRA) concentration	398, 436
1882	Disrupted, initiation of meiosis of oogonia in the ovary	398
1883	Decreased, size of the ovarian reserve	398, 563
405	Disrupted, ovarian cycle	7, 345, 398,
406	Decreased, fertility	7, 18, 51, 64, 345, 348, 349, 396, 398, 492

### AOP description

**Fig. 1:**
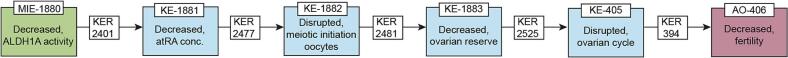
Graphical representation of AOP-398 linking reduced ALDH1A enzyme activity to decreased fertility (in females) via disrupted meiotic entry of oogonia during fetal life. MIE = molecular initiating event, KE = key event, AO = adverse outcome, KER = key event relationship.

The proposed AOP (Fig. 1) links decreased ALDH1A activity (MIE-1880) to decreased fertility in females (AO-406) through four intermediate key events (KEs): decreased atRA concentration (KE-1881), disrupted initiation of meiosis of oogonia (KE-1882), decreased size of the ovarian reserve (KE-1883), and disrupted ovarian cycle (KE-405). This causal pathway builds on the knowledge that oogonia must enter meiosis prophase I during fetal development, likely initiated by atRA's induction of *Stra8* expression (Childs et al., 2011, Baltus et al., 2006), to establish a healthy reserve of primordial follicles for adult fertility (Pepling and Spradling, 2001, Handel and Schimenti, 2010). Meiotic initiation is crucial for oocyte health and the establishment of the finite follicle pool, with oogonia failing to enter meiosis undergoing apoptosis, as evidenced in *Stra8*-null mice (Baltus et al., 2006). A reduced number of correctly formed primordial follicles during early development leads to faster depletion of the reserve during adulthood. When the reserve gets critically low, it cannot sustain normal folliculogenesis and steroidogenesis, resulting in irregular ovarian cycles and impaired fertility (O’Connor et al., 2001). This AOP notably connects fetal developmental disruptions (MIE) with adult female reproductive issues (AO), which in humans may separate the MIE from the AO by several decades.

Recognizing possible direct interference of biomolecules, ‘Decreased ALDH1A enzyme activity’ (MIE-1880) was designated as the MIE, leading to ‘decreased atRA concentration’ (KE-1881). Other events can also lead to decreased atRA levels locally in cells and tissues, which may be added later to this AOP to enhance the strength of initiating events and the capacity to incorporate test methods for screening potential adverse outcomes. Importantly, the MIE and first KE are also likely applicable to several AOs linked to disrupted retinoid signaling in other organs and organisms, as these steps convert dietary vitamin A (retinol, retinyl esters and carotenoids) into biologically active atRA, a molecule involved in numerous biological processes across animal species (Cunningham and Duester, 2015, Duester, 2008, Niederreither and Dollé, 2008), including the ovary at both fetal and adult life stages (Damdimopoulou et al., 2019).

While the MIE and first KE have wide applicability domains across vertebrates, this AOP becomes specific for mammals from the second KE, describing the consequence of atRA deficiency for fetal ovarian oocytes: ‘Disrupted initiation of meiosis of oogonia in the ovary’ (KE-1882). In this AOP, KE-1882 is linked directly to atRA signaling, as demonstrated largely in mouse studies; however, it can also occur by various other upstream events. Notably, other stressors can disrupt meiosis by other means, for example synaptic defects in mouse oocytes exposed *in utero* to bisphenol A (BPA) (Susiarjo et al., 2007). As such, KE-1882 may serve as a nodal point in future AOP networks. Because this KE is only meant to cover early meiotic entry during prophase; later events such as follicle formation could be described by additional KEs. The downstream events of KE-1882 pragmatically and minimally depict how disrupted meiotic entry affects the establishment of the follicle pool, crucial for subsequent female reproductive health from pre-puberty until menopause.

The fourth KE, ‘Decreased size of the ovarian reserve’ (KE-1883), signifies an ovary-specific outcome of disrupted entry into meiosis prophase I during fetal life, which commonly results in such oocytes being cleared by apoptosis. Importantly, the ovarian reserve declines in size with age through follicular atresia and follicular activation, and a smaller number of follicles at birth has been linked with earlier onset of menopause. Hence, any factors reducing meiotic entry and follicle assembly during early development, or increases follicle atresia or activation in later life, has the potential to shorten reproductive lifespan and increase the odds of infertility. Although the timing of follicle assembly differs between animal species (e.g. late gestation in humans versus early postnatal life in mice and rats), it is a relatively well conserved process, hence KE-1883 is applicable for different mammals. There are no good approaches to directly quantify the ovarian reserve, which is the sum of primordial follicles present in ovaries at any given time. Typically, the size of the reserve is estimated by counting follicles of different sizes (developmental stages), as described in OECD test guidelines (TGs) 416 and 443, assuming growing follicles reflect a normal reserve. However, it is a labor-intensive procedure to count follicles, and the numbers of growing follicles do not necessarily correlate with the number of primordial follicles. In humans, histological quantification of follicles in ovaries is typically not possible. Instead, serum biomarkers such as anti-Müllerian hormone (AMH) are used as a proxy (Broer et al., 2014). Since AMH is secreted by growing follicles, it does not directly inform about the number of primordial follicles i.e. the ovarian reserve.

KE-405 relates to ovarian cycle irregularities, which include variations in cycle length or ovulation problems such as deferred ovulation or anovulation. These irregularities indicate disturbances in any parts of the Hypothalamic-Pituitary-Ovarian (HPO) axis, which regulates reproductive processes. Therefore, KE-405 could also be considered an AO, as described in AOP-345 (Panagiotou et al., unpublished). Cycle irregularities can be measured *in vivo* by estrous cycle monitoring, an endpoint in several OECD guideline tests. Lastly, AO-406, ‘Decreased fertility’, refers to the capacity to conceive and is measured by calculating fertility rate based on the number of born offspring. This AO was chosen specifically for this AOP as its taxonomic domain is restricted to mammals with the focus on humans, mice, and rats. In humans, fecundity cannot be measured as is done in, for instance, fishes, frogs, and other model species (by counting the number of spawned eggs). Fertility, on the other hand, can be measured by the actual number of offspring produced, or in the case of no offspring, failure to achieve pregnancy after 12 months or more of regular unprotected sexual intercourse. Similar measurement would apply to rodent studies by counting the number of live pups, and in case of no offspring, provide evidence of a copulatory plug and a reasonable length of time with proven fertile male to demonstrate infertility via breeding assessment. It must also be recognized that human fertility rates are affected by a multitude of factors ranging from socioeconomic and cultural to personal decisions. Hence, offspring counts between rodents and humans are not directly comparable.

## Overview of AOP development approach

The development of AOP-398 followed guidelines from the OECD’s AOP Developer’s Handbook version 2.7 (obtained at https://aopwiki.org/handbooks/5) and applied a pragmatic approach as described in (Svingen et al., 2021). KER-2401 (Decreased, ALDH1A activity leads to Decreased, atRA concentration) and KER-2525 (Decreased, ovarian reserve leads to Disrupted, ovarian cycle) were considered canonical, with the remaining KERs developed using semi-systematic, transparent approaches for weight of evidence assessments. This approach is detailed in a published case-study for KER-2477 (Decreased, atRA concentration leads to Oocyte meiosis, disrupted) (Kam Draskau et al., 2022), and were also applied for the remaining KERs. KER-394 (ovarian cycle irregularities leads to impaired fertility) was pre-existing on AOP-Wiki and is currently part of two additional AOPs according to AOP-Wiki (AOP-7 and −345), it was recently updated in AOP-345 (Panagiotou et al., unpublished).

## Summary of scientific evidence

Weight of evidence assessment was conducted for the AOP overall to establish confidence in the causal relationships between linked KEs. Details can be found in the AOP-Wiki snapshot (provided as Supplementary Data 1 and available at https://aopwiki.org/aopwiki/snapshot/html_file/398-2025-07-04T11:52:11+00:00.html). Using modified Bradford-Hill criteria, we rated the overall confidence in AOP-398 as ‘moderate’, with KER-2525 identified as the weakest link due to limited mechanistic understanding and thus limited scientific evidence. This rating highlights the need for further empirical support to strengthen the mechanistic understanding and enhance the AOP's robustness for regulatory applications.

### Domain of applicability

The domain of applicability for an AOP is determined by the most narrowly restricted KE(R). In AOP-398, the MIE and first KE have a wide applicability domain that includes all ages and sexes across vertebrates, although the present KEs have been developed specifically for mammals. The remaining KEs, however, limits the applicability of AOP-398 to females during development, with evidence primarily from rodent studies, but also from humans, non-human primates, and livestock animals.

### Essentiality of key events

All KEs were separately assessed for their potential to impact the downstream sequence of KEs if they are modified, as summarized in Table 1. Essentiality analyses including assessment of uncertainties and inconsistencies are presented in Supplementary Data 2. Briefly, MIE-1880 and KE-1881 were considered ‘low’ overall even though strong evidence exists to suggest direct roles; the overall assessment of ‘low’ is based on there being evidence indicating that atRA synthesis is not the single determinant for initiation of meiosis in oocytes as discussed in Spiller & Bowles, 2022 and Shimada and Ishiguro, 2023 (Spiller and Bowles, 2022, Shimada and Ishiguro, 2023). KE-1882 of the sequence string was considered ‘high’, with no conflicting evidence, whereas KE-1883 and AO-405 were both considered ‘moderate’ as most evidence is indirect, as well as some conflicting evidence for KE-1883.

**Table 1:** Essentiality assessment of key events (KEs) of AOP-398. Retrieved literature and overall assessments are detailed in Supplementary Data 2. ****substantial evidence, ***good evidence, **moderate evidence, *some evidence.

Event	Direct evidence	Indirect evidence	Contradictory evidence	Overall essentiality assessment
KE-1880	****		***	Low
KE-1881	****		****	Low
KE-1882	****			High
KE-1883		***	*	Moderate
KE-405		**		Moderate

### Biological plausibility of the key event relationships

The biological plausibility of the five KERs was assessed individually by evaluating the mechanistic relationship between upstream and downstream KEs based on current established literature. The assessment is summarized in Table 2. All KERs were regarded to be supported by moderate to high weight of evidence. There are currently only adjacent KERs in AOP-398.

**Table 2:** Biological plausibility of key event relationships (KERs) of AOP-398.

ID	Assessment score	Rationale
KER-2401	High	It is well established that atRA is synthesized from retinaldehyde by the enzyme ALDH1A.
KER-2477	Moderate	A large body of evidence supports that atRA is involved in initiation of meiosis in ovarian germ cells but there also is data indicating that atRA is not the only determining factor.
KER-2481	High	It is well established that germ cells must enter meiosis prophase I during fetal development to establish the primordial follicles that ultimately make up the ovarian follicle pool.
KER-2525	Moderate	It is highly plausible that a reduced number of primordial follicles leads to irregularities in the ovarian cycle. The main challenge lies with quantification of effect and hence uncertainties regarding when the effect manifests relative to reduction in primordial follicles.
KER-394	High	It is well established that ovarian cyclicity is related to fertility.

### Empirical support for the key event relationships

More detailed descriptions and supporting references are found at respective KER pages in the time-stamped AOP snapshot (see Supplementary Data 1 or https://aopwiki.org/aopwiki/snapshot/html_file/398-2025-07-04T11:52:11+00:00.html). In short, and summarized in Table 3, the empirical support of KER-2401 (Reduced, ALDH1A enzyme activity leads to Decreased, atRA concentration) is considered moderate. Although it is known that atRA is metabolized from retinaldehyde by the three retinaldehyde dehydrogenases (ALDH1A1-3) there remain uncertainties surrounding RA biosynthesis and activity. For instance, although the spatiotemporal expression of ALDH enzymes is well characterized, it may not fully account for the tissue-specific activity of atRA, and the involvement of additional regulatory mechanisms cannot be entirely excluded (Rhinn and Dollé, 2012). Likewise, it is not fully understood how RA metabolic enzymes coordinate to establish the appropriate RA gradient (spatially, temporally, and in magnitude) during development (Duester, 2008).

**Table 3:** Empirical evidence for key event relationships (KERs) of AOP-398.

ID	Assessment score	Rationale
KER-2401	Moderate	Evidence exists but only for one stressor, WIN18,466.
KER-2477	Low	There are several studies with several different stressors that affect RA levels or RAR activity also affecting meiosis, but there also is conflicting data indicating that atRA is not the sole determining factor governing this process.
KER-2481	High	There are studies with several stressors showing same effect on meiosis and fertility.
KER-2525	High	There are studies with several stressors showing reduced number of follicles and affected ovarian cycling.
KER-394	Moderate	Although strong empirical evidence exists from numerous studies, they are chiefly based on the assessment of a single substance, DEHP.

Nevertheless, the bis-(dichloroacetyl)-diamine WIN18,446 has been shown in several studies to inhibit ALDH1A2 *in vitro* and *in vivo* resulting in inhibition of atRA synthesis. Empirical support for KER-2477 (Decreased, atRA concentration leads to Disrupted, initiation of meiosis of oogonia) is currently considered ‘low’. Although multiple studies demonstrate that changes in RA levels disrupt meiosis, some inconsistencies have been reported − highlighted under ‘Uncertainties, inconsistencies, and critical gaps’ − suggesting that atRA is not the sole determinant of this process (Bellutti et al., 2019, Chassot et al., 2020, Kumar et al., 2011, Vernet et al., 2020). Nonetheless, supporting evidence is substantial and includes meiotic failure in oocytes from vitamin A-deficient rats (Li and Clagett-Dame, 2009), as well as disrupted meiotic entry following treatment with the ALDH1A2 inhibitor WIN18,446 in mouse fetal ovary cultures (Rosario et al., 2020); citral (an RA synthesis inhibitor) in human fetal ovaries (Le Bouffant et al., 2010), and the RAR antagonists BMS-189453 and BMS-493 in both mouse and human fetal ovary cultures (Le Bouffant et al., 2010, Minkina et al., 2017) and mouse embryonic stem cells (Aoki and Takada, 2012). Additionally, numerous studies have shown that stimulating oocyte meiotic entry is possible. This has been demonstrated using the RARα agonist CD0336 in mouse fetal ovary cultures (Livera, 2000) or atRA in mouse embryonic stem cells (Aoki and Takada, 2012), mouse and camel oocyte cultures (Saadeldin et al., 2019, Tahaei et al., 2011), and human, mouse, or chicken fetal ovary cultures (Le Bouffant et al., 2010, Livera, 2000, Yu et al., 2013).

Evidence for both KER-2481 (Disrupted, initiation of meiosis of oogonia of the ovary leads to Reduced, ovarian reserve) and KER-2525 (Reduced, ovarian reserve leads to Disrupted, ovarian cycle) is considered ‘high’, since multiple *in vivo* studies in mice and rats have demonstrated that various chemical stressors can affect ovarian function. For instance, for KER-2481, mouse studies with di(2-ethylhexyl) phthalate (DEHP), BPA, paracetamol, or indomethacin all show an effect on meiosis and fertility (Dean et al., 2016, Mu et al., 2018, Zhang et al., 2012, Zhang et al., 2015), or follicle number (Zhang et al., 2009), whereas for KER-2525, mouse and rat studies with vinylcyclohexene diepoxide, 4-vinylcyclohexene, DEHP or BPA show reduced number of follicles and effects on ovarian cycling (Hannon et al., 2014, Hooser et al., 1994, Hu et al., 2018, Mayer et al., 2002, Mayer et al., 2004, Xu et al., 2010). Evidence for KER-394 (Disrupted, ovarian cycle leads to Decreased, fertility) is considered ‘moderate’, since it has been shown that various chemical substances can disrupt the estrous cycle, leading to persistent estrus, persistent diestrus, irregular cycles with extended durations, and ovulation problems. Common chemical classes causing cycle irregularities in rats, humans, and non-human primates include polychlorinated biphenyls (PCBs) and dioxins, which are linked to such irregularities in rats and humans (Chao et al., 2007, Li et al., 1995, Meerts, 2004). Various agricultural pesticides, including herbicides, fungicides, and fumigants, have also been associated with these effects (Bhattacharya and Keating, 2012, Bretveld et al., 2006). There are also numerous studies with phthalates, particularly DEHP, showing disrupted cyclicity and reduced fertility in rats, mice and sheep, although some studies are conflicting, as summarized under KER description on AOP-Wiki.

### Uncertainties, inconsistencies, and critical gaps

Although several studies have shown that atRA initiates meiosis in germ cells in mice (Koubova et al., 2006, Bowles et al., 2016, Teletin et al., 2017), other studies suggest that, despite being important, atRA is not essential for meiotic entry. This is evidenced by, for instance, meiosis occurring in *Aldh1a1*, *Aldh1a2*, and *Aldh1a3* knockout mice. Notably, although these *Aldha* isoforms show redundancy, compensating for the deletion of one or two (Bowles et al., 2016), double (*Aldh1a2/3*) and triple (*Aldh1a1/2/3*) knockout mice display reduced *Stra8* expression in oocytes, but meiosis still occurs, again suggesting a redundant role for atRA (Chassot et al., 2020, Kumar et al., 2011). Similarly, *RAR-α, −β, −γ* deletions lead to reduced *Stra8* expression but eventual meiosis (Vernet et al., 2020). While these knockouts can produce offspring, it is unclear if they have impaired fertility or altered oocyte pools.

Another uncertainty is that metabolic enzymes, such as ALDH class 1, can be used for both detoxification and for maintaining retinoid homeostasis. As a result, chemicals may indirectly affect retinoid homeostasis by competing for enzymes involved in RA production (Alnouti and Klaassen, 2008).

### Quantitative understanding

AOP-398 remains largely qualitative due to limited quantitative understanding of the KERs. The dose–response relationship between atRA concentrations in the ovary, meiotic initiation of oogonia, and the size of the resulting ovarian reserve is not well established. Furthermore, the connection between the number of follicles lost naturally during development and in adulthood, or due to other external factors, and overall fertility and reproductive health is not well understood. Specifically, it is currently unclear at what ovarian reserve size symptoms such as irregular cycles or infertility will start manifesting, rendering the overall AOP difficult to quantify.

### Relevance of the AOP

Numerous chemicals and drugs can disrupt the retinoic signaling pathways, highlighting the significance of this AOP for chemical toxicity, including endocrine disruption. Although the causal link between the MIE and AO in this AOP is considered moderate-to-high based on evidence, the biological plausibility is strong. Studies have shown that chemicals such as paracetamol can delay meiosis through disrupted *Stra8* expression, leading to reduced ovarian reserve size and fertility (Dean et al., 2016). Similarly, BPA and DEHP delay meiotic entry in female mice, although it remains unclear if these effects are due to disrupted retinoid signaling or indirect effects on *Stra8* regulation, potentially through epigenetic factors (Zhang et al., 2012, Zhang et al., 2015). In contrast, some studies report no effects of BPA on *Stra8* expression in ovaries (Lawson et al., 2011). Additional evidence is needed, particularly for quantitative relationships connecting specific KEs with adjacent or non-adjacent KEs, or the AO.

### Domain of applicability

The empirical evidence for this AOP is primarily derived from studies on humans, mice, and rats, thus restricting the applicability domain to these species. Given the AOP's focus on oocytes and its linkage to female fertility, the domain is limited to the female sex. The AOP spans from early fetal development to adulthood, with the MIE restricted to fetal stages and the AO to adult stages, which may be several decades in humans. Notably, retinoid signaling is highly conserved across animal taxa and is essential for vertebrate development (Ghyselinck and Duester, 2019, Bushue and Wan, 2010). Therefore, the early events of the AOP, particularly the MIE and KE1, have broader applicability.

## Potential applications

Evidence shows that environmental chemicals such as organochlorine pesticides, alkylphenols, and azole fungicides, can disrupt retinoid signaling and cause disease. However, current OECD test guidelines do not specifically address retinoid system modulation. OECD Expert Groups have emphasized the need for a greater focus on the modulation of the retinoid system by environmental chemicals and have highlighted the usability of the AOP framework in deciphering links between mechanisms and adverse outcomes (OECD, 2021). AOP-398 can provide a novel entry point to AOP-Wiki for RA signal disruption, with KEs encompassing currently available test methods that can also be integrated into test guidelines (Text Box 2). With RA signaling being crucial for development across animal taxa, and disruption linked to a wide range of dysmorphologies and diseases, future applications are potentially very broad, with upstream KEs being relevant for a plethora of toxicological pathways and disease outcomes, and downstream KEs more specific to female reproductive toxicity. It is also worth noting that retinoids also play a role in folliculogenesis and steroidogenesis in adulthood, suggesting that the female germline is susceptible to RA signaling disturbances throughout life (Damdimopoulou et al., 2019).

Text Box 2 **Examples of assays associated with individual key events (KEs) in AOP-398.**

**Table:** 

**ID**	**Title**	**Associated test assay**

1880	Decreased, ALDH1A (RALDH) enzyme activity	*in vitro:* Measure atRA or NADH formation. Several ALDH activity assay kits are commercially available (e.g. Aldefluor^TM^ kit).
1881	Decreased, all-trans retinoic acid (atRA) concentration	*in vitro:* Measure atRA, or indirect measure using reporter assays utilizing RAR-RXR-RARE or RXR-RXR-RARE promoter elements. *in vivo:* Measure directly atRA in serum (animals, humans) by chromatographic methods (HPLC, LC-MS).
1882	Disrupted, initiation of meiosis of oogonia in the ovary	*in vivo:* Assessing expression of meiotic markers in ovaries at mRNA or protein levels by primers/probes (e.g. qPCR, RNA *in situ*) or antibodies (e.g. IHC). Indirect measure using *Stra8*-GFP genetic mouse model.
1883	Decreased, size of the ovarian reserve	*in vivo*: Counting of ovarian follicles of different sizes in e.g. mouse/rat (included in OECD guideline tests no. 416, 443). Note, the ovarian reserve is composed of the primordial follicles that are typically not counted. Indirect measure by mRNA or protein expression of meiotic markers, or assessing ovary histology. In humans, assess AMH levels in blood, which reflects the number of small growing follicles and is assumed to correlate with ovarian reserve.
405	Disrupted, ovarian cycle	*in vivo:* Oestrus cyclicity monitoring (included for rats in OECD guideline tests no. 407, 416, 443).
406	Decreased, fertility	*in vivo:* Fertility rate calculation: number of offspring born (included for rats in OECD guideline tests no. 416, 443).

Female reproductive disorders, including decreased fertility, are on the rise and have been linked to exposure to environmental chemicals (Johansson et al., 2017, Parent et al., 2025). Establishing a biologically plausible causal pathway linking retinoid system modulation to reduced female fertility via disrupted oocyte meiotic entry in fetal ovaries thus provides a starting point for a more elaborate AOP network focused on retinoid signaling disruption. This pathway would highlight specific methods and assays available for assessing RA disruption and identify gaps in current methods where new efforts could be directed. Further to this, since MIEs (and other upstream KEs) can be detected rapidly and cost-effectively by alternative test assays – unlike apical outcomes that require lengthy and expensive testing – AOPs can be used to demonstrate the relevance and predictive power of early-stage assays for identifying potential reproductive toxicants. In this way, uncertainties can delineate the limits of inference from New Approach Methodologies (NAMs) to latent AO that may in many cases be temporally separated from each other by years, or even decades, in humans.

## Declaration of competing interest

The authors declare that they have no known competing financial interests or personal relationships that could have appeared to influence the work reported in this paper.

## Data Availability

Final PDF snapshots of all KE and KER descriptions are included as Supplementary Data 1 and can also be found in the AOP-Wiki (https://aopwiki.org/aopwiki/snapshot/html_file/398-2025-07-04T11:52:11+00:00.html).
