# Supplementary material for: AOP Report: Decreased ALDH1A (RALDH) activity leading to decreased fertility via disrupted meiotic initiation of fetal oogonia
Source: Curr Res Toxicol. 2025 Sep 4;9:100257. doi: 10.1016/j.crtox.2025.100257 (PMC12547181; doi:10.1016/j.crtox.2025.100257)
Supplement: Supplementary Data 1 [file mmc1.pdf]

AOP ID and Title:

AOP 398: Decreased ALDH1A (RALDH) activity leading to decreased fertility via disrupted meiotic initiation of fetal oogonia
Short Title: Decreased ALDH1A activity leading to decreased fertility

Graphical Representation

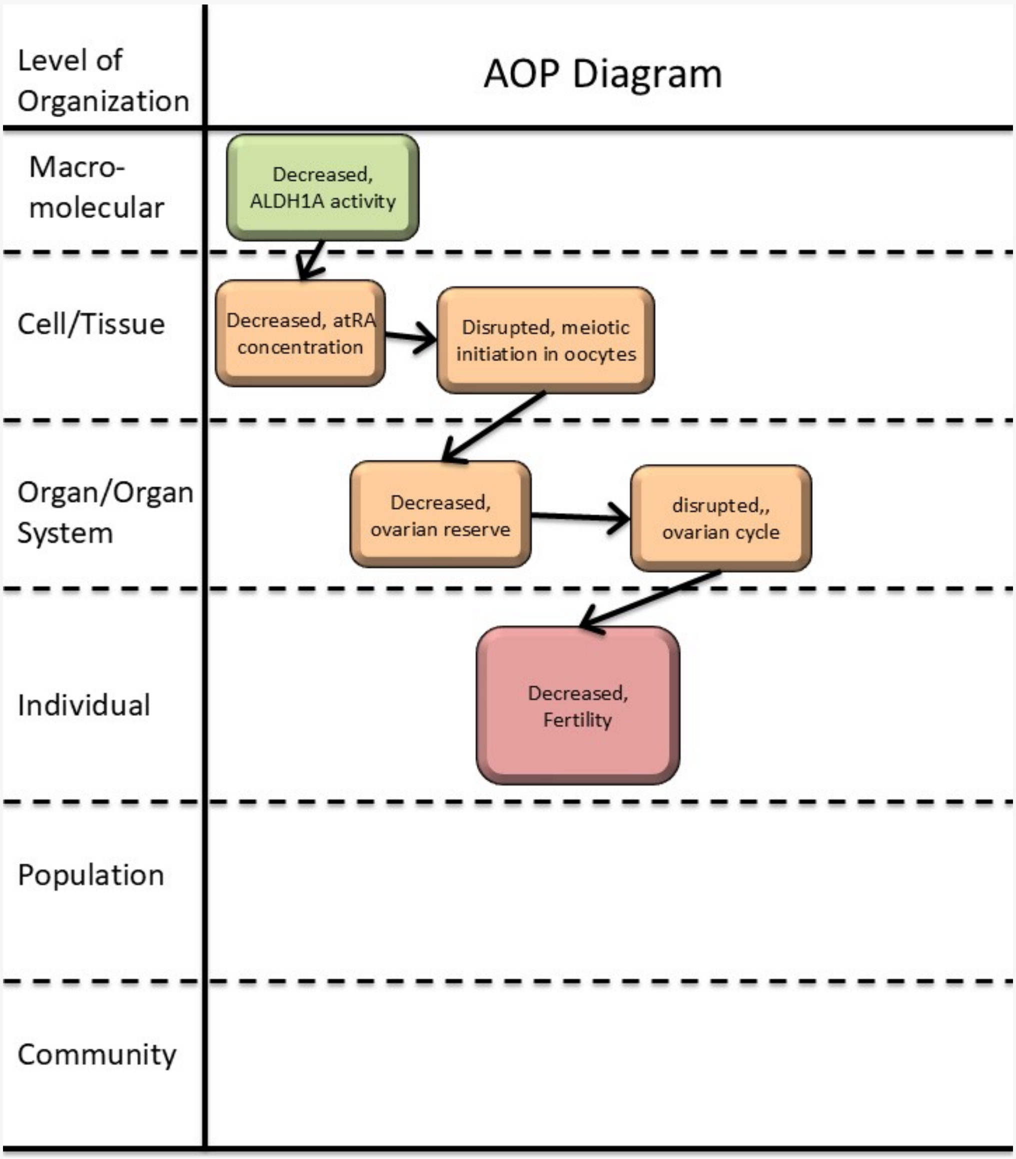

Authors

Monica Kam Draskau, Technical University of Denmark, Denmark

Cassy M. Spiller, University of Queensland, Australia

Josephine Bowles, University of Queensland, Australia

Eleftheria M. Panagiotou, Karolinska Institute, Sweden

Pauliina Damdimopoulou, Karolinska Institute, Sweden

Johanna Zilliacus, Karolinska Institute, Sweden

Anna Beronius, Karolinska Institute, Sweden

Terje Svingen, Terchnical University of Denmark, Denmark

Status

Author status	OECD status	OECD project	SAAOP status
Under development: Not open for comment. Do not cite	Under Development	1.97	Included in OECD Work Plan

Abstract

This AOP links inhibition of ALDH1A during fetal life with female infertility in adulthood. A key step in this AOP is a reduction in all-trans retinoic acid (atRA) locally in the fetal ovary, which prevents resident germ cells (oocytes) from entering meiosis. Evidence for this AOP, especially upstream events, draws heavily from mouse studies, both genetic models and from exposure studies (including explanted ovaries). Human evidence is also available, especially for downstream events where the oocyte pool/ovarian reserve is known to directly impact on fertility. In reproductive toxicity (animal studies and human epidemiology) fertility is an apical endpoint of high importance and has strong utility for chemical safety assessments. Infertility can be caused by many, and varied, factors, but this AOP focusses on linking perturbed meiosis through disrupted atRA signaling during development, thus supporting the use of data from in silico and in vitro measurements for interference with nuclear receptor activity (RAR/RXR) and atRA synthesis/expression to infer potential to cause in vivo effects.

Background

In mammals, the primordial germ cells are initially ‘bipotential’. They will develop into either oocytes or gonocytes in ovaries or testis, respectively, depending on cues from the somatic environment. Germ cells in the developing testis will enter a quiescent state and reactivate at the onset of puberty. In contrast, germ cells in the developing ovary will enter meiosis (prophase I) during fetal life. A key signaling event for this sexual dimorphic germ cell programming is retinoid signaling, with all-trans retinoic acid (atRA) acting as a meiosis-inducing factor (Spiller & Bowles, 2019).

The source of atRA during ovary development differs to some degree between species. In mice, the adjacent mesonephros, which expresses two enzymes necessary for the final step in atRA production, ALDH1A2 and ALDH1A3, is likely the main source of atRA at early developmental stages (Bowles et al, 2018; Bowles et al, 2006; Koubova et al, 2006; Niederreither et al, 1999). There is also the capacity for atRA to be produced within the ovary itself, due to local expression of the atRA-synthesizing enzyme ALDH1A1 (Bowles et al, 2016; Mu et al, 2013).

In humans, ALDH1A enzymes (ALDH1A, -1B and -1C) are expressed in both testes and ovaries of the developing fetus, which suggest a capacity for de novo synthesis of atRA (Childs et al, 2011; Jørgensen & Rajpert-De Meyts, 2014; le Bouffant et al, 2010), as is also the case in rabbits(Díaz-Hernández et al, 2019). One team studying human fetal ovaries reported a peak of ALDH1A1 expression at the onset of meiosis (le Bouffant et al, 2010), suggesting that meiotic onset in the human ovary depends on provision of atRA at the correct time. There seems to be conservation from rodent to human in terms of the requirement for atRA to induce the pre-meiotic factor STRA8. However, in mice atRA is produced by adjacent tissue and is present at high concentrations in the ovaries, whereas in human ovaries RA is present at only low levels and is then actively produced to induce meiosis in the ovary (Spiller & Bowles, 2019).

Summary of the AOP

Events

Molecular Initiating Events (MIE), Key Events (KE), Adverse Outcomes (AO)

Sequence	Type	Event ID	Title	Short name
1	MIE	1880	Decreased, ALDH1A (RALDH) enzyme activity	Decreased, ALDH1A activity
2	KE	1881	Decreased, all-trans retinoic acid (atRA) concentration	Decreased, atRA concentration
3	KE	1882	Disrupted, initiation of meiosis of oogonia in the ovary	Disrupted, meiotic initiation in oocyte
4	KE	1883	Decreased, size of the ovarian reserve	Decreased, ovarian reserve
5	KE	405	disrupted, ovarian cycle	disrupted, ovarian cycle

Sequence	Type	Event ID	Title	Short name
6	AO	406	decreased, Fertility	decreased, Fertility

Key Event Relationships

Upstream Event	Relationship Type	Downstream Event	Evidence	Quantitative Understanding
Decreased, ALDH1A (RALDH) enzyme activity	adjacent	Decreased, all-trans retinoic acid (atRA) concentration	High	Moderate
Decreased, all-trans retinoic acid (atRA) concentration	adjacent	Disrupted, initiation of meiosis of oogonia in the ovary	Moderate	Low
Disrupted, initiation of meiosis of oogonia in the ovary	adjacent	Decreased, size of the ovarian reserve	High	Moderate
Decreased, size of the ovarian reserve	adjacent	disrupted, ovarian cycle	Moderate	Low
disrupted, ovarian cycle	adjacent	decreased, Fertility	High	Low

Overall Assessment of the AOP

The majority of evidence supporting this AOP is derived from mouse studies, both in vitro (fetal ovary cultures) and in vivo (incl. genetic mouse models). There is also evidence from humans (in vitro ovary cultures), yet it is also recognized that there are some differences between mice and humans with regard to atRA synthesis, expression and potential role in meiotic initiation. Notably, an important link, yet not described as a separate key event, is the role for *Stra8* in meiotic initiation alongside the established role for atRA to control*Stra8* expression via RAR/RXR.

The evidence linking MIE with KE1 is considered as strong and regarded as canonical knowledge. Likewise, evidence for the downstream key events linking reduced oocyte pool/ovarian reserve with reduced fertility is very strong and regarded as canonical knowledge. The weak link in the overall AOP is the connection between reduced atRA levels and fertility via loss of oocytes during development. To strengthen this link, more evidence must be obtained; nevertheless, the remaining links are very strong and can be used to assess the impact of chemical stressors on female fertility. Yet, caution should be exercised with directly linking inhibition of ALDH1A2 with reduced fertility.

Domain of Applicability

Life Stage Applicability

Life Stage	Evidence
Development	High

Taxonomic Applicability

Term	Scientific Term	Evidence	Links
mouse	Mus musculus	High	NCBI
rat	Rattus norvegicus	Moderate	NCBI
human	Homo sapiens	Moderate	NCBI

Sex Applicability

Sex	Evidence
Female	High

- **Sex:** This AOP applies to females. atRA is also involved in meiosis of testicular gonocytes, but this occurs postnatally. In the female ovaries, atRA induces meiosis of oocytes during gestation, thus the spatiotemporal expression of atRA in the ovaries are tightly controlled. Finally, as this AOP is concerned with establishing the ovarian reserve/follicle pool through mechanisms that are unique to ovaries, restricting the AOP to female only is appropriate.
- **Life stages:** This AOP spans the period from mid- to late-gestation in mammals, all the way to adulthood where fertility is manifested. The upstream event pertains to fetal/neonatal life stages, whereas the downstream events pertain to adult reproductive life stages.
- **Taxonomy:** Strongest evidence for the role of atRA in regulating oocyte entry into meiosis stems from mouse studies, so the taxonomic applicability is strongest for this animal model. Studies have also been done in rats. Evidence for the same mechanisms in humans is less substantiated (Li & Clagett-Dame, 2009; Griswold et al,

2012; Spiller & Bowles, 2022; Jørgensen & Rajpert-De Meyts, 2014).

Essentiality of the Key Events

The essentiality of each key event (KE) was evaluated, meaning that if an upstream KE is blocked or does not occur, subsequent downstream KEs or the adverse outcome (AO) are prevented or altered. Both direct and indirect evidence of essentiality were assessed according to the OECD developer's handbook (see Appendix B, 1t3n0mxigk_App_B_Essentiality_2025.pdf) with a summary provided in Table 1.

Table 1: Essentiality assessment of KEs for AOP 398

Retrieved literature and overall assessments are detailed in Appendix B. ****substantial evidence, ***good evidence, **moderate evidence, *some evidence.

Event	Direct evidence	Indirect evidence	Contradictory evidence	Overall essentiality assessment
KE-1880	****		***	Low
KE-1881	****		****	Low
KE-1882	****			High
KE-1883		***	*	Moderate
KE-405		**		Moderate

Uncertainties, inconsistencies and data gaps

In mice, there is strong evidence to support the view that atRA is important for initiating meiosis in germ cells (Bowles et al, 2016; Spiller et al, 2017; Teletin et al, 2017). Some studies suggest that atAR is not critical but important for meiotic entry under normal physiological conditions by evidencing meiosis in *Aldh1a1*, *Aldh1a2* and *Aldh1a3* ablated mice, individually and in tandem (Bellutti et al, 2019; Chassot et al, 2020; Kumar et al, 2011); however, additional studies have shown redundant roles between all three Aldha isoforms which can compensate for deletion of one or two (Bowles et al, 2016). More specifically, both double (*Aldh1a2/3*) and triple (*Aldh1a1/2/3*) knockout mouse models display reduced *Stra8* expression in oocytes, yet oocytes eventually go through meiosis, which could suggest a redundant role for atRA for meiosis in the ovaries (Chassot et al, 2020; Kumar et al, 2011). A similar phenotype with reduced Stra8 expression but eventual meiotic initiation is seen for deletion of atRA receptors RAR-α, -β, -γ) in mice (Vernet et al, 2020). But, although RAR knockouts were also capable of producing offspring, it remains unclear if any of the above-mentioned mouse models display impaired fertility or whether the size of their oocyte pools are affected.

Weight of Evidence Summary

Biological Plausibility, coherence, and consistency of the experimental evidence

The role for ALDH1A2 in the synthesis of atRA is well established as an essential component of regulating regional expression of retinoid species during development. It is also well established that atRA is an inducer of meiosis in germ cells in mice; however, there is some debate about the essentiality of atRA in this process in human fetal ovaries. The requirement for oocytes to enter the first phase of meiosis during fetal development is also well established, hence the biological plausibility linking meiotic failure with loss of oocytes at later developmental stages is strong.

Although non-meiotic oocytes can survive in germ cell nests and during nest breakdown, they will ultimately be eliminated from the oocyte pool of competent follicles. There is therefore a direct link between meiotic entry and fertility during adulthood. Thus, this AOP provides a plausible chain of events linking reduced atRA during fetal life with reduced ovarian reserve and fertility during reproductive age. The strength of the downstream KEs and KER – reduced ovarian reserve and reduced fertility – is very well documented and thus the biological plausibility is very strong. Evidence for a direct link between the AO and perturbed atRA synthesis, or reduced atRA levels, during early development comes mainly from mouse studies; yet the relationship is regarded biologically plausible also in humans, but with weight of evidence not being as strong.

The overall evidence assessment scores for each KER is summarized in the below Table:

Table 2: Biological plausibility of key event relationships of AOP-398.

ID	Assessment score	Rationale
KER-2401	High	It is well established that atRA is synthesized from retinaldehyde by the enzyme ALDH1A.
KER-2477	Moderate	A large body of evidence supports that atRA is involved in initiation of meiosis in ovarian germ cells but there also is conflicting data indicating that atRA is not the only determining factor.

KER-2481	High	It is well established that germ cells must enter meiosis prophase I during fetal development to establish the primordial follicles that ultimately make up the ovarian follicle pool.
KER-2525	Moderate	It is highly plausible that a reduced number of primordial follicles leads to irregularities in the ovarian cycle. The main challenge lies with quantification of effect and hence uncertainties regarding when the effect manifests relative to reduction in primordial follicles.
KER-394	High	It is well established that ovarian cyclicity is related to fertility.

Empirical support

Concordance of dose-response relationships

The quantitative understanding of dose-response relationships in this AOP is limited. Whilst the relative levels of endogenous atRA produced by the ovary (for any species) remains unknown, similarly, the quantitative relationship between atRA levels and induction of meiosis also remains unclear. Nevertheless, it is has been conclusively shown that low levels of exogenous atRA can induce mouse and rat germ cells to enter meiosis both in vitro and ex vivo (Bowles et al, 2006; Livera et al, 2000). Likewise, atRA is necessary to achieve meiosis in in vitro-derived oocytes via PGCLCs (Miyauchi et al, 2017).

Temporal concordance among the key events and the adverse outcome

This AOP bridges two different life stages: fetal/perinatal and adult/reproductive age. The adverse outcome is the result of perturbation taking place during early stages of ovary development. In mice, rats and humans, the oocytes must enter meiosis prophase in order to establish the follicle pool/ovarian reserve postnatally. Thus, the AOP focusses on chemical perturbations during fetal life, which occurs around E13-E16 in mice and E15-E18 is rats, or first trimester in humans (Peters, 1970), but the adverse outcome does not manifest until adulthood.

There is strong temporal concordance between the various key events, from inhibition of ALDH1A2 (RALDH2) that leads to reduced atRA synthesis. In turn, atRA must be present in the fetal ovaries at the time when oocytes are supposed to enter meiosis mid-gestation in mice (or first trimester in human). With a significant reduction in available atRA the oocytes will not enter meiosis, ultimately leading to the downstream key event of loss of oocytes beyond what is normal. The number of oocytes, or the oocyte pool/ovarian reserve, in turn will affect ovary function and fertility at reproductive stages, hence the temporal sequence of events is rational based on the biological process.

Strength, consistency, and specificity of association of adverse effect and initiating event

In mice, there is strong evidence to support the view that atRA is an inducer of meiosis in germ cells, with consistent results from in vitro (PGCLCs), ex vivo (ovary cultures) and in vivo studies as listed under KE 2477. There is strong evidence showing the importance of RA for female fertility, but this relates to many aspects of reproductive development and function from fetal life to adulthood, including maintaining pregnancy (Clagett-Dame & Knutson, 2011). Thus, it can be difficult to distill exactly how atRA-controlled meiotic entry of oocytes directly link to reduced fertility. Nevertheless, a direct relationship is strongly supported by the fact that *Stra8*-depleted mice are infertile with small ovaries lacking oocytes (Baltus et al, 2006) and that *Stra8* induction in germ cells is controlled by atRA in mice, rats and humans (Bowles et al, 2006; Childs et al, 2011; Koubova et al, 2006; Livera et al, 2000). Furthermore, vitamin A-deficient (VAD) mice display delayed or failed meiotic entry of fetal oocytes depending on level of Vitamin A deficiency (Li & Clagett-Dame, 2009).

More detailed descriptions and supporting references are found at respective KER pages on AOPwiki. Overall assessments are summarized in **Table 3.**

Table 3: Empirical evidence for key event relationships of AOP-398.

ID	Assessment score	Rationale
KER-2401	Moderate	Evidence exists but only for one stressor, WIN18,466.
KER-2477	Low	There are several studies with several different stressors that affect RA levels or RAR activity also affecting meiosis, but there also is conflicting data indicating that atRA is not the sole determining factor governing this process.
KER-2481	High	There are studies with several stressors showing same effect on meiosis and fertility.

KER-2525	High	There are studies with several stressors showing reduced number of follicles and affected ovarian cycling.
KER-394	Moderate	Although strong empirical evidence exists from numerous studies, they are chiefly based on the assessment of a single substance, DEHP.

Quantitative Consideration

This AOP is still largely qualitative, as the quantitative understanding between chemical potency and perturbation of KEs are insufficient. This relates to the dose-response relationship between concentrations of atRA in the ovary relative to meiotic initiation of oocytes. It also relates to the relationship between number of lost oocytes during development relative to the oocyte pool/ovarian reserve, as there naturally is a large loss of oocytes during development.

Considerations for Potential Applications of the AOP (optional)

Currently disrupted retinoid signaling is not directly tested for in OECD TG studies; however, in, for example, the identification of endocrine disruptors, the R-modality is highlighted as a pathway that should be included. Hence, this AOP provides added support for inclusion of retinoid signaling-relevant assays to be included in testing or screening strategies.

This AOP can be used to identify chemicals that inhibit ALDH1A activity (e.g., through in vitro assays for retinoic acid biosynthesis) as potential reproductive toxicants, facilitating prioritization for further testing. It also provides a mechanistic basis for linking molecular-level perturbations to reproductive outcomes, supporting weight-of-evidence approaches in regulatory risk assessment and justifying restrictions on chemicals identified as disrupting this pathway.

References

Baltus AE, Menke DB, Hu YC, Goodheart ML, Carpenter AE, de Rooij DG, Page DC (2006) In germ cells of mouse embryonic ovaries, the decision to enter meiosis precedes premeiotic DNA replication. *Nat Genet* **38:** 1430-1434

Bellutti L, Abby E, Tourpin S, Messiaen S, Moison D, Trautmann E, Guerquin MJ, Rouiller-Fabre V, Habert R, Livera G (2019) Divergent Roles of CYP26B1 and Endogenous Retinoic Acid in Mouse Fetal Gonads. *Biomolecules* **9:** 536

Bowles J, Feng CW, Inseson J, Miles K, Spiller CM, Harley VR, Sinclair AH, Koopman P (2018) Retinoic Acid Antagonizes Testis Development in Mice. *Cell Rep* **24:** 1330-1341

Bowles J, Feng CW, Miles K, Inseson J, Spiller CM, Koopman P (2016) ALDH1A1 provides a source of meiosis-inducing retinoic acid in mouse fetal ovaries. *Nat Commun* **7:** 10845

Bowles J, Knight D, Smith C, Wilhelm D, Richman J, Mamiya S, Yashiro K, Chawengsaksophak K, Wilson MJ, Rossant J, Hamada H, Koopman P (2006) Retinoid signaling determines germ cell fate in mice. *Science* **312:** 596-600

Chassot AA, Le Rolle M, Jolivet G, Stevant I, Guigonis JM, Da Silva F, Nef S, Pailhoux E, Schedl A, Ghyselinck NB, Chaboissier MC (2020) Retinoic acid synthesis by ALDH1A proteins is dispensable for meiosis initiation in the mouse fetal ovary. *Sci Adv* **6:** eaaz1261

Chatzi C, Cunningham TJ, Duester G (2013) Investigation of retinoic acid function during embryonic brain development using retinaldehyde-rescued Rdh10 knockout mice. *Dev Dyn* **242:** 1056-1065

Childs AJ, Cowan G, Kinnell HL, Anderson RA, Saunders PTK (2011) Retinoic Acid signalling and the control of meiotic entry in the human fetal gonad. *PLoS One* **6:** e20249

Clagett-Dame M, Knutson D (2011) Vitamin A in Reproduction and Development. *Nutrients* **3:** 385-428

Díaz-Hernández V, Caldelas I, Merchant-Larios H (2019) Gene Expression in the Supporting Cells at the Onset of Meiosis in Rabbit Gonads. *Sex Dev* **13:** 125-136

Feng CW, Burnet G, Spiller CM, Cheung FKM, Chawengsaksophak K, Koopman P, Bowles J (2021) Identification of regulatory elements required for Stra8 expression in fetal ovarian germ cells of the mouse. *Development* **148:** dev194977

Griswold MD, Hogarth CA, Bowles J, Koopman P (2012) Initiating meiosis: the case for retinoic acid. Biol Reprod 86: 35

Grive KJ, Freiman RN (2015) The developmental origins of the mammalian ovarian reserve. *Development* **142:** 2554-2563

Jørgensen A, Rajpert-De Meyts E (2014) Regulation of meiotic entry and gonadal sex differentiation in the human: normal and disrupted signaling. *Biomol Concepts* **5:** 331-341

Kalampokas T, Shetty A, Maheswari A (2014) Vitamin A Deficiency and Female Fertility Problems: A Case Report and Mini Review of the Literature. *J Women's Health Care* **3:** 6

Koubova J, Menke DB, Zhou Q, Capel B, Griswold MD, Page DC (2006) Retinoic acid regulates sex-specific timing of meiotic initiation in mice. *Proc Natl Acad Sci U S A* **103:** 2474-2479

Krężel W, Rühl R, de Lera AR (2019) Alternative retinoid X receptor (RXR) ligands. *Mol Cell Endocrinol* **491:** 110436

Kumar S, Chatzi C, Brade T, Cunningham TJ, Zhao X, Duester G (2011) Sex-specific timing of meiotic initiation is regulated by Cyp26b1 independent of retinoic acid signalling. *Nat Commun* **2:** 151

le Bouffant R, Guerquin MJ, Duquenne C, Frydman N, Coffigny H, Rouiller-Fabre V, Frydman R, Habert R, Livera G (2010) Meiosis initiation in the human ovary requires intrinsic retinoic acid synthesis. *Hum Reprod* **25:** 2579-2590

Li H, Clagett-Dame M (2009) Vitamin A deficiency blocks the initiation of meiosis of germ cells in the developing rat ovary in vivo *Biol Reprod* **81:** 996-1001

Livera G, Rouiller-Fabre V, Valla J, Habert R (2000) Effects of retinoids on the meiosis in the fetal rat ovary in culture. *Mol Cell Endocrinol* **165:** 225-231

Miyauchi H, Ohta H, Nagaoka S, Nakaki F, Sasaki K, Hayashi K, Yabuta Y, Nakamura T, Yamamoto T, Saitou M (2017) Bone morphogenetic protein and retinoic acid synergistically specify female germ-cell fate in mice. *EMBO J* **36:** 3100-3119

Mu X, Wen J, Guo M, Wang J, Li G, Wang Z, Teng Z, Cui Y, Xia G (2013) Retinoic acid derived from the fetal ovary initiates meiosis in mouse germ cells. *J Cell Physiol* **228:** 627-639

Niederreither K, Subbarayan V, Dollé P, Chambon P (1999) Embryonic retinoic acid synthesis is essential for early mouse post-implantation development. *Nat Genet* **21:** 444-448

Nilsson, C and Working Group (2020) Retinoids in Mammalian Reproduction, with an Initial Scoping Effort to Identify Regulatory Methods. Available at https://norden.diva-portal.org/smash/record.jsf?pid=diva2%3A1424722&dswid=-4522

Peters H (1970) Migration of gonocytes into the mammalian gonad and their differentiation. *Philos Trans R Soc Lond B Biol Sci* **259:** 91-101

Shannon SR, Moise AR, Trainor PA (2017) New insights and changing paradigms in the regulation of vitamin A metabolism in development. *Wiley Interdiscip Rev Dev Biol* **6:** 10.1002/wdev.1264

Spiller C, Bowles J (2019) Sexually dimorphic germ cell identity in mammals. *Curr Top Dev Biol* **134:** 252-288

Spiller C, Koopman P, Bowles J (2017) Sex Determination in the Mammalian Germline. *Annu Rev Genet* **51:** 265-285

Teletin M, Vernet N, Ghyselinck NB, Mark M (2017) Roles of Retinoic Acid in Germ Cell Differentiation. *Curr Top Dev Biol* **125:** 191-225

Vernet N, Condrea D, Mayere C, Féret B, Klopfenstein M, Magnant W, Alunni V, Teletin M, Souali-Crespo S, Nef S, Mark M, Ghyselinck NB (2020) Meiosis occurs normally in the fetal ovary of mice lacking all retinoic acid receptors. *Sci Adv* **6:** eaaz1139

Appendix 1

List of MIEs in this AOP

Event: 1880: Decreased, ALDH1A (RALDH) enzyme activity

Short Name: Decreased, ALDH1A activity

Key Event Component

Process	Object	Action
retinoic acid biosynthetic process	retinal dehydrogenase 1	decreased

AOPs Including This Key Event

AOP ID and Name	Event Type
Aop:398 - Decreased ALDH1A (RALDH) activity leading to decreased fertility via disrupted meiotic initiation of fetal oogonia	MolecularInitiatingEvent
Aop:436 - Inhibition of RALDH2 causes reduced all-trans retinoic acid levels, leading to transposition of the great arteries	MolecularInitiatingEvent

Stressors

Name
Benomyl
WIN18,466
(~13~C,~15~N_2_)Cyanamide
Daidzein
Molinate
Pebulate
Vernolate
Butylate
Tri-allate
Cycloate

Biological Context

Level of Biological Organization
Molecular

Cell term

Cell term
eukaryotic cell

Domain of Applicability

Taxonomic Applicability

Term	Scientific Term	Evidence	Links
human	Homo sapiens	Moderate	NCBI
mouse	Mus musculus	High	NCBI
rat	Rattus norvegicus	Moderate	NCBI

Life Stage Applicability

Life Stage	Evidence
All life stages	High

Sex Applicability

Sex	Evidence
Male	High
Female	High

The retinoid signaling system is highly conserved across distant animal species(Bushue & Wan, 2010; Rhinn & Dollé, 2012).

Key Event Description

The oxidation of retinal to all-trans retinoic acid (atRA) is an irreversible reaction carried out by retinaldehyde dehydrogenases ALDH1A1, ALDH1A2, ALDH1A3 (RALDH1, RALDH2, RALDH3). ALDH1A2 is responsible for the second step of the metabolism of vitamin A into atRA (Chatzi et al, 2013; Shannon et al, 2017).The role of that reaction is to maintain atRA concentrations, with ALDH1A2 being most active during early development (Koppaka et al, 2012; Shannon et al, 2017). *Raldh2*-deficient mice exhibit severe developmental defects due to loss of atRA, but the phenotype is rescued by administration of exogenous RA (Niederreither et al, 1999). Thus, ALDH1A2 activity is essential for atRA-dependent developmental processes.

How it is Measured or Detected

There are no OECD validated assays for measuring ALDH1A2 inhibition.

ALDH1A2 mRNA and protein levels can be measured using various probes, antibodies as well as ELISA kits that are commercially available.

Enzyme activity can be assessed in assays including measurement of atRA formation(Arnold et al, 2015) or NADH formation (Harper et al, 2018; Schindler et al, 1998) and several ALDH activity assay kits using different approaches are commercially available; e.g. Aldeflour^{TM} kit (Flahaut et al, 2016).

References

Allen EMG, Anderson DGR, Florang VR, Khanna M, Hurley TD, Doorn JA (2010) Relative inhibitory potency of molinate and metabolites with aldehyde dehydrogenase 2: implications for the mechanism of enzyme inhibition. *Chem Res Toxicol* **23:** 1843-1850

Arnold SL, Kent T, Hogarth CA, Schlatt S, Prasad B, Haenisch M, T. W, Muller CH, Griswold MD, Amory JK, Isoherranen N (2015) Importance of ALDH1A enzymes in determining human testicular retinoic acid concentrations. *J Lipid Res* **56:** 342-357

Bushue N, Wan YJY (2010) Retinoid pathway and cancer therapeutics. *Adv Drug Deliv Rev* **62:** 1285-1298

Chatzi C, Cunningham TJ, Duester G (2013) Investigation of retinoic acid function during embryonic brain development using retinaldehyde-rescued Rdh10 knockout mice. *Dev Dyn* **242:** 1056-1065

Chen Y, Zhu JY, Hong KH, Mikles DC, Georg GI, Goldstein AS, Amory JK, Schönbrunn E (2018) Structural Basis of ALDH1A2 Inhibition by Irreversible and Reversible Small Molecule Inhibitors. *ACS Chem Biol* **13:** 582-590

Flahaut M, Jauquier N, Nardou K, Bourloud KB, Joseph JM, Barras D, Widmann C, Gross N, Renella R, Mühlethaler-Mottet A (2016) Aldehyde dehydrogenase activity plays a Key role in the aggressive phenotype of neuroblastoma. *BMC Cancer* **16:** 781

Harper AR, Le AT, Mather T, Burgett A, Berry W, Summers JA (2018) Design, synthesis, and ex vivo evaluation of a selective inhibitor for retinaldehyde dehydrogenase enzymes. *Bioorg Med Chem* **26:** 5766-5779

Koppaka V, Thompson DC, Chen Y, Ellermann M, Nicolaou KC, Juvonen RO, Petersen D, Deitrich RA, Hurley TD, Vasilio V (2012) Aldehyde dehydrogenase inhibitors: a comprehensive review of the pharmacology, mechanism of action, substrate specificity, and clinical application. *Pharmacol Rev* **64:** 520-539

Lowe ED, Gao GY, Johnson LN, Keung WM (2008) Structure of daidzin, a naturally occurring anti-alcohol-addiction agent, in complex with human mitochondrial aldehyde dehydrogenase. *J Med Chem* **51:** 4482-4487

Nagasawa HT, DeMaster EG, Redfern B, Shirota FN, Goon DJ (1990) Evidence for nitroxyl in the catalase-mediated bioactivation of the alcohol deterrent agent cyanamide. *J Med Chem* **33:** 3120-3122

Niederreither K, Subbarayan V, Dollé P, Chambon P (1999) Embryonic retinoic acid synthesis is essential for early mouse post-implantation development. *Nat Genet* **21:** 444-448

Paik J, Haenisch M, Muller CH, Goldstein AS, Arnold S, Isoherranen N, Brabb T, Treuting PM, Amory JK (2014) Inhibition of retinoic acid biosynthesis by the bisdichloroacetyldiamine WIN 18,446 markedly suppresses spermatogenesis and alters retinoid metabolism in mice. *J Biol Chem* **289:** 15104-15117

Quistad GB, Sparks SE, Casida JE (1994) Aldehyde dehydrogenase of mice inhibited by thiocarbamate herbicides. *Life Sci* **55:** 1537-1544

Rhinn M, Dollé P (2012) Retinoic acid signalling during development. *Development* **139:** 843-858

Schindler JF, Berst KB, Plapp BV (1998) Inhibition of human alcohol dehydrogenases by formamides. *J Med Chem* **41:** 1696-1701

Shannon SR, Moise AR, Trainor PA (2017) New insights and changing paradigms in the regulation of vitamin A metabolism in development. *Wiley Interdiscip Rev Dev Biol* **6:** 10.1002/wdev.1264

Shirota FN, DeMaster EG, Nagasawa HT (1987) Cyanide is a product of the catalase-mediated oxidation of the alcohol deterrent agent, cyanamide. *Toxicol Lett* **37:** 7-12

Staub RE, Quistad GB, Casida JE (1998) Mechanism for benomyl action as a mitochondrial aldehyde dehydrogenase inhibitor in mice. *Chem Res Toxicol* **11:** 535-543

List of Key Events in the AOP

Event: 1881: Decreased, all-trans retinoic acid (atRA) concentration

Short Name: Decreased, atRA concentration

Key Event Component

Process	Object	Action
retinoic acid biosynthetic process	all-trans-retinoic acid	decreased

AOPs Including This Key Event

AOP ID and Name	Event Type
Aop:398 - Decreased ALDH1A (RALDH) activity leading to decreased fertility via disrupted meiotic initiation of fetal oogonia	KeyEvent
Aop:436 - Inhibition of RALDH2 causes reduced all-trans retinoic acid levels, leading to transposition of the great arteries	KeyEvent

Stressors

Name
WIN18,466
Ethanol
Diethylaminobenzaldehyde

Biological Context

Level of Biological Organization
Tissue

Domain of Applicability

Taxonomic Applicability

Term	Scientific Term	Evidence	Links
mouse	Mus musculus	High	NCBI
rat	Rattus norvegicus	High	NCBI
human	Homo sapiens	High	NCBI
Vertebrates	Vertebrates	Moderate	NCBI

Life Stage Applicability

Life Stage	Evidence
All life stages	Moderate

Sex Applicability

Sex	Evidence
Male	High
Female	High

The retinoid signaling system is highly conserved across animal species(Bushue & Wan, 2010b; Rhinn & Dollé, 2012). atRA acts as a ligand for the nuclear retinoic acid (RAR) receptors, which upon activation regulate gene transcription in target cells. The type and number of RARs differ between evolutionary distant animals, but functionally they are all involved in the regulation of development.

Key Event Description

All-trans retinoic acid (atRA) is the active form of vitamin A/all-trans retinol and is involved in regulating a large number of developmental processes (Bushue & Wan, 2010a; Ghyselinck & Duester, 2019). Although 9-cis RA and 13-cis RA are other metabolic derivatives of vitamin A, atRA is generally considered the primary active metabolite during development, mainly acting as a short-range paracrine signaling molecule (Cunningham & Duester, 2015). atRA exerts dose-dependent effects on morphogenesis, so disruption to atRA concentrations during development can lead to malformations in numerous tissues and organs. During development the spatiotemporal regulation of atRA concentrations in target tissues is tightly controlled by a balance of synthesis and degradation enzymes (Kedishvili,

2013).

Cellular atRA synthesis starts by oxidation of vitamin A to retinaldehyde (RAL) by retinol dehydrogenase-10 (RDH10). RAL is then irreversibly converted to atRA by RAL dehydrogenases (ALDH1A1, ALD1A2, or ALDH1A3). To maintain appropriate retinoid levels in tissues, RAL can be converted back to retinol by enzymatic reactions; further retinoid levels can be controlled by enzymatic degradation of atRA by the cytochrome P450 enzymes CYP26A1, CYP26B1, or CYP26C1, which are differentially expressed throughout the mammalian body (Isoherranen & Zhong, 2019; Shimozono et al, 2013). Inhibition/disruption of any of the enzymes of the atRA synthesis pathway, or increased expression of the atRA degradation enzymes can lead to decreased concentrations of atRA in target cells(Kedishvili, 2013).

The atRA functions as a ligand for the nuclear retinoic acid receptors (RARs), which form heterodimers with the retinoid X receptors (RXRs); the atRA:RAR:RXR complex then binds to retinoic acid response elements (RAREs) upstream of target genes, leading to activation or repression of gene expression in target cells (Chambon, 1996; le Maire et al, 2019). The type and number of RAR/RXRs differ between evolutionary distant animals, but functionally they are all involved in the regulation of development (Gutierrez-Mazariegos et al, 2014).

How it is Measured or Detected

Direct measurements of atRA in serum (humans, animals) can be performed by various chromatographic methods (Gundersen, 2006), including high performance liquid chromatography (HPLC) or liquid chromatography-tandem mass spectrometry (LC-MS) (Morgenstern et al, 2021).

Indirect measurements in cells and animal models can be performed with reporter assays utilizing RAR-RXR-RARE or RXR-RXR-RARE promoter elements, which are activated by atRA, driving expression of reporter proteins. These reporter assays can detect the presence of atRA in tissues in a semi-quantitative manner. Examples include reporter mouse lines (Carlsen et al, 2021; Rossant et al, 1991; Solomin et al, 1998), reporter cell lines (Wagner et al, 1992) and transient transfection of constructs for in vitro cell-based assays (Chassot et al, 2020).

References

Arnold SLM, Kent T, Hogarth CA, Griswold MD, Amory JK, Isoherranen N (2015) Pharmacological inhibition of ALDH1A in mice decreases all-trans retinoic acid concentrations in a tissue specific manner. *Biochem Pharmacol* **95:** 177-192

Bushue N, Wan YJ (2010a) Retinoid pathway and cancer therapeutics. *Adv Drug Deliv Rev* **62:** 1285-1298

Bushue N, Wan YJY (2010b) Retinoid pathway and cancer therapeutics. *Adv Drug Deliv Rev* **62:** 1285-1298

Carlsen H, Ebihara K, Kuwata NH, Kuwata K, Aydemir G, Ruhl R, Blomhoff R (2021) A transgenic reporter mouse model for in vivo assessment of retinoic acid receptor transcriptional activation. *Int J Vitam Nutr Res*: 1-13

Chambon P (1996) A decade of molecular biology of retinoic acid receptors. *FASEB J* **10:** 940-954

Chassot AA, Le Rolle M, Jolivet G, Stevant I, Guigonis JM, Da Silva F, Nef S, Pailhoux E, Schedl A, Ghyselinck NB, Chaboissier MC (2020) Retinoic acid synthesis by ALDH1A proteins is dispensable for meiosis initiation in the mouse fetal ovary. *Sci Adv* **6:** eaaz1261

Cunningham TJ, Duester G (2015) Mechanisms of retinoic acid signalling and its roles in organ and limb development. *Nat Rev Mol Cell Biol* **16:** 110-123

Deltour L, Ang HL, Duester G (1996) Ethanol inhibition of retinoic acid synthesis as a potential mechanism for fetal alcohol syndrome. *FASEB J* **10:** 1050-1057

Ghyselinck NB, Duester G (2019) Retinoic acid signaling pathways. *Development* **146**

Gundersen TE (2006) Methods for detecting and identifying retinoids in tissue. *J Neurobiol* **66:** 631-644

Gutierrez-Mazariegos J, Schubert M, Laudet V (2014) Evolution of retinoic acid receptors and retinoic acid signaling. *Subcell Biochem* **70:** 55-73

Isoherranen N, Zhong G (2019) Biochemical and physiological importance of the CYP26 retinoic acid hydroxylases. *Pharmacol Ther* **204:** 107400

Kedishvili NY (2013) Enzymology of retinoic acid biosynthesis and degradation. *J Lipid Res* **54:** 1744-1760

Le HGT, Dowling JE, Cameron DJ (2012) Early retinoic acid deprivation in developing zebrafish results in microphthalmia. *Vis Neurosci* **29:** 219-228

le Maire A, Teyssier C, Balaguer P, Bourguet W, Germain P (2019) Regulation of RXR-RAR Heterodimers by RXR- and RAR-Specific Ligands and Their Combinations. *Cells* **8**

Morgenstern J, Fleming T, Kliemank E, Brune M, Nawroth P, Fischer A (2021) Quantification of All-Trans Retinoic Acid by Liquid Chromatography-Tandem Mass Spectrometry and Association with Lipid Profile in Patients with Type 2 Diabetes. *Metabolites* **11**

Rhinn M, Dollé P (2012) Retinoic acid signalling during development. *Development* **139:** 843-858

Rossant J, Zirngibl R, Cado D, Shago M, Giguere V (1991) Expression of a retinoic acid response element-hsplacZ transgene defines specific domains of transcriptional activity during mouse embryogenesis. *Genes Dev* **5:** 1333-1344

Shimozono S, Iimura T, Kitaguchi T, Higashijima S, Miyawaki A (2013) Visualization of an endogenous retinoic acid gradient across embryonic development. *Nature* **496:** 363-366

Solomin L, Johansson CB, Zetterstrom RH, Bissonnette RP, Heyman RA, Olson L, Lendahl U, Frisen J, Perlmann T (1998) Retinoid-X receptor signalling in the developing spinal cord. *Nature* **395:** 398-402

Wagner M, Han B, Jessell TM (1992) Regional differences in retinoid release from embryonic neural tissue detected by an in vitro reporter assay. *Development* **116:** 55-66

Event: 1882: Disrupted, initiation of meiosis of oogonia in the ovary

Short Name: Disrupted, meiotic initiation in oocyte

Key Event Component

Process	Object	Action
meiosis I	oocyte	disrupted

AOPs Including This Key Event

AOP ID and Name	Event Type
Aop:398 - Decreased ALDH1A (RALDH) activity leading to decreased fertility via disrupted meiotic initiation of fetal oogonia	KeyEvent

Stressors

Name
Acetaminophen
Indomethacin
Bis(2-ethylhexyl) phthalate
Bisphenol A

Biological Context

Level of Biological Organization
Cellular

Organ term

Organ term
ovary sex cord

Domain of Applicability

Taxonomic Applicability

Term	Scientific Term	Evidence	Links
mouse	Mus musculus	High	NCBI
human	Homo sapiens	High	NCBI

Term	Scientific Term	Evidence	Links
rat	Rattus norvegicus	High	NCBI

Life Stage Applicability

Life Stage	Evidence
Foetal	High
Development	High

Sex Applicability

Sex	Evidence
Female	High

Fetal oocytes need to enter meiosis prophase I to maintain the oocyte population and establish the oocyte pool. This process in conserved between mice, rats and humans.

Key Event Description

Oocyte meiosis

Oogonia, the female germ cells, are the precursors for the female oocytes. Primary oocytes are formed in the ovaries during fetal development when oogonia enter into prophase I of meiosis; meiotic entry initiates at around embryonic (E) day 13.5 in mice, E15.5 in rats, and gestational week 10-12 in humans. The entry into meiosis is driven by expression of the key genes *Stra8, Meiosin* and *Rec8* and is followed by expression of meiotic proteins including SYCP3 and γH2AX (Baltus et al, 2006; Bowles et al, 2006; Ishiguro et al, 2020; Kojima et al, 2019; Koubova et al, 2014; Spiller et al, 2017). The crucial role for *Stra8* in meiotic entry is conserved from mice to humans (Childs et al, 2011).

Disrupted meiotic entry as Key Event

The initiation of meiosis during fetal life is critical for maintenance of the oocytes throughout development and, eventually, for establishing the oocyte pool, or 'oocyte reserve' at birth. Without timely fetal entry into meiosis, the oogonia are depleted, as evidenced in *Stra8*-null mice (Baltus et al, 2006). The *Stra8*-null female mice are infertile and display abnormally small ovaries that are devoid of oocytes. For *Stra8* to be expressed and, therefore, for meiosis to initiate, the oogonia require direct stimulation by atRA as evidenced in mice (Bowles et al, 2016; Bowles et al, 2006; Feng et al, 2021; Koubova et al, 2006; Spiller et al, 2017; Teletin et al, 2017), and humans (Childs et al, 2011; Le Bouffant et al, 2010).

How it is Measured or Detected

There are no OECD-validated assays for measuring meiotic inhibition.

The expression of meiotic factors, such as STRA8, SYCP3, γH2AX, can be assessed at mRNA and/or protein levels and levels measured using primers/probes and antibodies that are commercially available.

Indirect measurements in animal models can be performed using the *Stra8* promoter element driving expression of reporter protein GFP (Feng et al, 2021). This reporter assay can detect the presence (GFP) or absence (GFP negative) of *Stra8* promoter activation in a semi-quantitative manner.

References

Baltus AE, Menke DB, Hu YC, Goodheart ML, Carpenter AE, de Rooij DG, Page DC (2006) In germ cells of mouse embryonic ovaries, the decision to enter meiosis precedes premeiotic DNA replication. *Nat Genet* **38:** 1430-1434

Bowles J, Feng CW, Miles K, Ineson J, Spiller C, Koopman P (2016) ALDH1A1 provides a source of meiosis-inducing retinoic acid in mouse fetal ovaries. *Nat Commun* **7:** 10845

Bowles J, Knight D, Smith C, Wilhelm D, Richman J, Mamiya S, Yashiro K, Chawengsaksophak K, Wilson MJ, Rossant J, Hamada H, Koopman P (2006) Retinoid signaling determines germ cell fate in mice. *Science* **312:** 596-600

Brieno-Enriquez MA, Reig-Viader R, Cabero L, Toran N, Martinez F, Roig I, Garcia Caldes M (2012) Gene expression is altered after bisphenol A exposure in human fetal oocytes in vitro. *Mol Hum Reprod* **18:** 171-183

Childs AJ, Cowan G, Kinnell HL, Anderson RA, Saunders PT (2011) Retinoic Acid signalling and the control of meiotic entry in the human fetal gonad. *PLoS One* **6:** e20249

Dean A, van den Driesche S, Wang Y, McKinnell C, Macpherson S, Eddie SL, Kinnell H, Hurtado-Gonzalez P, Chambers TJ, Stevenson K, Wolfinger E, Hrabalkova L, Calarrao A, Bayne RA, Hagen CP, Mitchell RT, Anderson RA, Sharpe RM (2016) Analgesic exposure in pregnant rats affects fetal germ cell development with inter-generational reproductive consequences. *Sci Rep* **6:** 19789

Feng CW, Burnet G, Spiller CM, Cheung FKM, Chawengsaksophak K, Koopman P, Bowles J (2021) Identification of regulatory elements required for Stra8 expression in fetal ovarian germ cells of the mouse. *Development* **148**

Holm JB, Mazaud-Guittot S, Danneskiold-Samsoe NB, Chalmey C, Jensen B, Norregard MM, Hansen CH, Styrishave B, Svingen T, Vinggaard AM, Koch HM, Bowles J, Koopman P, Jegou B, Kristiansen K, Kristensen DM (2016) Intrauterine Exposure to Paracetamol and Aniline Impairs Female Reproductive Development by Reducing Follicle Reserves and Fertility. *Toxicol Sci* **150:** 178-189

Ishiguro KI, Matsuura K, Tani N, Takeda N, Usuki S, Yamane M, Sugimoto M, Fujimura S, Hosokawa M, Chuma S, Ko MSH, Araki K, Niwa H (2020) MEIOSIN Directs the Switch from Mitosis to Meiosis in Mammalian Germ Cells. *Dev Cell* **52:** 429-445 e410

Kojima ML, de Rooij DG, Page DC (2019) Amplification of a broad transcriptional program by a common factor triggers the meiotic cell cycle in mice. *Elife* **8**

Koubova J, Hu YC, Bhattacharyya T, Soh YQ, Gill ME, Goodheart ML, Hogarth CA, Griswold MD, Page DC (2014) Retinoic acid activates two pathways required for meiosis in mice. *PLoS Genet* **10:** e1004541

Koubova J, Menke DB, Zhou Q, Capel B, Griswold MD, Page DC (2006) Retinoic acid regulates sex-specific timing of meiotic initiation in mice. *Proc Natl Acad Sci U S A* **103:** 2474-2479

Lawson C, Gieske M, Murdoch B, Ye P, Li Y, Hassold T, Hunt PA (2011) Gene expression in the fetal mouse ovary is altered by exposure to low doses of bisphenol A. *Biol Reprod* **84:** 79-86

Le Bouffant R, Guerquin MJ, Duquenne C, Frydman N, Coffigny H, Rouiller-Fabre V, Frydman R, Habert R, Livera G (2010) Meiosis initiation in the human ovary requires intrinsic retinoic acid synthesis. *Hum Reprod* **25:** 2579-2590

Liu JC, Lai FN, Li L, Sun XF, Cheng SF, Ge W, Wang YF, Li L, Zhang XF, De Felici M, Dyce PW, Shen W (2017) Di (2-ethylhexyl) phthalate exposure impairs meiotic progression and DNA damage repair in fetal mouse oocytes in vitro. *Cell Death Dis* **8:** e2966

Spiller C, Koopman P, Bowles J (2017) Sex Determination in the Mammalian Germline. *Annu Rev Genet* **51:** 265-285

Teletin M, Vernet N, Ghyselinck NB, Mark M (2017) Roles of Retinoic Acid in Germ Cell Differentiation. *Curr Top Dev Biol* **125:** 191-225

Zhang HQ, Zhang XF, Zhang LJ, Chao HH, Pan B, Feng YM, Li L, Sun XF, Shen W (2012) Fetal exposure to bisphenol A affects the primordial follicle formation by inhibiting the meiotic progression of oocytes. *Mol Biol Rep* **39:** 5651-5657

Zhang XF, Zhang T, Han Z, Liu JC, Liu YP, Ma JY, Li L, Shen W (2015) Transgenerational inheritance of ovarian development deficiency induced by maternal diethylhexyl phthalate exposure. *Reprod Fertil Dev* **27:** 1213-1221

Event: 1883: Decreased, size of the ovarian reserve

Short Name: Decreased, ovarian reserve

Key Event Component

Process	Object	Action
ovarian follicle development	ovarian follicle	decreased

AOPs Including This Key Event

AOP ID and Name	Event Type
Aop:398 - Decreased ALDH1A (RALDH) activity leading to decreased fertility via disrupted meiotic initiation of fetal oogonia	KeyEvent
Aop:563 - Aryl hydrocarbon Receptor (AHR) activation causes Premature Ovarian Insufficiency via Bax mediated apoptosis	KeyEvent

Stressors

Name
Diethylstilbestrol
Bisphenol A

Name
Genistein
Bis(2,4,6-trimethylphenyl)-lambda~2~-germane--selenium (1/1)

Biological Context

Level of Biological Organization
Organ

Organ term

Organ term
ovary sex cord

Domain of Applicability

Taxonomic Applicability

Term	Scientific Term	Evidence	Links
mouse	Mus musculus	High	NCBI
human	Homo sapiens	High	NCBI
rat	Rattus norvegicus	High	NCBI

Life Stage Applicability

Life Stage	Evidence
All life stages	Moderate

Sex Applicability

Sex	Evidence
Female	High

Follicle assembly occur in females during fetal life (humans) or around and after birth (rodents). Many of the mechanisms involved are preserved between mice, rats and humans.

Key Event Description

Formation of the follicle pool (follicle assembly)

During fetal life, primordial germ cells migrate to the genital ridges where they arrange into germ cell nests and proceed through to meiosis prophase I (Pepling & Spradling, 2001). Assembly into individual follicles occurs via mechanisms that are not well known, but involves germ cell nest break down and a reduction in oocyte numbers via programmed cell death. Somatic pre-granulosa cells infiltrate between the oocytes, arrange around them in a single layer, and establish what is called the primordial follicles (Escobar et al, 2008; Gawriluk et al, 2011; Pepling & Spradling, 2001). The primordial follicles constitute the follicle pool - a limited stock of oocytes that are available for maturation and potential fertilization determining the length of a female’s reproductive life span (Grive & Freiman, 2015).

The timing of follicle assembly differs between mammalian species, but the processes involved seem to be relatively well conserved (Grive & Freiman, 2015). In humans, follicle assembly occurs during mid-gestation whereas in mice and rats it is initiated around the time of birth and continues until approximately six days post partum.

Reduced follicle pool as Key Event

An intact follicle pool is critical for female fertility. Any disruption to the formation of the final pool can have adverse consequences for reproductive capacity, leading to sub- or infertility. Loss of oocytes/follicles can occur during any of the abovementioned stages during the process of follicle assembly – oocyte nest breakdown, programmed cell death or somatic pre-granulosa cell intrusion. Follicle assembly and establishment of the functional follicle pool is also dependent on the stages occurring before this process, e.g. migration of primordial germ cells to the genital ridges,

sex determination and meiosis.

How it is Measured or Detected

In animal studies, counting of follicles of different sizes is included in OECD guidelines: TG 416 (Two-Generation Reproductive Toxicity Study) and TG 443 (Extended One-Generation Reproductive Toxicity Study). It is a time-consuming and labor-intensive method and it is not recommended to compare values between studies (Tilly, 2003).

In humans, there is no direct way to count the follicle pool *in vivo*. Instead, surrogate markers are used. The most established biomarker for estimation of the follicle pool is anti-Müllerian hormone (AMH). It is readily measured in a blood sample and the levels are rather stable throughout the menstrual cycle (Broer et al, 2014).

The size of the pool can also be measured indirectly by mRNA and protein expression of meiotic markers, or by assessing overall ovary histology by histological assessments (Zhang et al, 2012).

References

Broer SL, Broekmans FJM, Laven JSE, Fauser BCJM (2014) Anti-Müllerian hormone: ovarian reserve testing and its potential clinical implications. *Hum Reprod Update* **20:** 688-701

Escobar ML, Echeverría OM, Ortíz R, Vázquez-Nin GH (2008) Combined apoptosis and autophagy, the process that eliminates the oocytes of atretic follicles in immature rats. *Apoptosis* **13:** 1253-1266

Gawriluk TR, Hale AN, Flaws JA, Dillon CP, Green DR, Rucker 3rd EB (2011) Autophagy is a cell survival program for female germ cells in the murine ovary. *Reproduction* **141:** 759-765

Grive KJ, Freiman RN (2015) The developmental origins of the mammalian ovarian reserve. *Development* **142:** 2554-2563

Jefferson W, Newbold R, Padilla-Banks E, Pepling M (2006) Neonatal genistein treatment alters ovarian differentiation in the mouse: inhibition of oocyte nest breakdown and increased oocyte survival. *Biol Reprod* **74:** 161-168

Mu X, Liao X, Chen X, Li Y, Wang M, Shen C, Zhang X, Wang Y, Liu X, He J (2015) DEHP exposure impairs mouse oocyte cyst breakdown and primordial follicle assembly through estrogen receptor-dependent and independent mechanisms. *Journal of Hazardous Materials* **298:** 232-240

Pepling ME, Spradling AC (2001) Mouse ovarian germ cell cysts undergo programmed breakdown to form primordial follicles. *Dev Biol* **234:** 339-351

Rodríguez HA, Santambrosio N, Santamaría CG, Muñoz-de-Toro M, Luque EH (2010) Neonatal exposure to bisphenol A reduces the pool of primordial follicles in the rat ovary. *Reprod Toxicol* **30:** 550-557

Tilly JL (2003) Ovarian follicle counts--not as simple as 1, 2, 3. *Reprod Biol Endocrinol* **1:** 11

Wang W, Hafner KS, Flaws JA (2014) In utero bisphenol A exposure disrupts germ cell nest breakdown and reduces fertility with age in the mouse. *Toxicol Appl Pharmacol* **276:** 157-164

Zhang HQ, Zhang XF, Zhang LJ, Chao HH, Pan B, Feng YM, Li L, Sun XF, Shen W (2012) Fetal exposure to bisphenol A affects the primordial follicle formation by inhibiting the meiotic progression of oocytes. *Mol Biol Rep* **39:** 5651-5657

Event: 405: disrupted, ovarian cycle

Short Name: disrupted, ovarian cycle

Key Event Component

Process	Object	Action
ovulation cycle	ovarian follicle	disrupted

AOPs Including This Key Event

AOP ID and Name	Event Type
Aop:7 - Aromatase (Cyp19a1) reduction leading to impaired fertility in adult female	AdverseOutcome
Aop:398 - Decreased ALDH1A (RALDH) activity leading to decreased fertility via disrupted meiotic initiation of fetal oogonia	KeyEvent

AOP ID and Name	Event Type
Aop:345 - Androgen receptor (AR) antagonism leading to decreased fertility in females	AdverseOutcome

Biological Context

Level of Biological Organization

Individual

Domain of Applicability

Taxonomic Applicability

Term	Scientific Term	Evidence	Links
mice	Mus sp.	Low	NCBI
rat	Rattus norvegicus	Moderate	NCBI

Life Stage Applicability

Life Stage	Evidence
Adult, reproductively mature	

Sex Applicability

Sex	Evidence
Female	High

The estrous cycle comprises the recurring physiologic changes that are induced by reproductive hormones in most mammalian females. Many of the mechanisms involved in the regulation of the reproductive axis are similar across species (particularly those mediated through the estrogen receptor), assessments of rodent estrous cyclicity can offer insight into potential adverse effects in humans (Goldman, Murr, & Cooper, 2007). While evaluations of vaginal cytology in the laboratory rodent can provide a valuable reflection of the integrity of the hypothalamic-pituitary-ovarian axis, other indices are more useful in humans to determine the functional status of the reproductive system (e.g. menses, basal body temperature, alterations in vaginal pH, cervical mucous viscosity, and blood hormone levels). Nevertheless, since many of the mechanisms involved in the regulation of the reproductive axis are similar across species (particularly those mediated through the estrogen receptor), assessments of rodent estrous cyclicity can offer insight into potential adverse effects in humans (Rasier, Toppari, Parent, & Bourguignon, 2006).

Key Event Description

Biological state

The female ovarian cycle is the result of a balanced cooperation between several organs and is determined by a complex interaction of hormones. Ovarian cycle irregularities include disturbances in the ovarian cycle (e.g. longer cycle, persistent estrus) and/or ovulation problems (deferred ovulation or anovulation). The estrous cycle (also oestrous cycle) comprises the recurring physiologic changes that are induced by reproductive hormones in females. Estrous cycles start after sexual maturity in females and are interrupted by anestrous phases or pregnancies. During this cycle numerous well defined and sequential alterations in reproductive tract histology, physiology and cytology occur, initiated and regulated by the hypothalamic-pituitary-ovarian (HPO) axis. The central feature of the mammalian estrous cycle is the periodic maturation of eggs that will be released at ovulation and luteinisation of the follicles after ovulation to form corpora lutea. Adapted from www.oecd.org/chemicalsafety/testing/43754807.pdf Biological compartments

The cyclic changes that occur in the female reproductive tract are initiated and regulated by the hypothalamic-pituitary-ovarian (HPO) axis. Although folliculogenesis occurs independently of hormonal stimulation up until the formation of early tertiary follicles, the gonadotrophins luteinising hormone (LH) and follicle stimulating hormone (FSH) are essential for the completion of follicular maturation and development of mature preovulatory (Graafian) follicles. The oestrous cycle consists of four stages: prooestrus, oestrus, metoestrus (or dioestrus 1) and dioestrus (or dioestrus 2) orchestrated by hormones. Levels of LH and FSH begin to increase just after dioestrus. Both hormones are secreted by the same secretory cells (gonadotrophs) in the pars distalis of the anterior pituitary (adenohypophysis). FSH stimulates the development of the zona granulosa and triggers expression of LH receptors by granulosa cells. LH initiates the synthesis and secretion of androstenedione and, to a lesser extent, testosterone by the theca interna; these androgens are utilised by granulosa cells as substrates in the synthesis of estrogen. Pituitary release of gonadotrophins thus drives follicular maturation and secretion of estrogen during prooestrus. Gonadotrophin secretion by the anterior pituitary is regulated by luteinising hormone-releasing hormone (LHRH), produced by the hypothalamus. LHRH is transported along the axons of hypothalamic neurones to the median eminence where it is secreted into the hypothalamic-hypophyseal portal system and transported to the anterior pituitary. The hypothalamus secretes LHRH in rhythmic pulses; this pulsatility is essential for the normal activation of

gonadotrophs and subsequent release of LH and FSH. Adapted from www.oecd.org/chemicalsafety/testing/43754807.pdf

Follicles that produce estrogens have sequestered pituitary FSH which in turn stimulates the aromatase reaction. Such follicles can undergo normal development and ovulation and contain eggs that readily resume meiosis when released. In the absence of an active local aromatase (i.e., no follicle-stimulating hormone), the follicles and oocytes become atretic and regress without ovulating. If aromatase is present, the estrogen and follicle stimulating hormone can further develop the follicular cells for normal luteal function after ovulation takes place (Ryan, 1982).

General role in biology

A sequential progression of interrelated physiological and behavioural cycles underlines the female's successful production of young. In many but not all species the first and most basic of these is estrous cycle, which is itself a combination of cycles.

How it is Measured or Detected

Methods that have been previously reviewed and approved by a recognized authority should be included in the Overview section above. All other methods, including those well established in the published literature, should be described here. Consider the following criteria when describing each method: 1. Is the assay fit for purpose? 2. Is the assay directly or indirectly (i.e. a surrogate) related to a key event relevant to the final adverse effect in question? 3. Is the assay repeatable? 4. Is the assay reproducible?

The pattern of events in the estrous cycle may provide a useful indicator of the normality of reproductive neuroendocrine and ovarian function in the nonpregnant female. It also provides a means to interpret hormonal, histologic, and morphologic measurements relative to stage of the cycle, and can be useful to monitor the status of mated females. Regular cyclicity is one of the key parameters in assessment of female reproductive function in rodents. Parameters assessed for cyclicity: - Number of cycling females - Number of females with regular cycles - Number of cycles - Estrous cycle length - Percentage of time spent in the various estrous cycle stages Estrous cyclicity provides a method for evaluating the endocrine disrupting activity of each test chemical under physiologic conditions where endogenous concentrations of estrogen vary. Abnormal cycles were defined as one or more estrous cycles in the 21-day period with prolonged estrus (≥3 days) and/or prolonged metestrus or diestrus (≥4 days) within a given cycle (Goldman, Murr, & Cooper, 2007).

Estrous cycle normality can be monitored in the rat and mouse by observing the changes in the vaginal smear cytology. Visual observation of the vagina is the quickest method, requires no special equipment, and is best used when only proestrus or estrus stages need to be identified. For details see: (Westwood, 2008), (Byers, Wiles, Dunn, & Taft, 2012) and OECD guidelines (www.oecd.org).

The observation that animals do not ovulate while exhibiting estrous cycles indicates that estrous cyclicity alone may not be a sufficient surrogate of healthy function of ovaries; the measurements of serum hormones and particularly FSH can contribute to more sensitivity indicators of healthy function of ovaries (Davis, Maronpot, & Heindel, 1994).

Monitoring of oestrus cyclicity is included in OECD test guidelines (Test No. 407: Repeated Dose 28-day Oral Toxicity Study in Rodents, 2008) [1], (Test No. 416: Two-Generation Reproduction Toxicity, 2001)[2] and (Test No. 443: Extended One-Generation Reproductive Toxicity Study, 2012) [3]and in USA EPA OCSPP 890.1450.

In vitro testing

The follicle culture models were developed for the in-vitro production of mature oocytes and used to study the process of folliculogenesis and oogenesis in vitro (Cortvrindt & Smitz, 2002). These in vitro cultures demonstrate near-identical effects to those found in vivo, therefore might be able to acquire a place in fertility testing, replacing some in-vivo studies for ovarian function and female gamete quality testing (Stefansdottir, Fowler, Powles-Glover, Anderson, & Spears, 2014).

Regulatory Significance of the AO

Chemicals may be found to interfere with reproductive function in the female rat. This interference is commonly expressed as a change in normal morphology of the reproductive tract or a disturbance in the duration of particular phases of the estrous cycle. This key event lies within the scope of testing for endocrine disrupting activity of chemicals and therefore for testing of female reproductive and developmental toxicity. Monitoring of oestrus cyclicity is included in OECD test guidelines (Test No. 407: Repeated Dose 28-day Oral Toxicity Study in Rodents, 2008), (Test No. 416: Two-Generation Reproduction Toxicity, 2001) and (Test No. 443: Extended One-Generation Reproductive Toxicity Study, 2012) and in USA EPA OCSPP 890.1450. While an evaluation of the estrous cycle in laboratory rodents can be a useful measure of the integrity of the hypothalamic-pituitary-ovarian reproductive axis, it can also serve as a way of insuring that animals exhibiting abnormal cycling patterns are excluded from a study prior to exposure to a test compound. When incorporated as an adjunct to other endpoint measures, a determination of a female's cycling status can contribute important information about the nature of a toxicant insult to the reproductive system. In doing so, it can help to integrate the data into a more comprehensive mechanistic portrait of the effect, and in terms of risk assessment, may provide some indication of a toxicant's impact on human reproductive physiology. Significant evidence that the estrous cycle (or menstrual cycle in primates) has been disrupted should be considered an adverse effect (OECD, 2008). Included should be evidence of abnormal cycle length or pattern, ovulation failure, or abnormal

menstruation.

References

Byers, S. L., Wiles, M. V, Dunn, S. L., & Taft, R. A. (2012). Mouse estrous cycle identification tool and images. PloS One, 7(4), e35538. doi:10.1371/journal.pone.0035538

Cortvrindt, R. G., & Smitz, J. E. J. (2002). Follicle culture in reproductive toxicology: a tool for in-vitro testing of ovarian function? Human Reproduction Update, 8(3), 243–54.

Davis, B. J., Maronpot, R. R., & Heindel, J. J. (1994). Di-(2-ethylhexyl) phthalate suppresses estradiol and ovulation in cycling rats. Toxicology and Applied Pharmacology, 128(2), 216–23. doi:10.1006/taap.1994.1200

Goldman, J. M., Murr, A. S., & Cooper, R. L. (2007). The rodent estrous cycle: characterization of vaginal cytology and its utility in toxicological studies. Birth Defects Research. Part B, Developmental and Reproductive Toxicology, 80(2), 84–97. doi:10.1002/bdrb.20106

OECD. (2008). No 43: Guidance document on mammalian reproductive toxicity testing and assessment.

Rasier, G., Toppari, J., Parent, A.-S., & Bourguignon, J.-P. (2006). Female sexual maturation and reproduction after prepubertal exposure to estrogens and endocrine disrupting chemicals: a review of rodent and human data. Molecular and Cellular Endocrinology, 254-255, 187–201. doi:10.1016/j.mce.2006.04.002

Ryan, K. J. (1982). Biochemistry of aromatase: significance to female reproductive physiology. Cancer Research, 42(8 Suppl), 3342s–3344s.

Stefansdottir, A., Fowler, P. A., Powles-Glover, N., Anderson, R. A., & Spears, N. (2014). Use of ovary culture techniques in reproductive toxicology. Reproductive Toxicology (Elmsford, N.Y.), 49C, 117–135. doi:10.1016/j.reprotox.2014.08.001

Test No. 407: Repeated Dose 28-day Oral Toxicity Study in Rodents. (2008). OECD Publishing. doi:10.1787/9789264070684-en

Test No. 416: Two-Generation Reproduction Toxicity. (2001). OECD Publishing. doi:10.1787/9789264070868-en

Test No. 443: Extended One-Generation Reproductive Toxicity Study. (2012). OECD Publishing. doi:10.1787/9789264185371-en

Westwood, F. R. (2008). The female rat reproductive cycle: a practical histological guide to staging. Toxicologic Pathology, 36(3), 375–84. doi:10.1177/0192623308315665

List of Adverse Outcomes in this AOP

Event: 406: decreased, Fertility

Short Name: decreased, Fertility

Key Event Component

Process	Object	Action
fertility		decreased
fertilization	fertility	decreased

AOPs Including This Key Event

AOP ID and Name	Event Type
Aop:7 - Aromatase (Cyp19a1) reduction leading to impaired fertility in adult female	AdverseOutcome
Aop:51 - PPARα activation leading to impaired fertility in adult male rodents	AdverseOutcome
Aop:18 - PPARα activation in utero leading to impaired fertility in males	AdverseOutcome
Aop:64 - Glucocorticoid Receptor (GR) Mediated Adult Leydig Cell Dysfunction Leading to Decreased Male Fertility	AdverseOutcome
Aop:348 - Inhibition of 11β-Hydroxysteroid Dehydrogenase leading to decreased population trajectory	KeyEvent
Aop:349 - Inhibition of 11β-hydroxylase leading to decresed population trajectory	KeyEvent

AOP ID and Name	Event Type
Aop:396 - Deposition of ionizing energy leads to population decline via impaired meiosis	KeyEvent
Aop:398 - Decreased ALDH1A (RALDH) activity leading to decreased fertility via disrupted meiotic initiation of fetal oogonia	AdverseOutcome
Aop:492 - Glutathione conjugation leading to reproductive dysfunction via oxidative stress	AdverseOutcome
Aop:345 - Androgen receptor (AR) antagonism leading to decreased fertility in females	AdverseOutcome

Biological Context

Level of Biological Organization

Individual

Domain of Applicability

Taxonomic Applicability

Term	Scientific Term	Evidence	Links
rat	Rattus norvegicus	High	NCBI
mouse	Mus musculus	High	NCBI
human	Homo sapiens	High	NCBI

Life Stage Applicability

Life Stage	Evidence
Adult, reproductively mature	High
Juvenile	High
Adults	High

Sex Applicability

Sex	Evidence
Male	High
Female	High

Plausible domain of applicability

Taxonomic applicability: The impaired fertility may also have relevance for fish, mammals, amphibians, reptiles, birds and and invertebrates with sexual reproduction.

Life stage applicability: The impaired fertility can be measured at juveniles and adults.

Sex applicability: The impaired fertility can be measured in both male and female species.

Key Event Description

Biological state

capability to produce offspring

Biological compartments

System

General role in biology

Fertility is the capacity to conceive or induce conception. Impairment of fertility represents disorders of male or female reproductive functions or capacity.

How it is Measured or Detected

As a measure, fertility rate, is the number of offspring born per mating pair, individual or population.

Regulatory Significance of the AO

Under REACH, information on reproductive toxicity is required for chemicals with an annual production/importation volume of 10 metric tonnes or more. Standard information requirements include a screening study on reproduction toxicity (OECD TG 421/422) at Annex VIII (10-100 t.p.a), a prenatal developmental toxicity study (OECD 414) on a first species at Annex IX (100-1000 t.p.a), and from March 2015 the OECD 443(Extended One-Generation Reproductive Toxicity Study) is reproductive toxicity requirement instead of the two generation reproductive toxicity study (OECD TG 416). If not conducted already at Annex IX, a prenatal developmental toxicity study on a second species at Annex X (≥ 1000 t.p.a.).

Under the Biocidal Products Regulation (BPR), information is also required on reproductive toxicity for active substances as part of core data set and additional data set (EU 2012, ECHA 2013). As a core data set, prenatal developmental toxicity study (EU TM B.31) in rabbits as a first species and a two-generation reproduction toxicity study (EU TM B.31) are required. OECD TG 443 (Extended One-Generation Reproductive Toxicity Study) shall be considered as an alternative approach to the multi-generation study.) According to the Classification, Labelling and Packaging (CLP) regulation (EC, 200; Annex I: 3.7.1.1): a) “reproductive toxicity” includes adverse effects on sexual function and fertility in adult males and females, as well as developmental toxicity in the offspring; b) “effects on fertility” includes adverse effects on sexual function and fertility; and c) “developmental toxicity” includes adverse effects on development of the offspring.

References

OECD (2001), *Test No. 416: Two-Generation Reproduction Toxicity*, OECD Guidelines for the Testing of Chemicals, Section 4, OECD Publishing, Paris, https://doi.org/10.1787/9789264070868-en.

OECD (2018), *Test No. 443: Extended One-Generation Reproductive Toxicity Study*, OECD Guidelines for the Testing of Chemicals, Section 4, OECD Publishing, Paris, https://doi.org/10.1787/9789264185371-en.

OECD (2018), *Test No. 414: Prenatal Developmental Toxicity Study*, OECD Guidelines for the Testing of Chemicals, Section 4, OECD Publishing, Paris, https://doi.org/10.1787/9789264070820-en.

OECD (2018), "Reproduction/Developmental Toxicity Screening Test (OECD TG 421) and Combined Repeated Dose Toxicity Study with the Reproduction/Developmental Toxicity Screening Test (OECD TG 422)", in *Revised Guidance Document 150 on Standardised Test Guidelines for Evaluating Chemicals for Endocrine Disruption*, OECD Publishing, Paris, https://doi.org/10.1787/9789264304741-25-en.

Appendix 2

List of Key Event Relationships in the AOP

List of Adjacent Key Event Relationships

Relationship: 2401: Decreased, ALDH1A activity leads to Decreased, atRA concentration

AOPs Referencing Relationship

AOP Name	Adjacency	Weight of Evidence	Quantitative Understanding
Decreased ALDH1A (RALDH) activity leading to decreased fertility via disrupted meiotic initiation of fetal oogonia	adjacent	High	Moderate
Inhibition of RALDH2 causes reduced all-trans retinoic acid levels, leading to transposition of the great arteries	adjacent	High	Moderate

Evidence Supporting Applicability of this Relationship

Taxonomic Applicability

Term	Scientific Term	Evidence	Links
human	Homo sapiens	High	NCBI
mouse	Mus musculus	High	NCBI
rat	Rattus norvegicus	High	NCBI

Life Stage Applicability

Life Stage	Evidence
All life stages	

Sex Applicability

Sex	Evidence
Male	High
Female	High

Key Event Relationship Description

All-trans retinoic acid (atRA) is the active metabolite of vitamin A in developing mammals and its physiological levels is tightly regulated by enzymatic pathways. This KER is particularly relevant for mammalian embryogenesis/fetal development stages.

atRA is synthesized from dietary vitamin A (retinol) by a two-step oxidation pathway(Chatzi et al, 2013; Kedishvili, 2016): 1) retinol dehydrogenase (RDH10) metabolizes retinol to retinaldehyde (reversible step), 2) retinaldehyde dehydrogenase ALDH1A (ALDH1A1, ALDH1A2, ALDH1A3) metabolizes retinaldehyde to RA (irreversible step). All three isoenzymes can carry out the second (irreversible step) to produce atRA, but ALDH1A2 is the most active form during development (Kedishvili, 2016). Thus, inhibition of ALDH1A2 during development will decrease atRA concentrations.

Evidence Supporting this KER

Evidence showing that retinaldehyde dehydrogenases is responsible for the irreversible oxidation of retinal to retinoic acid was provided by several studies in the 1960s, using calf and rat livers (Dmitrovskii, 1961; Dunagin Jr et al, 1964; Elder & Topper, 1962; Futterman, 1962; Lakshmanan et al, 1964; Mahadevan et al, 1962), as reviewed by (Kedishvili, 2016). The identification of the three isoenzymes ALDH1A1 (RALDH1), ALDH1A2 (RALDH2), ALDH1A3 (RALDH3) followed during 1980-1990 (Kedishvili, 2016). It is now considered canonical knowledge that the three retinaldehyde dehydrogenases are responsible for the in vivo biosynthesis of retinoic acid from retinal (Marchitti et al, 2008; Napoli, 2012).

Biological Plausibility

Embryogenesis/fetal development in mammals

Of the three isoenzymes, ALDH1A2 is the most active form during early development in mammals. This is evidenced in mice ablated for *Aldh1a2* (*Raldh2*$^{-/-}$), which are incapable of producing atRA and present with severe developmental defects (Niederreither et al, 1999). Conversely, mice lacking *Aldh1a1* or *Aldh1a3* survive fetal development, with phenotypes presenting postnatally (Dupé et al, 2003; Fan et al, 2003; Molotkov & Duester, 2003). Thus, the biological plausibility that inhibition of ALDH1A2 will lead to decreased atRA in cells and tissues during development is strong.

Empirical Evidence

The empirical evidence for linkage is strong and widely accepted. The enzymatic activity of ALDH1A2 and capacity to oxidize retinal has been proven *in vitro* (see KE 1880). In vivo, the strongest evidence comes from the *Aldh1a2*-deficient mice that fail to synthesize retinoic acid during embryogenesis (Niederreither et al, 1999). Additionally, ovary culture with the potent ALDH1A2 inhibitor WIN18,446 results in failure to upregulate the atRA-regulated gene *Stra8* in oocytes, resulting in germ cell loss (Rosario et al, 2020). Additional evidence for this relationship using WIN18,466 also comes from in vivo studies looking at spermatogenesis; inhibition of ALDH1A2 via WIN18,466 results in loss of atRA expression and halted spermatogenesis in diverse species such as mice, rabbits and zebrafish (Amory et al, 2011; Paik et al, 2014; Pradhan & Olsson, 2015).

Uncertainties and Inconsistencies

There are redundant pathways for atRA synthesis (e.g. ALDH isoforms) which may buffer a decrease in atRA concentrations caused by reduced ALDH1A activity, complicating the prediction of changes to atRA concentration. There is also tissue-specific expression of various components of the atRA synthesis pathways, which introduces additional variability in atRA concentration outcomes depending on biological context.

Quantitative Understanding of the Linkage

The distribution of retinoic acid in cells and tissues are highly variable, as has been shown across species including chicken (Maden et al, 1998), frogs (Chen et al, 1994), mice (Kane et al, 2005; Obrochta et al, 2014) and rats (Bhat, 1997), as well as serum/plasma from humans (Kane et al, 2008; Miyagi et al, 2001; Napoli et al, 1985).

The exact relationship between ALDH1A2 inhibition and resulting atRA concentrations in mammalian ovaries is unclear. The ALDH1A2 inhibitor WIN18,446 inhibits enzyme activity in vitro with an IC(50) of 0.3 µM (Amory et al, 2011), and a dose of only 0.01 µM is sufficient to significantly reduce expression of *Stra8* in cultured mouse fetal ovaries and with actual loss of oocytes from 2 µM (Rosario et al, 2020).

Time-scale

Since atRA must be enzymatically synthesized by ALDH1A enzymes (in this case ALDH1A2), the temporal and linear relationship between the two KEs are essential.

Known Feedforward/Feedback loops influencing this KER

Retinoic acid status is regulated by complex feedback loops. For instance, atRA induces expression of retinoid enzymes to promote synthesis of retinyl esters, but simultaneously atRA induces expression of its own catabolizing CYP26 enzymes (Kedishvili, 2013; Kedishvili, 2016; Teletin et al, 2017).

References

Amory JK, Muller CH, Shimshoni JA, Isoherranen N, Paik J, Moreb JS, Amory Sr DW, Evanoff R, Goldstein AS, Griswold MD (2011) Suppression of spermatogenesis by bisdichloroacetyldiamines is mediated by inhibition of testicular retinoic acid biosynthesis. *J Androl* **32:** 111-119

Bhat PV (1997) Tissue concentrations of retinol, retinyl esters, and retinoic acid in vitamin A deficient rats administered a single dose of radioactive retinol. *Can J Physiol Pharmacol* **75:** 74-77

Chatzi C, Cunningham TJ, Duester G (2013) Investigation of retinoic acid function during embryonic brain development using retinaldehyde-rescued Rdh10 knockout mice. *Dev Dyn* **242:** 1056-1065

Chen Y, Huang L, Solursh M (1994) A concentration gradient of retinoids in the early Xenopus laevis embryo. *Dev Biol* **161:** 70-76

Dmitrovskii AA (1961) Oxidation of vitamin A aldehyde to vitamin A acid catalyzed by aldehyde oxidase. *Biokhimiya* **26:** 126

Dunagin Jr PE, Zachman RD, Olson JA (1964) Identification of free and conjugated retinoic acid as a product of retinal (vitamin A aldehyde) metabolism in the rat in vivo. *Biochim Biophys Acta* **90:** 432-434

Dupé V, Matt N, Garnier JM, Chambon P, Mark M, Ghyselinck NB (2003) A newborn lethal defect due to inactivation of retinaldehyde dehydrogenase type 3 is prevented by maternal retinoic acid treatment. *Proc Natl Acad Sci U S A* **100:** 14036-14041

Elder TD, Topper YJ (1962) The oxidation of retinene (vitamin A1 aldehyde) to vitamin A acid by mammalian steroid-sensitive aldehyde dehydrogenase. *Biochim Biophys Acta* **64:** 430

Fan X, Molotkov A, Manabe SI, Donmoyer CM, Deltour L, Foglio MH, Cuenca AE, Blaner WS, Lipton SA, Duester G (2003) Targeted disruption of Aldh1a1 (Raldh1) provides evidence for a complex mechanism of retinoic acid synthesis in the developing retina. *Mol Cell Biol* **23:** 4637-4648

Futterman S (1962) Enzymatic oxidation of vitamin A aldehyde to vitamin A acid. *J Biol Chem* **237:** 677-680

Kane MA, Chen N, Sparks S, Napoli JL (2005) Quantification of endogenous retinoic acid in limited biological samples by LC/MS/MS. *Biochem J* **388:** 363-369

Kane MA, Folias AE, Napoli JL (2008) HPLC/UV quantitation of retinal, retinol, and retinyl esters in serum and tissues. *Anal Biochem* **378:** 71-79

Kedishvili NY (2013) Enzymology of retinoic acid biosynthesis and degradation. *J Lipid Res* **54:** 1744-1760

Kedishvili NY (2016) Retinoic Acid Synthesis and Degradation. *Subcell Biochem* **81:** 127-161

Lakshmanan MR, Vaidyanathan CS, Cama HR (1964) Oxidation of vitamin A1 aldehyde and vitamin A2 aldehyde to the corresponding acids by aldehyde oxidase from different species. *Biochem J* **90:** 569-573

Maden M, Sonneveld E, van der Saag PT, Gale E (1998) The distribution of endogenous retinoic acid in the chick embryo: implications for developmental mechanisms. *Development* **125:** 4133-4144

Mahadevan S, Murthy SK, Ganguly J (1962) Enzymic oxidation of vitamin A aldehyde to vitamin A acid by rat liver. *Biochem J* **85:** 326-331

Marchitti SA, Brocker C, Stagos D, Vasiliou V (2008) Non-P450 aldehyde oxidizing enzymes: the aldehyde dehydrogenase superfamily. *Expert Opin Drug Metab Toxicol* **4:** 697-720

Miyagi M, Yokoyama H, Shiraishi H, Matsumoto M, Ishii H (2001) Simultaneous quantification of retinol, retinal, and retinoic acid isomers by high-performance liquid chromatography with a simple gradiation. *J Chromatogr B Biomed Sci Appl* **757:** 365-368

Molotkov A, Duester G (2003) Genetic evidence that retinaldehyde dehydrogenase Raldh1 (Aldh1a1) functions downstream of alcohol dehydrogenase Adh1 in metabolism of retinol to retinoic acid. *J Biol Chem* **278:** 36085-36090

Napoli JL (2012) Physiological insights into all-trans-retinoic acid biosynthesis. *Biochim Biophys Acta* **1821:** 152-167

Napoli JL, Pramanik BC, Williams JB, Dawson MI, Hobbs PD (1985) Quantification of retinoic acid by gas-liquid chromatography-mass spectrometry: total versus all-trans-retinoic acid in human plasma. *J Lipid Res* **26:** 387-392

Niederreither K, Subbarayan V, Dollé P, Chambon P (1999) Embryonic retinoic acid synthesis is essential for early mouse post-implantation development. *Nat Genet* **21:** 444-448

Obrochta KM, Kane MA, Napoli JL (2014) Effects of diet and strain on mouse serum and tissue retinoid concentrations. *PLoS One* **9:** e99435

Paik J, Haenisch M, Muller CH, Goldstein AS, Arnold S, Isoherranen N, Brabb T, Treuting PM, Amory JK (2014) Inhibition of retinoic acid biosynthesis by the bisdichloroacetyldiamine WIN 18,446 markedly suppresses spermatogenesis and alters retinoid metabolism in mice. *J Biol Chem* **289:** 15104-15117

Pradhan A, Olsson PE (2015) Inhibition of retinoic acid synthesis disrupts spermatogenesis and fecundity in zebrafish. *Gen Comp Endocrinol* **217-218:** 81-91

Rosario R, Stewart HL, Walshe E, Anderson RA (2020) Reduced retinoic acid synthesis accelerates prophase I and follicle activation. *Reproduction* **160:** 331-341

Teletin M, Vernet N, Ghyselinck NB, Mark M (2017) Roles of Retinoic Acid in Germ Cell Differentiation. *Curr Top Dev Biol* **125:** 191-225

Relationship: 2477: Decreased, atRA concentration leads to Disrupted, meiotic initiation in oocyte

AOPs Referencing Relationship

AOP Name	Adjacency	Weight of Evidence	Quantitative Understanding
Decreased ALDH1A (RALDH) activity leading to decreased fertility via disrupted meiotic initiation of fetal oogonia	adjacent	Moderate	Low

Evidence Supporting Applicability of this Relationship

Taxonomic Applicability

Term	Scientific Term	Evidence	Links
mouse	Mus musculus	High	NCBI
human	Homo sapiens	Low	NCBI
rat	Rattus norvegicus	Moderate	NCBI

Life Stage Applicability

Life Stage	Evidence
Foetal	High

Sex Applicability

Sex	Evidence
Female	High

Key Event Relationship Description

All-trans retinoic acid (atRA) is the active metabolite of vitamin A and is involved in regulating a large number of developmental processes (Bushue & Wan, 2010; Ghyselinck & Duester, 2019). atRA is produced in spatial and temporal gradients, and these patterns are maintained by regulated expression of the synthesis and degradation enzymes of the atRA pathway (Kedishvili, 2013).The presence of atRA in the fetal ovaries induces germ cells to enter meiosis (Spiller et al, 2017). The initiation of meiosis at this time during fetal life is critical for maintenance of the germ line throughout development and establishment of the oocyte pool at birth. If atRA is not present at the correct time and at sufficient concentration, meiotic initiation is either delayed or prevented from occurring, ultimately disrupting germ cell development.

All-trans retinoic acid (atRA) is the active metabolite of vitamin A and is involved in regulating a large number of developmental processes (Bushue & Wan, 2010; Ghyselinck & Duester, 2019). atRA is produced in spatial and temporal gradients, and these patterns are maintained by regulated expression of the synthesis and degradation enzymes of the atRA pathway (Kedishvili, 2013).

Evidence Supporting this KER

The majority of evidence for this KER comes from rodent studies. In pregnant rats, depletion of vitamin A, the precursor of atRA, leads to an inability of ovarian germ cells to initiate meiosis (Li & Clagett-Dame, 2009). Further studies in mice have produced strong evidence that atRA acts as a meiosis-inducing factor in oogonia of the ovaries, although there are some conflicting data depending on which techniques are used (Griswold et al, 2012; Spiller &

Bowles, 2022). Evidence for the same mechanisms in human fetal ovaries is less substantiated and there may be species differences, particularly the manner in which atRA is made available (reviewed by (Jørgensen & Rajpert-De Meyts, 2014). In humans, evidence to support the KER comes from studies using explanted ovary culture.

Biological Plausibility

In mammalian germ cells, the initiation and progression of meiosis is critically dependent on the expression of Stimulated by retinoic acid gene 8 (*Stra8*). In mice, deleting *Stra8* leads to infertility in both males and females due to meiotic failure (Anderson et al, 2008; Baltus et al, 2006; Mark et al, 2008). What regulates the temporal expression of *Stra8,* and other factors (such as *Rec8* and *Dazl*) in the germ cells is not completely clear, but there is strong evidence to support an important role for atRA (Bowles et al, 2006; Feng et al, 2021; Griswold et al, 2012; Koubova et al, 2014; Soh et al, 2015).

In the fetal mouse ovary, entry into meiosis, preceded by Stra8 expression, occurs in an overlapping anterior-to-posterior wave from E12.5 (Bowles et al, 2006; Menke et al, 2003). Stra8 is also expressed in rat oogonia at comparative developmental stages to the mouse (Liu et al, 2020). atRA can similarly upregulate Stra8 in vitro, but this is restricted to pluripotent cell lines(Feng et al, 2021; Oulad-Abdelghani et al, 1996; Wang et al, 2016). Culture of mouse skin-derived stem cells with atRA stimulates the formation of functioning gametes and improves oogonia-like cells entry into meiosis (Dyce et al, 2018; Miyauchi et al, 2017). Stra8 expression cannot be induced by atRA in non-pluripotent cell lines, nor in somatic cells in vivo (Feng et al, 2021).

Exposure of pre-meiotic tammar (marsupial) ovaries to atRA induces Stra8 expression and oogonial meiotic entry (Hickford et al, 2017). Culturing fetal mouse ovaries in the presence of atRA increases the number of meiotic oocytes (Livera et al, 2000) and the same phenomenon is observed in cultured human fetal ovaries(Jørgensen et al, 2015).

In mouse ovaries lacking the atRA synthesizing enzyme ALDH1A1, the onset of germ cell meiosis is delayed(Bowles et al, 2016). This supports a previous study showing that atRA derived from the ovary (rather than mesonephros) is sufficient to initiate meiosis in mice (Mu et al, 2013). In humans, the local synthesis of atRA by ALDH1A enzymes within the ovary may also be involved in meiotic regulation (Childs et al, 2011; Le Bouffant et al, 2010). In two recent studies looking at mouse ovaries lacking all known atRA synthesizing enzymes (Chassot et al, 2020) or RA receptors (Vernet et al, 2020), expression of Stra8 was delayed, albeit some meiosis was still observed in these mice.

Empirical Evidence

Animal models

Model	Relevant observations	Reference
Vitamin A deficient (VAD) rats	Oocytes fail to enter meiosis in ovaries of VAT rats due to atAR deficiency.<br><br>Meiotic entry measured by SYCP3 expression was detected in 10% and 30% of germ cells in rats fed severely deficient (1.5ug of atRA per gram of diet) and moderately deficient (12ug of atRA per gram of diet) atRA diets, respectively, whilst controls had 70% of germ cells enter meiosis.<br><br>The expression of the atRA-responsive gene, Stra8, was reduced by approximately 90% and 50% in the severely and moderately atRA-deficient ovaries, respectively, compared with the atRA-sufficient controls.	(Li & Clagett-Dame, 2009)

In vitro/ex vivo

Study type	Species	Compound	Effect Dose	Duration	Results	Reference
Fetal ovaries in culture	Mouse	WIN 18,446<br><br>(ALDH1A2 inhibitor)	2 µM	3-12 d	Reduced *Stra8* expression and germ cell loss.	(Rosario et al, 2020)
Fetal ovaries in culture	Mouse	BMS-189453 (RAR antagonist)	1 µM	3 d	Reduced STRA8-positve germ cells without overall oocyte loss	(Minkina et al, 2017)

Embryonic stem cells	Mouse	ATRA	100 nM		Activates meiosis-related gene network	(Aoki & Takada, 2012)
Embryonic stem cells	Mouse	BMS-493 (RAR antagonist)	10 µM		Inhibition of expression meiosis-related genes	(Aoki & Takada, 2012)
Naked oocytes, matured	Mouse	ATRA	2 µM	24 h	Culture in presence of atRA increased meiosis resumption and formation of metaphase II oocytes	(Tahaei et al, 2011)
fetal ovaries in culture	Human	ATRA	1 µM	1-3 d	atRA strongly promote initiation of germ cell meiosis	(le Bouffant et al, 2010)
fetal ovaries in culture	Human	BMS-189453 (RAR antagonist)	10 µM	14 d	Partial inhibition of meiotic entry of germ cells	(le Bouffant et al, 2010)
fetal ovaries in culture	Human	Citral	55 µM	14 d	Partial inhibition of meiotic entry of germ cells by inhibiting RA synthesizing enzymes	(le Bouffant et al, 2010)
Fetal ovaries in culture	Mouse	AGN193109 (RAR antagonist)	5 µM	48 h or 72 h	Meiotic program inhibited	(Bowles et al, 2006)
Fetal ovaries in culture	Mouse	BMS-204493 (RAR antagonist)	5 µM	2 d	*Stra8* expression not upregulated in germ cells, marker for failed initiation of meiosis	(Koubova et al, 2006)
Fetal ovaries in culture	Mouse	ATRA	1 µM		Acceleration of germ cells into meiosis, reduction in total number of germ cells	(Livera et al, 2000)
Fetal ovaries in culture	Mouse	CD0336 (RARα agonist)	1 nM		Acceleration of germ cells into meiosis, reduction in total number of germ cells	(Livera et al, 2000)
Naked oocytes, matured	Camel	ATRA	20 µM	24 h	Stimulates meiosis and promotes oocyte viability	(Saddeldin et al, 2019)
Fetal ovaries in culture	Chicken	ATRA	1 µM		Stimulates meiotic initiation.	(Yu et al, 2013)

Uncertainties and Inconsistencies

Mouse deletion models for the atRA synthesis enzymes Aldh1a1, Aldh1a2 and Aldh1a3 showed decreased expression of Stra8 in double (Aldh1a2/3) and triple (Aldh1a1/2/3) knockouts, although ultimately some germ cells were observed undergoing meiosis in these ovaries, suggesting that atRA is not essential for meiotic onset or progression(Chassot et al, 2020; Kumar et al, 2011). Similarly, transgenic mice lacking the three atRA nuclear receptors (RAR-α, -β, -γ) showed reduced levels of Stra8, although ultimately some germ cells were observed undergoing meiosis and were capable of producing live offspring (Vernet et al, 2020). Whether or not these models led to impaired fertility (such as sub-fertility) has not been elucidated and the size of their oocyte pools were not determined. In addition, the completeness of the genetic deletions in these models is not clear (discussed in (Spiller & Bowles, 2022)).

Gain of function mouse ovary models for CYP26A1 and CYP26B1 show that CYP26B1 can prevent oocytes from entering meiosis (as assessed by failure to induce Stra8 expression), whereas CYP26A1 does not have the same effect despite being a potent atRA degrading enzyme. This suggests that CYP26B1 works by additional mechanism(s) other than RA degradation (Bellutti et al, 2019).

Quantitative Understanding of the Linkage

The quantitative knowledge pertaining to this KER is very limited as little is known about 1) the levels of endogenous atRA produced in the ovaries in different mammals and 2) the levels of atRA required to achieve meiotic initiation.

Response-response relationship

In vitro and ex vivo, it has been conclusively shown that low levels (as low as 1uM) of exogenous atRA can induce germ cells to enter meiosis in mice (Bowles et al, 2010) and rats (Livera et al, 2000) and, similarly, that it is necessary

to achieve meiosis in in vitro-derived oocytes via primordial germ cells (PGCs)/PGC-like cells (PGCLCs) (Miyauchi et al, 2017). Yet, its exact role in vivo is under debate.

Whilst the relative levels of endogenous atRA produced by the ovary (for any species) remains unknown, similarly, the quantitative relationship between atRA levels and induction of meiosis also remains unclear. As such, the quantitative understanding of how much atRA needs to be reduced to prevent germ cells to enter meiosis in vivo is rated low.

Time-scale

The time-scale for this KER is relatively short, limited to just a couple of days in e.g. mouse models. The induction of meiosis occurs shortly after the germ cells have colonized the ovary and occurs asynchronously (Bullejos & Koopman, 2004) (in mice this begins at E13.5 and is completed for all germ cells 2 days later at E15.5). Proliferation is halted and cells progress through leptonema, zygonema, pachynema, and arrest in diplonema of prophase I prior to birth (Zamboni, 1986). Time and duration of oogenesis varies between species, with rats the shortest duration of only 1-2 days, with other mammals such as pigs, cows, monkeys and humans lasting months (Peters, 1970).

The rat model of vitamin A deficiency (VAD) revealed severe defects to meiosis induction when Vitamin A was restricted/removed from the diet at E10.5, which is just 3 days prior to normal meiotic induction (Li & Clagett-Dame, 2009). Shorter time-frames have not been assessed to date, nor has rescue of VAD during later embryonic time-points been attempted.

Known modulating factors

No modulating factors are currently known to alter the quantitative relationship between the two KEs.

Known Feedforward/Feedback loops influencing this KER

During development, retinoic acid homeostasis is regulated by feedback loops, as both too much and too little RA can have deleterious effects on the embryo or fetus. The availability of atRA is regulated locally by maintaining a balance between synthesis (ALDH1 enzymes) and metabolism (CYP26 enzymes) (Kedishvili, 2013; Niederreither & Dollé, 2008; Roberts, 2020; Teletin et al, 2017).

The expression of *Aldh1a2* and *Cyp26a1* can act as part of a negative feedback loop in response to changes in RA levels. Exogenous atRA suppresses expression of *Aldh1a2* (Niederreither et al, 1997) whereas blocking atRA signalling increases expression of Aldh1a2. Although Cyp26 expression does not require atRA, addition of atRA greatly increases the expression of *Cyp26a1*, and conversely, reduced levels of atRA reduces *Cyp26a1* expression (de Roos et al, 1999; Hollemann et al, 1998; Ross & Zolfaghari, 2011; Sirbu et al, 2005). Negative feedback loops also extend to the enzymes that convert retinol to all-*trans* retinaldehyde as well as other related enzymes (Feng et al, 2010; Strate et al, 2009), including *Ski*, which seem to have cell-type specific roles(Melling et al, 2013; Niederreither & Dollé, 2008).

References

Anderson EL, Baltus AE, Roepers-Gajadien HL, Hassold TJ, de Rooij DG, van Pelt AMM, Page DC (2008) Stra8 and its inducer, retinoic acid, regulate meiotic initiation in both spermatogenesis and oogenesis in mice. *Proc Natl Acad Sci U S A* **105:** 14976-14980

Aoki T, Takada T (2012) Bisphenol A modulates germ cell differentiation and retinoic acid signaling in mouse ES cells. *Reprod Toxicol* **34:** 463-470

Baltus AE, Menke DB, Hu YC, Goodheart ML, Carpenter AE, de Rooij DG, Page DC (2006) In germ cells of mouse embryonic ovaries, the decision to enter meiosis precedes premeiotic DNA replication. *Nat Genet* **38:** 1430-1434

Bellutti L, Abby E, Tourpin S, Messiaen S, Moison D, Trautmann E, Guerquin MJ, Rouiller-Fabre V, Habert R, Livera G (2019) Divergent Roles of CYP26B1 and Endogenous Retinoic Acid in Mouse Fetal Gonads. *Biomolecules* **9:** 536

Bowles J, Feng CW, Miles K, Inseson J, Spiller CM, Koopman P (2016) ALDH1A1 provides a source of meiosis-inducing retinoic acid in mouse fetal ovaries. *Nat Commun* **7:** 10845

Bowles J, Feng CW, Spiller CM, Davidson TL, Jackson A, Koopman P (2010) FGF9 suppresses meiosis and promotes male germ cell fate in mice. *Dev Cell* **19:** 440-449

Bowles J, Knight D, Smith C, Wilhelm D, Richman J, Mamiya S, Yashiro K, Chawengsaksophak K, Wilson MJ, Rossant J, Hamada H, Koopman P (2006) Retinoid signaling determines germ cell fate in mice. *Science* **312:** 596-600

Bullejos M, Koopman P (2004) Germ cells enter meiosis in a rostro-caudal wave during development of the mouse ovary. *Mol Reprod Dev* **68:** 422-428

Bushue N, Wan YJY (2010) Retinoid pathway and cancer therapeutics. *Adv Drug Deliv Rev* **62:** 1285-1298

Chassot AA, Le Rolle M, Jolivet G, Stevant I, Guigonis JM, Da Silva F, Nef S, Pailhoux E, Schedl A, Ghyselinck NB, Chaboissier MC (2020) Retinoic acid synthesis by ALDH1A proteins is dispensable for meiosis initiation in the mouse fetal ovary. *Sci Adv* **6:** eaaz1261

Childs AJ, Cowan G, Kinnell HL, Anderson RA, Saunders PTK (2011) Retinoic Acid signalling and the control of meiotic entry in the human fetal gonad. *PLoS One* **6:** e20249

de Roos K, Sonneveld E, Compaan B, ten Berge D, Durston AJ, van der Saag PT (1999) Expression of retinoic acid 4-hydroxylase (CYP26) during mouse and Xenopus laevis embryogenesis. *Mech Dev* **82:** 205-211

Dyce PW, Tenn N, Kidder GM (2018) Retinoic acid enhances germ cell differentiation of mouse skin-derived stem cells. *J Ovarian Res* **11:** 19

Feng CW, Burnet G, Spiller CM, Cheung FKM, Chawengsaksophak K, Koopman P, Bowles J (2021) Identification of regulatory elements required for Stra8 expression in fetal ovarian germ cells of the mouse. *Development* **148:** dev194977

Feng L, Hernandez RE, Waxman JS, Yelon D, Moens CB (2010) Dhrs3a regulates retinoic acid biosynthesis through a feedback inhibition mechanism. *Dev Biol* **338:** 1-14

Ghyselinck NB, Duester G (2019) Retinoic acid signaling pathways. *Development* **146:** dev167502

Griswold MD, Hogarth CA, Bowles J, Koopman P (2012) Initiating meiosis: the case for retinoic acid. *Biol Reprod* **86:** 35

Hickford DE, Wong SFL, Frankenberg SR, Shaw G, Yu H, Chew KY, Renfree MB (2017) Expression of STRA8 is conserved in therian mammals but expression of CYP26B1 differs between marsupials and mice. *Biol Reprod* **97:** 217-229

Hollemann T, Chen Y, Grunz H, Pieler T (1998) Regionalized metabolic activity establishes boundaries of retinoic acid signalling. *EMBO J* **17:** 7361-7372

Jørgensen A, Nielsen JE, Perlman S, Lundvall L, Mitchell RT, Juul A, Rajpert-De Meyts E (2015) Ex vivo culture of human fetal gonads: manipulation of meiosis signalling by retinoic acid treatment disrupts testis development. *Hum Reprod* **30:** 2351-2363

Jørgensen A, Rajpert-De Meyts E (2014) Regulation of meiotic entry and gonadal sex differentiation in the human: normal and disrupted signaling. *Biomol Concepts* **5:** 331-341

Kedishvili NY (2013) Enzymology of retinoic acid biosynthesis and degradation. *J Lipid Res* **54:** 1744-1760

Koubova J, Hu YC, Bhattacharyya T, Soh YQS, Gill ME, Goodheart ML, Hogarth CA, Griswold MD, Page DC (2014) Retinoic acid activates two pathways required for meiosis in mice. *PLoS Genet* **10:** e1004541

Koubova J, Menke DB, Zhou Q, Capel B, Griswold MD, Page DC (2006) Retinoic acid regulates sex-specific timing of meiotic initiation in mice. *Proc Natl Acad Sci U S A* **103:** 2474-2479

Kumar S, Chatzi C, Brade T, Cunningham TJ, Zhao X, Duester G (2011) Sex-specific timing of meiotic initiation is regulated by Cyp26b1 independent of retinoic acid signalling. *Nat Commun* **2:** 151

Le Bouffant R, Guerquin MJ, Duquenne C, Frydman N, Coffigny H, Rouiller-Fabre V, Frydman R, Habert R, Livera G (2010) Meiosis initiation in the human ovary requires intrinsic retinoic acid synthesis. *Hum Reprod* **25:** 2579-2590

Li H, Clagett-Dame M (2009) Vitamin A deficiency blocks the initiation of meiosis of germ cells in the developing rat ovary in vivo *Biol Reprod* **81:** 996-1001

Liu Y, Fan X, Yue M, Yue W, Zhang X, Zhang J, Ren G, He J (2020) Expression and localization of meiosis-associated protein in gonads of female rats at different stages. *Acta Histochemica* **122:** 151509

Livera G, Rouiller-Fabre V, Valla J, Habert R (2000) Effects of retinoids on the meiosis in the fetal rat ovary in culture.*Mol Cell Endocrinol* **165:** 225-231

Mark M, Jacobs H, Oulad-Abdelghani M, Dennefeld C, Féret B, vernet N, Codreanu CA, Chambon P, Ghyselinck NB (2008) STRA8-deficient spermatocytes initiate, but fail to complete, meiosis and undergo premature chromosome condensation. *J Cell Sci* **121:** 3233-3242

Melling MA, Friendship CR, Shepherd TG, Drysdale TA (2013) Expression of Ski can act as a negative feedback mechanism on retinoic acid signaling *Dev Dyn* **242:** 604-613

Menke DB, Koubova J, Page DC (2003) Sexual differentiation of germ cells in XX mouse gonads occurs in an anterior-to-posterior wave. *Dev Biol* **262:** 303-312

Minkina A, Lindeman RE, Gearhart MD, Chassot AA, Chaboissier MC, Ghyselinck NB, Bardwell VJ, Zarkower D (2017) Retinoic acid signaling is dispensable for somatic development and function in the mammalian ovary. *Dev Biol* **424:** 208-220

Miyauchi H, Ohta H, Nagaoka S, Nakaki F, Sasaki K, Hayashi K, Yabuta Y, Nakamura T, Yamamoto T, Saitou M (2017) Bone morphogenetic protein and retinoic acid synergistically specify female germ-cell fate in mice. *EMBO J* **36:** 3100-3119

Mu X, Wen J, Guo M, Wang J, Li G, Wang Z, Teng Z, Cui Y, Xia G (2013) Retinoic acid derived from the fetal ovary initiates meiosis in mouse germ cells. *J Cell Physiol* **228:** 627-639

Niederreither K, Dollé P (2008) Retinoic acid in development: towards an integrated view. *Nat Rev Genet* **9:** 541-553

Niederreither K, McCaffery P, Dräger UC, Chambon P, Dollé P (1997) Restricted expression and retinoic acid-induced downregulation of the retinaldehyde dehydrogenase type 2 (RALDH-2) gene during mouse development. *Mech Dev* **62:** 67-78

Oulad-Abdelghani M, Bouillet P, Décimo D, Gansmuller A, Heyberger S, Dollé P, Bronner S, Lutz Y, Chambon P (1996) Characterization of a premeiotic germ cell-specific cytoplasmic protein encoded by Stra8, a novel retinoic acid-responsive

gene. *J Cell Biol* **135:** 469-477

Peters H (1970) Migration of gonocytes into the mammalian gonad and their differentiation. *Philos Trans R Soc Lond B Biol Sci* **259:** 91-101

Roberts C (2020) Regulating Retinoic Acid Availability during Development and Regeneration: The Role of the CYP26 Enzymes. *J Dev Biol* **8:** 6

Rosario R, Stewart HL, Walshe E, Anderson RA (2020) Reduced retinoic acid synthesis accelerates prophase I and follicle activation. *Reproduction* **160:** 331-341

Ross AC, Zolfaghari R (2011) Cytochrome P450s in the regulation of cellular retinoic acid metabolism. *Annu Rev Nutr* **31:** 65-87

Saddeldin IM, Swelum AA, Elsafadi M, Mahmood A, Yaqoob SH, Alfayez M, Alowaimer AN (2019) Effects of all-trans retinoic acid on the in vitro maturation of camel (Camelus dromedarius) cumulus-oocyte complexes. *J Reprod Dev* **65:** 215-221

Sirbu IO, Gresh L, Barra J, Duester G (2005) Shifting boundaries of retinoic acid activity control hindbrain segmental gene expression. *Development* **132:** 2611-2622

Soh YQS, Junker JP, Gill ME, Mueller JL, van Oudenaarden A, Page DC (2015) A Gene Regulatory Program for Meiotic Prophase in the Fetal Ovary. *PLoS Genet* **11:** e1005531

Spiller C, Koopman P, Bowles J (2017) Sex Determination in the Mammalian Germline. *Annu Rev Genet* **51:** 265-285

Spiller CM, Bowles J (2022) Instructing mouse germ cells to adopt a female fate. *Sex Dev* **In Press**

Strate I, Min TH, Iliev D, Pera EM (2009) Retinol dehydrogenase 10 is a feedback regulator of retinoic acid signalling during axis formation and patterning of the central nervous system. *Development* **136:** 461-472

Tahaei LS, Eimani H, Yazdi PE, Ebrahimi B, Fathi R (2011) Effects of retinoic acid on maturation of immature mouse oocytes in the presence and absence of a granulosa cell co-culture system. *J Assist Reprod Genet* **28:** 553-558

Teletin M, Vernet N, Ghyselinck NB, Mark M (2017) Roles of Retinoic Acid in Germ Cell Differentiation. *Curr Top Dev Biol* **125:** 191-225

Vernet N, Condrea D, Mayere C, Féret B, Klopfenstein M, Magnant W, Alunni V, Teletin M, Souali-Crespo S, Nef S, Mark M, Ghyselinck NB (2020) Meiosis occurs normally in the fetal ovary of mice lacking all retinoic acid receptors. *Sci Adv* **6:** eaaz1139

Wang S, Wang X, Ma L, Lin X, Zhang D, Li Z, Wu Y, Zheng C, Feng X, Liao S, Feng Y, Chen J, Hu X, Wang M, Han C (2016) Retinoic Acid Is Sufficient for the In Vitro Induction of Mouse Spermatocytes. *Stem Cell Reports* **7:** 80-94

Yu M, Yu P, Leghari IH, Ge C, Mi Y, Zhang C (2013) RALDH2, the enzyme for retinoic acid synthesis, mediates meiosis initiation in germ cells of the female embryonic chickens *Amino Acids* **44:** 405-412

Zamboni L (1986) Meiosis as a sexual dimorphic character of germinal cell differentiation. *Tokai J Exp Clin Med* **11:** 377-390

Relationship: 2481: Disrupted, meiotic initiation in oocyte leads to Decreased, ovarian reserve

AOPs Referencing Relationship

AOP Name	Adjacency	Weight of Evidence	Quantitative Understanding
Decreased ALDH1A (RALDH) activity leading to decreased fertility via disrupted meiotic initiation of fetal oogonia	adjacent	High	Moderate

Evidence Supporting Applicability of this Relationship

Taxonomic Applicability

Term	Scientific Term	Evidence	Links
human	Homo sapiens	High	NCBI
mouse	Mus musculus	High	NCBI
rat	Rattus norvegicus	High	NCBI

Life Stage Applicability

Life Stage	Evidence
Foetal	High

Sex Applicability

Sex	Evidence
Female	High

Female mammals during fetal life, as this corresponds to initiation of meiosis prophase I in oocytes of the ovaries.

Key Event Relationship Description

The establishment of the primordial follicle pool is a multistep process that spans from early fetal life to reproductive maturity. This period of time varies greatly between species, lasting only a few weeks in mice and rats, but years in humans (Tingen et al, 2009). One important process is for the mitotic primordial germs cells to enter meiosis prior to cyst formation(Findlay et al, 2015; Tingen et al, 2009). Notably, in females there is a massive loss of oocytes between cyst formation and time of maturity, and the exact mechanisms behind this oocyte degradation is not well understood (Findlay et al, 2015; Sun et al, 2017).

Evidence Supporting this KER

It is well established that disruption to meiosis during oocyte development can lead to sub-/infertility in females at reproductive age. There are numerous gene mutation in mice showing links between meiotic defects and fertility phenotypes, as well as associations to female fertility phenotypes in humans (Adelfalk et al, 2011).

Biological Plausibility

Although the entry into meiosis is required for oocyte development, the relationship between meiotic entry and final oocyte reserve remains unclear. However, there are strong correlations between disrupted meiosis and infertility (or aneuploidy) in females (Handel & Schimenti, 2010). For instance, in mice, ablation of *Stra8* prevents oocytes from entering meiosis in the fetal ovaries and mature females are infertile (Baltus et al, 2006; Zhou et al, 2008). Mutation in *Atm*, a gene involved in recombination during meiosis, results in complete loss of primary oocytes in mice, and greatly reduced follicle pool in humans (Adelfalk et al, 2011; Agamanolis & Greenstein, 1979; Aguilar et al, 1968; Xu et al, 1996). Other examples include *Fanca* and *Fancd2* genes that are involved in recombination. Mutations to these genes lead to oocyte degeneration and subfertility in mice (Cheng et al, 2000; Houghtaling et al, 2003; Wong et al, 2003).

Mice with *Lhx8* ablation display total loss of oocytes. *Lhx8-/-* mice maintain oocytes during fetal development, but loose the oocytes shortly after birth by autophagy, likely because the oocytes have failed to enter meiosis in utero (Choi et al, 2008; D'Ignazio et al, 2018). *Fzr1* is a regulator of mitotic cell division. When conditionally ablated from the germ cells, female mice display premature ovarian failure by 5 months of age and are subfertile; oocytes are lost in utero during early meiotic prophase I (Holt et al, 2014).

CYP51 (lanosterol 14 α-demethylase) is expressed by fetal oocytes and is involved in meiotic regulation(Mu et al, 2018). Inhibition of CYP51 activity reduces the formation of primordial follicles (Zhang et al, 2009) by disrupting entry into diplotene stage (Mu et al, 2018).

Empirical Evidence

Study type	Species	Compound	Effect Dose	Duration	Results	Reference
In vivo	Mouse	di(2-ethylhexyl) phthalate (DEHP)			Delayed meiotic progression at 17.5 dpc (reduced *Stra8* expression at time of initiation, 13.5 dpc)	(Zhang et al, 2015)
In vivo	Mouse	Bisphenol A (BPA)	0.08 mg/kg/d	12.5-17.5 dpc	Delayed meiotic progression at 17.5 dpc (incl. decreased Stra8 expression)	(Zhang et al, 2012)
In vivo	Mouse	Paracetamol	350 mg/kg/d	13.5-21.5 dpc	Delayed meiotic entry and reduced fertility	(Dean et al, 2016)
In vivo	Mouse	Indomethacin	0.8 mg/kg/d	15.5-18.5 dpc	Delayed meiotic entry and reduced fertility	(Dean et al, 2016)
fetal ovary culture	Mouse	RS21745 (CYP51 inhibitor)	10 µM	3 days	Oocytes arrest at zygote stage (delayed meiotic progression).	(Mu et al, 2018)
fetal ovary culture	Mouse	RS21745 (CYP51 inhibitor)	1, 25, 50 µM	2 days exposure + 5 days no exposure	Dose dependent reduction in follicle numbers; significant in 25 and 50 µM groups	(Zhang et al, 2009)

Uncertainties and Inconsistencies

The mechanisms and outcomes of meiosis I disruption may vary significantly across species, making it challenging to generalize findings from animal models to humans. Also, the extent of disruption that is required to significantly affect the ovarian reserve remains uncertain, as there may be potential threshold effects influenced by genetic, epigenetic, and environmental factors.

Timing of disruption to meiosis I initiation in oocytes may influence to what extent the ovary reserve is ultimately affected.

Methods to quantify the ovarian reserve (e.g. follicle count, AMH levels) may not directly reflect the impact of meiotic disruption, leading to inconsistencies in observed effects.

Quantitative Understanding of the Linkage

The quantitative understanding of this KER remains poorly understood, not least because the quantification of actual oocyte numbers at various stages of development are very difficult to perform.

Response-response relationship

The ovarian follicle pool (ovarian reserve) refers to the final number of primordial follicles in the mature ovary and is established through a series of events. In most mammals, it is determined during gestation or just after birth and relies on i) how many germ cells were established during embryogenesis, ii) their proliferation during migration and early ovary development, iii) death rate during oogenesis and iv) formation of primordial follicles at nest breakdown (Findlay et al, 2015). The last two stages, which includes nest formation and breakdown, is largely influenced by the mitotic-meiotic transition, in that oocytes that have failed to enter meiosis may contribute to the cysts population, but only high quality oocytes in meiotic prophase are spared during cyst breakdown (Findlay et al, 2015). Thus, there is a response-response relationship between meiotic entry and final follicle pool, albeit the quantitative relationship is not that well understood.

Time-scale

The time-scale for oocyte mitotic-meiotic transition and subsequent nest breakdown varies between species, but generally takes place from mid gestation to around the time of birth. In mice, meiosis and nest formation is initiated from around E13, whereas in humans it initiates at around GW12-14 (Childs et al, 2012; Findlay et al, 2015; Grive & Freiman, 2015; Pepling, 2006; Tingen et al, 2009). Nest breakdown starts just before birth in mice and completes around postnatal day 5 (Grive & Freiman, 2015; Pepling, 2006). In humans, nest breakdown takes place during second trimester (Grive & Freiman, 2015; Tingen et al, 2009).

Known modulating factors

Variations in genes involved in meiotic regulation (e.g., SYCP3, MSH5, DAZL) may influence sensitivity to disruptions, including species differences, as may variations in epigenetic status of oocytes.

Known Feedforward/Feedback loops influencing this KER

No (known) relevant feedback loop.

References

Adelfalk C, Ahmed EA, Scherthan H (2011) Reproductive Phenotypes of Mouse Models Illuminate Human Infertility. *J Reproduktionsmed Endokrinol* **8:** 376-383

Agamanolis DP, Greenstein JI (1979) Ataxia-telangiectasia. Report of a case with Lewy bodies and vascular abnormalities within cerebral tissue. *J Neuropathol Exp Neurol* **38:** 475-489

Aguilar MJ, Kamoshita S, Landing BH, Boder E, Sedgwick RP (1968) Pathological observations in ataxia-telangiectasia. A report of five cases. *J Neuropathol Exp Neurol* **27:** 659-676

Baltus AE, Menke DB, Hu YC, Goodheart ML, Carpenter AE, de Rooij DG, Page DC (2006) In germ cells of mouse embryonic ovaries, the decision to enter meiosis precedes premeiotic DNA replication. *Nat Genet* **38:** 1430-1434

Cheng NC, van de Vrugt HJ, van der Valk MA, Oostra AB, Krimpenfort P, de Vries Y, Joenje H, Berns A, Arwert F (2000) Mice with a targeted disruption of the Fanconi anemia homolog Fanca. *Hum Mol Genet* **9:** 1805-1811

Childs AJ, Kinnell HL, He J, Anderson RA (2012) LIN28 is selectively expressed by primordial and pre-meiotic germ cells in the human fetal ovary. *Stem Cells Dev* **21:** 2343-2349

Choi Y, Ballow DJ, Xin Y, Rajkovic A (2008) Lim homeobox gene, lhx8, is essential for mouse oocyte differentiation and survival. *Biol Reprod* **79:** 442-449

D'Ignazio L, Michel M, Beyer M, Thompson K, Forabosco A, Schlessinger D, Pelosi E (2018) Lhx8 ablation leads to massive autophagy of mouse oocytes associated with DNA damage. *Biol Reprod* **98:** 532-542

Dean A, van den Driesche S, Wang Y, McKinnell C, Macpherson S, Eddie SL, Kinnell HL, Hurtado-Gonzalez P, Chambers TJ,

Stevenson K, Wolfinger E, Hrabalkova L, Calarrao A, Bayne RA, Hagen CP, Mitchell RT, Anderson RA, Sharpe RM (2016) Analgesic exposure in pregnant rats affects fetal germ cell development with inter-generational reproductive consequences. *Sci Rep* **6:** 19789

Findlay JK, Hutt KJ, Hickey M, Anderson RA (2015) How Is the Number of Primordial Follicles in the Ovarian Reserve Established? *Biol Reprod* **93:** 111

Grive KJ, Freiman RN (2015) The developmental origins of the mammalian ovarian reserve. *Development* **142:** 2554-2563

Handel MA, Schimenti JC (2010) Genetics of mammalian meiosis: regulation, dynamics and impact on fertility. *Nat Rev Genet* **11:** 124-136

Holt JE, Pye V, Boon E, Stewart JL, García-Higuera I, Moreno S, Rodríguez R, Jones KT, McLaughlin EA (2014) The APC/C activator FZR1 is essential for meiotic prophase I in mice. *Development* **141:** 1354-1365

Houghtaling S, Timmers C, Noll M, Finegold MJ, Jones SN, Meyn MS, Grompe M (2003) Epithelial cancer in Fanconi anemia complementation group D2 (Fancd2) knockout mice. *Genes Dev* **17:** 2021-2035

Mu X, Wen J, Chen Q, Wang Z, Wang Y, Guo M, Yang Y, Xu J, Wei Z, Xia G, Yang M, Wang C (2018) Retinoic acid-induced CYP51 nuclear translocation promotes meiosis prophase I process and is correlated to the expression of REC8 and STAG3 in mice. *Biol Open* **7:** bio035626

Pepling ME (2006) From primordial germ cell to primordial follicle: mammalian female germ cell development. *Genesis* **44:** 622-632

Sun YC, Sun XF, Dyce PW, Shen W, Chen H (2017) The role of germ cell loss during primordial follicle assembly: a review of current advances. *Int J Biol Sci* **13:** 449-457

Tingen C, Kim A, Woodruff TK (2009) The primordial pool of follicles and nest breakdown in mammalian ovaries. *Mol Hum Reprod* **15:** 795-803

Wong JCY, Alon N, Mckerlie C, Huang JR, Meyn MS, Buchwald M (2003) Targeted disruption of exons 1 to 6 of the Fanconi Anemia group A gene leads to growth retardation, strain-specific microphthalmia, meiotic defects and primordial germ cell hypoplasia. *Hum Mol Genet* **12:** 2063-2076

Xu Y, Ashley T, Brainerd EE, Bronson RT, Meyn MS, Baltimore D (1996) Targeted disruption of ATM leads to growth retardation, chromosomal fragmentation during meiosis, immune defects, and thymic lymphoma. *Genes Dev* **10:** 2411-2422

Zhang H, Xu B, Xie H, Zhou B, Quyang H, Ning G, Li G, Zhang M (2009) Lanosterol metabolic product(s) is involved in primordial folliculogenesis and establishment of primordial follicle pool in mouse fetal ovary. *Mol Reprod Dev* **76:** 514-521

Zhang HQ, Zhang XF, Zhang LJ, Chao HH, Pan B, Feng YM, Li L, Sun XF, Shen W (2012) Fetal exposure to bisphenol A affects the primordial follicle formation by inhibiting the meiotic progression of oocytes. *Mol Biol Rep* **39:** 5651-5657

Zhang XF, Zhang T, Han Z, Liu JC, Liu YP, Ma JY, Li L, Shen W (2015) Transgenerational inheritance of ovarian development deficiency induced by maternal diethylhexyl phthalate exposure. *Reprod Fertil Dev* **27:** 1213-1221

Zhou Q, Nie R, Li Y, Friel P, Mitchell D, Hess RA, Small C, Griswold MD (2008) Expression of Stimulated by Retinoic Acid Gene 8 (Stra8) in Spermatogenic Cells Induced by Retinoic Acid: An In Vivo Study in Vitamin A-Sufficient Postnatal Murine Testes. *Biol Reprod* **79:** 35-42

Relationship: 2525: Decreased, ovarian reserve leads to disrupted, ovarian cycle

AOPs Referencing Relationship

AOP Name	Adjacency	Weight of Evidence	Quantitative Understanding
Decreased ALDH1A (RALDH) activity leading to decreased fertility via disrupted meiotic initiation of fetal oogonia	adjacent	Moderate	Low

Evidence Supporting Applicability of this Relationship

Taxonomic Applicability

Term	Scientific Term	Evidence	Links
human, mouse, rat	human, mouse, rat	High	NCBI

Life Stage Applicability

Life Stage	Evidence

Life Stage	Evidence
Adult, reproductively mature	High

Sex Applicability

Sex	Evidence
Female	High

Applicable for (mammalian) females during postnatal life. Although diminished ovary reserve was caused by disruption during fetal development, the link between reduced ovary reserve and irregular cycling occurs postnatally during reproductive ages.

Key Event Relationship Description

Reduced ovarian reserve, meaning the finite pool of primordial follicles containing the immature oocytes, is leading to ovarian cycle irregularities. Cycle irregularities include disturbances of the ovarian cycle like shorter cycle and prolonged estrus and/or ovulation problems like deferred ovulation and anovulation. This KER is considered canonical information.

Evidence Supporting this KER

Biological Plausibility

All primordial follicles are formed during early development (fetal period in humans, perinatally in rodents) and can stay dormant for long periods of time (years in humans, months in rodents). The stock of primordial dormant follicles constitutes the ‘ovarian reserve’. In humans, millions of follicles are formed by mid-gestation (Wallace & Kelsey, 2010). Upon puberty, the hypothalamus-pituitary-ovary (HPO) axis matures enabling the primordial follicles to grow into maturity in a process called folliculogenesis, which serves two functions: i) secretion of steroid hormones that enable pregnancy, and ii) production of mature oocytes that are ovulated for possible fertilization.

Cohorts of primordial follicles continuously enter the growing pool. After puberty, growing antral follicles are recruited for final maturation during each menstrual cycle (estrus cycle in rodents) by gonadotropins secreted from the pituitary gland but only a limited number will reach maturity and ovulate oocytes (typically one in humans and 10-20 in rodents). The majority of follicles never reach maturity and instead die in a process called atresia. Therefore, the ovarian reserve is irreversibly depleted with age. When the reserve is depleted to a level that cannot faithfully maintain steroid production, fertility ceases. In humans, fertility ends in sterility at menopause when less than 1,000 follicles remain (Wallace & Kelsey, 2010).

Regular cycles are considered as an indicator of reproductive health and often used in animal studies as the earliest biomarker to reflect disruption of fertility and ovotoxicity (Hooser et al, 1994). OECD test guidelines 407, 416 and 443 include rodent cyclicity as an endpoint to assess reproductive toxicity (OECD, 2001; OECD, 2008; OECD, 2018). In humans, normal menstrual cycle lasts 28-35 days, and in rodents 4-6 days. When the ovarian reserve is depleted to a critically low level, like naturally during the perimenopausal period in humans, the variability of the cycle length increases with many of the cycles being anovulatory. The lower the ovarian reserve is, the lower the probability of growing follicles to exist at any given time point. Since the growing follicles produce steroid hormones that are essential for cyclicity, the lack of growing follicles leads to disturbances of the HPO axis. Therefore, the lower the ovarian reserve is, the less probable are regular cycles; reflected by regular menstruation in humans (O'Connor et al, 1998; O'Connor et al, 2001).

Supporting evidence exists. Anti-Müllerian hormone (AMH) is a growth factor secreted by growing follicles. Low levels of AMH correlate with longer cycle length (Harris et al, 2021). Other studies have connected AMH or antral follicle count (AFC), another ovarian reserve marker, to shorter cycles (Younis et al, 2020). A systematic review and meta-analysis have revealed in regularly cycling women, a shorter cycle is associated with lower ovarian reserve based on AMH or AFC (Younis et al, 2020). Young women diagnosed with premature ovarian failure have also reported shorter cycles (Guzel et al, 2017). In addition, it is well established in humans that diminishing ovarian reserve leads to perimenopause (a period or irregular cycles) and eventually menopause (complete cessation of cycles).

Empirical Evidence

Stressors that are known to deplete the ovarian reserve include cancer treatments, which kill primordial follicles and disrupt folliculogenesis. Alkylating chemotherapy agents like cyclophosphamide and cisplatin are highly ovotoxic, as well as radiation therapy towards ovaries (Pampanini et al, 2020). Therapies based on high dose alkylators and radiation towards ovaries lead with high likelihood to amenorrhea, infertility and premature ovarian insufficiency in humans due to depletion of ovarian reserve and therefore constitute an indication for clinical fertility preservation (ESHRE_Guideline_Group_on_Female_Fertility_Preservation et al, 2020; Pampanini et al, 2020).

In mice, it has been shown that cisplatin induces primordial follicle loss in a dose-depended manner(Meirow et al, 1999). Cyclophosphamide can accelerate primordial follicle loss in mice and in human ovarian tissue(Kalich-Philosoph et al., 2013; Lande et al., 2017). In human xenografts in mice, the same compound decreased the primordial follicle density(Oktem & Oktay, 2007). These key studies indicate the effect of chemotherapy compounds on the size of the ovarian reserve (Meirow et al, 2010). In treated patients, although there is often no direct information on the size of

the ovarian reserve, a rapid decrease of AMH levels has been observed following high risk chemotherapy. Patients receiving chemotherapy have also been shown to have an increased risk of premature ovarian failure. In addition, some studies have shown that the number of healthy follicles is significantly decreased and that of atretic follicles increased in human ovarian tissue following alkylating chemotherapy (Pampanini et al, 2019). These data establish a clear indication of diminished ovarian reserve following chemotherapy. Importantly, chemotherapy compounds also affect the menstrual cycle, with patients experiencing amenorrhea and irregular cycles (Jacobson et al., 2016; Meirow et al., 2010).

Another stressor known to affect the ovarian reserve is smoking. Cigarette smoke contains thousands of chemicals, several of which have been shown to be ovotoxic (Budani & Tiboni, 2017). Mice exposed in vivo to cigarette smoke have significantly fewer primordial follicles compared to the control group (Tuttle et al., 2009). Smoking women display reduced ovarian reserve markers and experience irregular cycles compared to non-smokers of the same age group (El-Nemr et al, 1998; Sharara et al, 1994).

Additional chemical insults affecting the ovarian reserve and causing menstrual cycle irregularities are presented in Table 1. These studies in animal models demonstrate how these chemicals directly target primordial follicles and cause menstrual cycle irregularities. Vinylcyclohexene diepoxide (VCD), metabolite of 4-vinylcyclohexene (VCH), is the most commonly used chemical in these studies and is often used to induce reproductive senescence in model organisms.

Table 1: *In vivo* studies demonstrating that effects on the KE upstream affect the KE downstream. VCD: vinylcyclohexene diepoxide, VCH: 4-vinylcyclohexene, BPA: bisphenol A, DEHP: bis(2-ethylhexyl) phthalate, B[a]P: Benzo[a]pyrene

Species	Compound	Dose	Duration	Reduced Ovarian Reserve	Ovarian Cycle Irregularities	Reference
Mouse	VCD	160 mg/kg/day	15 days	No primordial follicles at the end of exposure	All treated animals exhibited irregular cycles by day 58	(Mayer et al, 2004)
Mouse	VCD	160 mg/kg/day	15 days	No follicles on day 156	Longer cycles after day 28	(Lohff et al, 2005)
Rat	VCD	80 mg/kg/day	30 days	Reduced number of primordial and primary follicles at the end of exposure	Longer cycles by day 360	(Mayer et al, 2002)
Mouse	VCD	160 mg/kg/day	10 days 20 days	Reduced number of primordial and primary follicles by the end of 10-day exposure, all follicles reduced by the end of the 20-day exposure	Longer cycles on 135.1 ± 6.9 days for 10-day exposure and on 52.0 ± 2.2 days<br><br>for 20-day exposure	(Lohff et al, 2006)
Rat	VCD	80 mg/kg/day	30 days	Reduced number of primordial and primary follicles at the end of exposure	Irregular cycles of adult but not immature rats within the 30-day exposure	(Flaws et al, 1994)

Mouse	VCH	800 mg/kg/day	30 days	Reduced number of all follicles on day 30 – primordial and primary more affected, no follicles at day 360	Acyclicity by day 360	(Hooser et al, 1994)
Mouse	BPA	10, 100 µg/kg/day<br>1, 10 mg/kg/day	28 days	Reduced number of primordial follicles at the end of exposure	Longer cycles	(Hu et al, 2018)
Mouse	DEHP	20, 200 mg/kg/day	10 days	Reduced number of primordial follicles at the end of exposure	Longer cycles within the 10-day exposure	(Hannon et al, 2014)
Rat	B[a]P, DEHP,<br>B[a]P + DEHP	10 mg/kg/day B[a]P<br>600 mg/kg/day DEHP	60 days (on alternate days)	Reduced number of primordial follicles at the end of exposure	Longer cycles within the 60-day exposure	(Xu et al, 2010)

Uncertainties and Inconsistencies

As mentioned, several chemotherapy agents damage ovarian reserve and disrupt folliculogenesis. However, it has been shown that regular menses can resume upon treatment cessation (Jacobson et al, 2016). Therefore, in this case reduced ovarian reserve did not lead to permanent irregularities of ovarian cycle. In a systematic review and meta-analysis investigating the connection between the ovarian reserve and the length of the menstrual cycle, studies are mentioned where reduced ovarian reserve markers did not associate with irregular menstrual cycles (Younis et al, 2020). Several factors affect the impact of chemotherapy on ovarian health in humans, including the age at the treatment, size of ovarian reserve at treatment, and treatment regimen. However, late side effects of chemotherapy often include amenorrhea, premature ovarian insufficiency, and infertility.

Menstrual irregularities can be caused by factors other than reduced ovarian reserve. The most common factor affecting cyclicity is HPO axis dysregulation causing hypothalamic amenorrhea (Berga & Naftolin, 2012). Another example is the contraceptive pill that decreases gonadotropin secretion by the pituitary gland, leading to inhibition of folliculogenesis and amenorrhea. Changes in hormone levels produced by the pituitary gland have also been connected to shorter and anovulatory cycles (Mumford et al., 2012). Another factor affecting cyclicity is the thyroid gland function. Thyroid function disturbances, like hypo and hyperthyroidism have been connected to menstrual disturbances (Koutras, 1997).

Quantitative Understanding of the Linkage

poor

Time-scale

The timescale at which disruption in cyclicity occurs depends on the type of follicles that are affected, size of the reserve at the time of insult, and the extent of the damage. When a stressor targets selectively the ovarian reserve, it might take months (or years in humans) for the disruptions in cyclicity to be observed (Hoyer & Sipes, 1996). This delay was evident in some of the animal studies mentioned in Table 1 (Hooser et al., 1994; Lohff et al., 2006; Mayer et al., 2002).

Known modulating factors

The size of the ovarian reserve at the time of stressor exposure is a factor that can affect the response-response relationship of this KER. Therefore, age can also be a modulating factor, as observed in the animal study mentioned in table 1, where even though all treated rats exhibited reduction in the ovarian reserve, irregular cycles were only observed in the adult ones but not the immature ones (Flaws et al., 1994). In addition, chemotherapy effects on fertility tend to be more severe with increasing age due to a smaller ovarian reserve (Jacobson et al, 2016).

Changes in hormones can affect menstrual/estrus cyclicity, without being connected to the size of the ovarian reserve. For instance, experiencing stress has been shown to affect the hypothalamus-pituitary-adrenal axis (HPA) activity. A high body mass index (BMI) has been shown to affect sex hormone-binding globulin (SHBG), free androgen index (FAI), testosterone, and insulin levels. Smoking, although it can also affect the reserve, can cause hypoestrogenism. Therefore, stress, obesity and smoking can affect menstrual cyclicity and influence the response-response relationship of this KER (Bae et al, 2018).

Known Feedforward/Feedback loops influencing this KER

HPO axis regulates estrus/menstrual cycle, and is based on positive and negative feedback loops by ovarian steroids and peptide hormones, and hormones released by the hypothalamus and pituitary gland.

References

Bae J, Park SU, Kwon JW (2018) Factors associated with menstrual cycle irregularity and menopause. *BMC Womens Health* **18:** 36

Berga S, Naftolin F (2012) Neuroendocrine control of ovulation. *Gynecol Endocrinol* **28 Suppl 1:** 9-13

Budani MC, Tiboni GM (2017) Ovotoxicity of cigarette smoke: A systematic review of the literature. *Reprod Toxicol* **72:** 164-181

El-Nemr A, Al-Shawaf T, Sabatini L, Wilson C, Lower AM, Grudzinskas JG (1998) Effect of smoking on ovarian reserve and ovarian stimulation in in-vitro fertilization and embryo transfer. *Hum Reprod* **13:** 2192-2198

ESHRE_Guideline_Group_on_Female_Fertility_Preservation, Anderson RA, Amant F, Braat D, D'Angelo A, de Sousa Lopes SMC, Demeestere I, Dwek S, Frith L, Lambertini M, Maslin C, Moura-Ramos M, Nogueira D, Rodriguez-Wallberg K, Vermeulen N (2020) ESHRE guideline: female fertility preservation *Hum Reprod Update* **2020:** hoaa052

Flaws JA, Doerr JK, Sipes IG, Hoyer PB (1994) Destruction of preantral follicles in adult rats by 4-vinyl-1-cyclohexene diepoxide. *Reprod Toxicol* **8:** 509-514

Guzel Y, Aba YA, Yakin K, Oktem O (2017) Menstrual cycle characteristics of young females with occult primary ovarian insufficiency at initial diagnosis and one-year follow-up with serum amh level and antral follicle count. *PLoS One* **12:** e0188334

Hannon PR, Peretz J, Flaws JA (2014) Daily exposure to Di(2-ethylhexyl) phthalate alters estrous cyclicity and accelerates primordial follicle recruitment potentially via dysregulation of the phosphatidylinositol 3-kinase signaling pathway in adult mice. *Biol Reprod* **90:** 136

Harris BS, Steiner AZ, Jukic AM (2021) Ovarian Reserve Biomarkers and Menstrual Cycle Length in a Prospective Cohort Study. *J Clin Endocrinol Metab* **106:** e3748-e3759

Hooser SB, Douds DP, DeMerell DG, Hoyer PB, Sipes IG (1994) Long-term ovarian and gonadotropin changes in mice exposed to 4-vinylcyclohexene. *Reprod Toxicol* **8:** 315-323

Hoyer PB, Sipes IG (1996) Assessment of follicle destruction in chemical-induced ovarian toxicity. *Annu Rev Pharmacol Toxicol* **36:** 307-331

Hu Y, Yuan DZ, Wu Y, Yu LL, Xu LZ, Yue LM, Liu L, Xu WM, Qiao XY, Zeng RJ, Yang ZL, Yin WY, Ma YX, Nie Y (2018) Bisphenol A Initiates Excessive Premature Activation of Primordial Follicles in Mouse Ovaries via the PTEN Signaling Pathway. *Reprod Sci* **25:** 609-620

Jacobson MH, Mertens AC, Spencer JB, Manatunga AK, Howards PP (2016) Menses resumption after cancer treatment-induced amenorrhea occurs early or not at all. *Fertil Steril* **105:** 765-772

Kalich-Philosoph, L., Roness, H., Carmely, A., Fishel-Bartal, M., Ligumsky, H., Paglin, S., Wolf, I., Kanety, H., Sredni, B., & Meirow, D. (2013). Cyclophosphamide triggers follicle activation and "burnout "; AS101 prevents follicle loss and preserves fertility. *Science Translational Medicine*, *5*(185).

Koutras, D. A. (1997). Disturbances of menstruation in thyroid disease. *Annals of the New York Academy of Sciences*, *816*, 280–284.

Lande, Y., Fisch, B., Tsur, A., Farhi, J., Prag-Rosenberg, R., Ben-Haroush, A., Kessler-Icekson, G., Zahalka, M. A., Ludeman, S. M., & Abir, R. (2017). Short-term exposure of human ovarian follicles to cyclophosphamide metabolites seems to promote follicular activation in vitro. *Reproductive BioMedicine Online*, *34*(1), 104–114.

Lohff JC, Christian PJ, Marion SL, Arrandale A, Hoyer PB (2005) Characterization of cyclicity and hormonal profile with impending ovarian failure in a novel chemical-induced mouse model of perimenopause. *Comp Med* **55:** 523-527

Lohff JC, Christian PJ, Marion SL, Hoyer PB (2006) Effect of duration of dosing on onset of ovarian failure in a chemical-induced mouse model of perimenopause. *Menopause* **13:** 482-488

Mayer LP, Devine PJ, Dyer CA, Hoyer PB (2004) The follicle-deplete mouse ovary produces androgen. *Biol Reprod* **71:** 130-138

Mayer LP, Pearsall NA, Christian PJ, Devine PJ, Payne CM, McCuskey MK, Marion SL, Sipes IG, Hoyer PB (2002) Long-term effects of ovarian follicular depletion in rats by 4-vinylcyclohexene diepoxide. *Reprod Toxicol* **16:** 775-781

Meirow D, Biederman H, Anderson RA, Wallace WHB (2010) Toxicity of chemotherapy and radiation on female reproduction. *Clin Obstet Gynecol* **53:** 727-739

Meirow D, Lewis H, Nugent D, Epstein M (1999) Subclinical depletion of primordial follicular reserve in mice treated with cyclophosphamide: clinical importance and proposed accurate investigative tool. *Hum Reprod* **14:** 1903-1907

Mumford, S. L., Steiner, A. Z., Pollack, A. Z., Perkins, N. J., Filiberto, A. C., Albert, P. S., Mattison, D. R., Wactawski-Wende, J., & Schisterman, E. F. (2012). The utility of menstrual cycle length as an indicator of cumulative hormonal exposure. *Journal of Clinical Endocrinology and Metabolism*, *97*(10).

O'Connor KA, Holman DJ, Wood JW (1998) Declining fecundity and ovarian ageing in natural fertility populations. *Maturitas* **30:** 127-136

O'Connor KA, Holman DJ, Wood JW (2001) Menstrual cycle variability and the perimenopause. *Am J Hum Biol* **13:** 465-478

OECD. (2001) Test No. 416: Two-Generation Reproduction Toxicity, OECD Guidelines for the Testing of Chemicals, Section 4. OECD Publishing, Paris.

OECD. (2008) Test No. 407: Repeated Dose 28-day Oral Toxicity Study in Rodents. OECD Publishing, Paris.

OECD. (2018) Test No. 443: Extended One-Generation Reproductive Toxicity Study, OECD Guidelines for the Testing of Chemicals, Section 4. OECD Publishing, Paris.

Oktem O, Oktay K (2007) A novel ovarian xenografting model to characterize the impact of chemotherapy agents on human primordial follicle reserve. *Cancer Res* **67:** 10159-10162

Pampanini V, Hassan J, Oliver E, Stukenborg JB, Damdimopoulou P, Jahnukainen K (2020) Fertility Preservation for Prepubertal Patients at Risk of Infertility: Present Status and Future Perspectives. *Horm Res Paediatr* **93:** 599-608

Pampanini V, Wagner M, Asadi-Azarbaijani B, Oskam IC, Sheikhi M, Sjödin MOD, Lindberg J, Hovatta O, Sahlin L, Björvang RD, Otala M, Damdimopoulou P, Jahnukainen K (2019) Impact of first-line cancer treatment on the follicle quality in cryopreserved ovarian samples from girls and young women. *Hum Reprod* **34:** 1674-1685

Sharara FI, Beatse SN, Leonardi MR, Navot D, Scott Jr RT (1994) Cigarette smoking accelerates the development of diminished ovarian reserve as evidenced by the clomiphene citrate challenge test. *Fertil Steril* **62:** 257-262

Tuttle AM, Stämpfli M, Foster WG (2009) Cigarette smoke causes follicle loss in mice ovaries at concentrations representative of human exposure. *Hum Reprod* **24:** 1452-1459

Wallace WHB, Kelsey TW (2010) Human ovarian reserve from conception to the menopause. *PLoS One* **5:** e8772

Windham GC, Elkin EP, Swan SH, Waller KO, Fenster L (1999) Cigarette smoking and effects on menstrual function. *Obstet Gynecol* **93:** 59-65

Xu C, Chen JA, Qiu Z, Zhao Q, Luo J, Yang L, Zeng H, Huang Y, Zhang L, Cao J, Shu W (2010) Ovotoxicity and PPAR-mediated aromatase downregulation in female Sprague-Dawley rats following combined oral exposure to benzo[a]pyrene and di-(2-ethylhexyl) phthalate. *Toxicol Lett* **199:** 323-332

Younis JS, Iskander R, Fauser BMJM, Izhaki I (2020) Does an association exist between menstrual cycle length within the normal range and ovarian reserve biomarkers during the reproductive years? A systematic review and meta-analysis. *Hum Reprod Update* **26:** 904-928

Relationship: 394: disrupted, ovarian cycle leads to decreased, Fertility

AOPs Referencing Relationship

AOP Name	Adjacency	Weight of Evidence	Quantitative Understanding
Aromatase (Cyp19a1) reduction leading to impaired fertility in adult female	non-adjacent	Moderate	
Decreased ALDH1A (RALDH) activity leading to decreased fertility via disrupted meiotic initiation of fetal oogonia	adjacent	High	Low

AOP Name	Adjacency	Weight of Evidence	Quantitative Understanding
Androgen receptor (AR) antagonism leading to decreased fertility in females	adjacent	High	Low

Evidence Supporting Applicability of this Relationship

Taxonomic Applicability

Term	Scientific Term	Evidence	Links
human, mouse, rat	human, mouse, rat	High	NCBI

Life Stage Applicability

Life Stage	Evidence
Adult, reproductively mature	High

Sex Applicability

Sex	Evidence
Female	High

In many instances, human female reproductive toxicity of an agent is suspected based on studies performed in experimental animals. The neuroendocrinology, steroid biochemistry, and other physiologic events in the females of most small experimental species often used (mouse, rat, hamster) are similar in their susceptibility to disruption by toxicants (Massaro, 1997).

Although the assessment of the human ovarian cycle may have a variety of biomarkers distinct from those in rats, many of the underlying endocrine mechanisms associated with successful follicular development, ovulation, pregnancy, and parturition are homologous between the two (for review see (Bretveld et al., 2006). For this reason, a toxicant-induced perturbation of ovarian cycles in female rats suggest that a compound may function as a reproductive toxicant in human females.

Mice

- environmental air pollution (Mohallem et al., 2005)
- phthalates (DEHP)
- abortion rate of 100% in F0 dams in the 500-mg/kg/day was observed, in F1 females found that the total number of F2 embryos (exposed to DEHP only as germ cells) was not impaired. However, in the 0.05- and 5-mg DEHP groups, 28% and 29%, respectively, of the blastocysts were degenerated, compared with 8% of controls (Schmidt et al., 2012).
- Lamb et al. studied fertility effects of DEHP in mice (both sexes) and found that DEHP caused dose-dependent decreases in fertility. DBP exposure resulted in a reduction in the numbers of litters per pair and of live pups per litter and in the proportion of pups born alive at the 1.0% amount, but not at lower dose levels. A crossover mating trial demonstrated that female mice, but not males, were affected by DBP, as shown by significant decreases in the percentage of fertile pairs, the number of live pups per litter, the proportion of pups born alive, and live pup weight. DHP in the diet resulted in dose-related adverse effects on the numbers of litters per pair and of live pups per litter and proportion of pups born alive at 0.3, 0.6, and 1.2% DHP in the diet. A crossover mating study demonstrated that both sexes were affected. DEHP (at 0.1 and 0.3%) caused dose-dependent decreases in fertility and in the number and the proportion of pups born alive. A crossover mating trial showed that both sexes were affected by exposure to DEHP. These data demonstrate the ability of the continuous breeding protocol to discriminate the qualitative and quantitative reproductive effects of the more and less active congeners as well as the large differences in reproductive toxicity attributable to subtle changes in the alkyl substitution of phthalate esters (Lamb et al., 1987).

Rat phthalates (DEHP)

- female rats exposed to a high dose of DEHP (3,000 mg/kg/day) had irregular estrous cycles and a slight decline in pregnancy rate (Takai et al., 2009). At 1,000 mg/kg bw/day over a period of 4 weeks did not disturb female fertility or early embryo development.

- There was significant evidence that 5, 15, 50, and 400 mg /kg/day females differed from the control females in the relative amount of time spent in oestrous stages, however no changes were revealed in the number of females with regular cycles, cycle length, number of cycles, and in number of cycling females across the dose groups as compared to the control females The litter size (number of live pups) produced by the P0 generation was significantly reduced in the 400 mg/kg/day dose group (Blystone et al., 2010).

Human

Studies showing a correlation between decreased fertility and;

- professional activity (Olsen, 1994)
- phthalates (DEHP) In occupationally exposed women to high concentration of phthalates exhibit hypoestrogenic anovulary cycles and was associated with decreased pregnancy rate and higher miscarriage rates (Aldyreva,M.V.,Klimove,T.S.,Iziumova,A.S.,Timofeevskaia,L.A., 1975).
- smoking (Hull, North, Taylor, Farrow, & Ford, 2000)
- the use of certain drugs or radiation exposure (Dobson & Felton, 1983)

For the taxonomic applicability see also the Table 1.

Key Event Relationship Description

The ovarian cycle irregularities impact on reproductive capacity of the females that may result in impaired fertility:

1. Irregular cycles may reflect impaired ovulation. Extended vaginal estrus usually indicates that the female cannot spontaneously achieve the ovulatory surge of LH (Huang and Meites, 1975). The persistence of regular vaginal cycles after treatment does not necessarily indicate that ovulation occurred, because luteal tissue may form in follicles that have not ruptured. However, that effect should be reflected in reduced fertility. Conversely, subtle alterations of cyclicity can occur at doses below those that alter fertility (Gray et al., 1989).

2. Persistent or constant vaginal cornification (or vaginal estrus) may result from one or several effects. Typically, in the adult, if the vaginal epithelium becomes cornified and remains so in response to toxicant exposure, it is the result of the agent's estrogenic properties (i.e., DES or methoxychlor), or the ability of the agent to block ovulation. In the latter case, the follicle persists and endogenous estrogen levels bring about the persistent vaginal cornification. Histologically, the ovaries in persistent estrus will be atrophied following exposure to estrogenic substances. In contrast, the ovaries of females in which ovulation has been blocked because of altered gonadotropin secretion will contain several large follicles and no corpora lutea. Females in constant estrus may be sexually receptive regardless of the mechanism responsible for this altered ovarian condition. However, if ovulation has been blocked by the treatment, an LH surge may be induced by mating (Brown-Grant et al., 1973; Smith, E.R. and Davidson, 1974) and a pregnancy or pseudopregnancy may ensue. The fertility of such matings is reduced (Cooper et al., 1994).

3. Significant delays in ovulation can result in increased embryonic abnormalities and pregnancy loss (Fugo and Butcher, 1966; Cooper et al., 1994).

4. Persistent diestrus indicates temporary or permanent cessation of follicular development and ovulation, and thus at least temporary infertility.

5. Prolonged vaginal diestrus, or anestrus, may be indicative of agents (e.g., polyaromatic hydrocarbons) that interfere with follicular development or deplete the pool of primordial follicles (Mattison and Nightingale, 1980) or agents such as atrazine that interrupt gonadotropin support of the ovary (Cooper et al., 1996). Pseudopregnancy is another altered endocrine state reflected by persistent diestrus. The ovaries of anestrous females are atrophic, with few primary follicles and an unstimulated uterus (Huang and Meites, 1975). Serum estradiol and progesterone are abnormally low.

6. Lengthening of the cycle may be a result of increased duration of either estrus or diestrus.

Evidence Supporting this KER

Biological Plausibility

In females, normal reproductive function involves the appropriate interaction of central nervous system, anterior pituitary, oviducts, uterus, cervix and ovaries. During the reproductive years the ovary is the central organ in this axis. The functional unit within the ovary is the follicle which is composed of theca; granulosa cells and the oocyte. The somatic compartment synthesizes and secrets hormones (steroids and growth factors) necessary for the orchestration of the inter-relationship between the other parts of the reproductive tract and the central nervous system. Oestrus cycle is under strict hormonal control, therefore perturbations of hormonal balance lead to perturbations of normal cyclicity (change in number of cycles or duration of each phase) and/or ovulation problems leading to impaired female reproductive function. However, there are other mechanisms that might result in impaired fertility (e.g cellular maturation in ovary).

Empirical Evidence

Many chemicals are found to interfere with reproductive function in the female. This interference is commonly expressed as a change in normal morphology of the reproductive tract or in ovarian cycle irregularities (disturbance in the duration of particular phases of the estrous cycle and/or ovulation problems). Monitoring estrous cyclicity provides a means to identify alterations in reproductive functions which are mediated through nonestrogenic as well as estrogenic mechanisms (Blasberg, Langan, & Clark, 1997), (Clark, Blasberg, & Brandling-Bennett, 1998). Adverse alteration in the nonpregnant female reproductive system have been observed at dose levels below those that result in reduced fertility or produce other overt effects on pregnancy or pregnancy outcomes. A disruption of cycling caused by xenobiotic treatment can induce a persistent estrus, a persistent diestrus, an irregular pattern with cycles of extended duration and ovulation problems. Common classes of chemicals have been shown to cause cycle irregularities in rats, humans, and non-human primates. Examples include the polychlorinated biphenyls (PCBs) and

dioxins, which are associated with such irregularities in rats and humans (e.g (Li, Johnson, & Rozman, 1995) (Meerts et al., 2004), (Chao, Wang, Lin, Lee, & Päpke, 2007) and various agricultural pesticides, including herbicides, fungicides, and fumigants for review see (Bhattacharya & Keating, 2012),(Bretveld, Thomas, Scheepers, Zielhuis, & Roeleveld, 2006).

Compound class	**Species**	**AO:ovarian cycle irregularities**	**AO:Impaired fertility**	reference
Phthalates (DEHP)	rat	5-400 mg/kg/day females differed from the control in the relative amount of time spent in oestrous stages	number of live pups (P0) reduced (400 mg/kg/day)	(Blystone et al., 2010)
Phthalates (DEHP)	rat	irregular estrous cycles (3,000 mg/kg/day)	slight decline in pregnancy rate (3,000 mg/kg/day)	(Takai et al., 2009)
Phthalates (DEHP)	mice		dose-dependent decreases in fertility	(Lamb, Chapin, Teague, Lawton, & Reel, 1987)
Phthalates (DEHP)	mice	No change	abortion rate of 100% in F0 dams (500-mg/kg/day)	(Schmidt, Schaedlich, Fiandanese, Pocar, & Fischer, 2012).
Phthalates (DEHP)	sheep	dose-dependent effect on the duration of the estrous cycles shortening of the ovulatory cycles due mainly to a reduction in the size and lifespan of CL		(Herreros, Gonzalez-Bulnes, et al., 2013)
Phthalates (DEHP)	sheep	No effect on ovulatory efficiency		(Herreros, Encinas, et al., 2013)
Phthalates (DEHP)	rat	No changes in F0, increase of cycle by 0.4 day in F1 at 10,000ppm	18% and 21% decrease in live pups/litter F0 at 7500ppm and 10,000ppm respectively, no viable litters (F1 10,000 ppm ~643.95mg/kg/day)	(NTP, 2005)
Phthalates (DEHP)	rat	Deficit in growing follicles and corpora lutea	4-fold increase in females with stillborn pups in F0 at 9000ppm 2.1-fold Postimplantation loss in F0 at 9000ppm	(Schilling, K., Deckardt. K., Gembardt, Chr., and Hildebrand, 1999)
Phthalates (DEHP)	rat	prolong the estrous cycle, anovulation		(Davis, Maronpot, & Heindel, 1994)
Phthalates			Reduced fertility and fecundity	(Wolf et al., 1999)
Organochlorine (methoxychlor)	rat	Decreased number of cycles, extended diestrus and estrus		(Laws, 2000)
Organotins tributyltin chloride (TBTCl)	rat	At 125 ppm vaginal opening and impaired estrous cyclicity		(Ogata et al., 2001)

Table 1 Summary the empirical evidence supporting the KER.

It is known that exposure to 17-β-estradiol can disrupt the normal 4- to 5-day estrous cycle in adult female rats by inducing an extended period of diestrus consistent with pseudopregnancy within 5–7 days after the exposure (Gilmore & McDonald, 1969). This is due to the estrogen-dependent increase in prolactin that rescues ovarian corpora lutea and the subsequent synthesis and release of progesterone (Cooper, R. L., and Goldman, 1999). Significant evidence that the estrous cycle (or menstrual cycle in primates) has been disrupted should be considered an adverse effect (OECD, 2008).

Uncertainties and Inconsistencies

Chemicals may be found to interfere with reproductive function in the female. This interference is commonly expressed as a change in normal morphology of the reproductive tract or a disturbance in the duration of particular phases of the estrous cycle. However, menstrual cyclicity is affected by many parameters such as age, nutritional status, stress, exercise level, certain drugs, and the use of contraceptive measures that alter endocrine feedback. In nonpregnant females, repetitive occurrence of the four stages of the estrous cycle at regular, normal intervals suggests that neuroendocrine control of the cycle and ovarian responses to that control are normal. Even normal, control animals can show irregular cycles. However, a significant alteration compared with controls in the interval between occurrence of estrus for a treatment group is cause for concern. Generally, the cycle will be lengthened or the animals will become acyclic. Therefore changes in cyclicity should be interpreted with caution and not judged adverse without a comprehensive consideration of additional relevant endpoints in a weight-of-evidence approach.

Inconsistencies

Two generation studies by Tyl et al with Butyl benzyl phthalate (BBP) did not observe effects in F0 females on any parameters of estrous cycling, mating, or gestation. However, F1 females carrying F2 litters at and reduced number of total and live pups/litter at birth, with no effects on pre- or postnatal survival (Tyl et al., 2004).

Quantitative Understanding of the Linkage

Response-response relationship

A systematic review and meta-analysis, has made a correlation between menstrual cycle length and outcomes including fecundity defined by antral follicle count or anti-mullerian hormone (AMH) levels (Younis et al., 2020). This study can be used to provide quantitative information for this KER for humans. A short menstrual cycle length (21-27 days) was correlated with lower AMH levels and antral follicle counts.

Time-scale

Ovarian cycle irregularities encompass disturbances in the ovarian cycle and/or ovulation issues. In cases of anovulation, the time scale is immediate, whereas other irregularities depend on the duration of folliculogenesis and the menstrual or estrous cycle specific to each species.

References

Aldyreva,M.V.,Klimove,T.S.,Iziumova,A.S.,Timofeevskaia,L.A. (1975). The effect of phthalate plasticizers on the generative function. Gig.Tr.Prof.Zabol., (19), 25–29.

Bhattacharya, P., & Keating, A. F. (2012). Impact of environmental exposures on ovarian function and role of xenobiotic metabolism during ovotoxicity. Toxicology and Applied Pharmacology, 261(3), 227–35. doi:10.1016/j.taap.2012.04.009

Blasberg, M. E., Langan, C. J., & Clark, A. S. (1997). The effects of 17 alpha-methyltestosterone, methandrostenolone, and nandrolone decanoate on the rat estrous cycle. Physiology & Behavior, 61(2), 265–72.

Blystone, C. R., Kissling, G. E., Bishop, J. B., Chapin, R. E., Wolfe, G. W., & Foster, P. M. D. (2010). Determination of the di-(2-ethylhexyl) phthalate NOAEL for reproductive development in the rat: importance of the retention of extra animals to adulthood. Toxicological Sciences : An Official Journal of the Society of Toxicology, 116(2), 640–6. doi:10.1093/toxsci/kfq147

Bretveld, R. W., Thomas, C. M. G., Scheepers, P. T. J., Zielhuis, G. A., & Roeleveld, N. (2006). Pesticide exposure: the hormonal function of the female reproductive system disrupted? Reproductive Biology and Endocrinology : RB&E, 4(1), 30. doi:10.1186/1477-7827-4-30

Chao, H.-R., Wang, S.-L., Lin, L.-Y., Lee, W.-J., & Päpke, O. (2007). Placental transfer of polychlorinated dibenzo-p-dioxins, dibenzofurans, and biphenyls in Taiwanese mothers in relation to menstrual cycle characteristics. Food and Chemical Toxicology : An International Journal Published for the British Industrial Biological Research Association, 45(2), 259–65. doi:10.1016/j.fct.2006.07.032

Clark, A. S., Blasberg, M. E., & Brandling-Bennett, E. M. (1998). Stanozolol, oxymetholone, and testosterone cypionate effects on the rat estrous cycle. Physiology & Behavior, 63(2), 287–95.

Cooper, R. L., and Goldman, J. M. (1999). Vaginal cytology. In An Evaluation and Interpretation of Reproductive Endpoints for Human Health Risk Assessment. Washington. Davis, B. J., Maronpot, R. R., & Heindel, J. J. (1994). Di-(2-ethylhexyl) phthalate suppresses estradiol and ovulation in cycling rats. Toxicology and Applied Pharmacology, 128(2), 216–23. doi:10.1006/taap.1994.1200

Dobson, R. L., & Felton, J. S. (1983). Female germ cell loss from radiation and chemical exposures. American Journal of Industrial Medicine, 4(1-2), 175–90.

Gilmore, D. P., & McDonald, P. G. (1969). Induction of prolonged diestrus in the rat by a low level of estrogen. Endocrinology, 85(5), 946–8. doi:10.1210/endo-85-5-946 Herreros, M. A., Encinas, T., Torres-Rovira, L., Garcia-Fernandez, R. A., Flores, J. M., Ros, J. M., & Gonzalez-Bulnes, A. (2013). Exposure to the endocrine disruptor di(2-ethylhexyl)phthalate affects female reproductive features by altering pulsatile LH secretion. Environmental Toxicology and Pharmacology, 36(3), 1141–9. doi:10.1016/j.etap.2013.09.020

Herreros, M. A., Gonzalez-Bulnes, A., Iñigo-Nuñez, S., Contreras-Solis, I., Ros, J. M., & Encinas, T. (2013). Toxicokinetics of di(2-ethylhexyl) phthalate (DEHP) and its effects on luteal function in sheep. Reproductive Biology, 13(1), 66–74. doi:10.1016/j.repbio.2013.01.177

Hull, M. G., North, K., Taylor, H., Farrow, A., & Ford, W. C. (2000). Delayed conception and active and passive smoking. The Avon Longitudinal Study of Pregnancy and Childhood Study Team. Fertility and Sterility, 74(4), 725–33.

Lamb, J. C., Chapin, R. E., Teague, J., Lawton, A. D., & Reel, J. R. (1987). Reproductive effects of four phthalic acid esters in the mouse. Toxicology and Applied Pharmacology, 88(2), 255–69.

Laws, S. C. (2000). Estrogenic Activity of Octylphenol, Nonylphenol, Bisphenol A and Methoxychlor in Rats. Toxicological Sciences, 54(1), 154–167. doi:10.1093/toxsci/54.1.154

Li, X., Johnson, D. C., & Rozman, K. K. (1995). Effects of 2,3,7,8-tetrachlorodibenzo-p-dioxin (TCDD) on estrous cyclicity and ovulation in female Sprague-Dawley rats. Toxicology Letters, 78(3), 219–22.

Massaro, E. J. (Ed.). (1997). Handbook of Human Toxicology, Volume 236. Taylor & Francis.

Meerts, I. A. T. M., Hoving, S., van den Berg, J. H. J., Weijers, B. M., Swarts, H. J., van der Beek, E. M., ... Brouwer, A.

(2004). Effects of in utero exposure to 4-hydroxy-2,3,3’,4',5-pentachlorobiphenyl (4-OH-CB107) on developmental landmarks, steroid hormone levels, and female estrous cyclicity in rats. Toxicological Sciences : An Official Journal of the Society of Toxicology, 82(1), 259–67. doi:10.1093/toxsci/kfh251

Mohallem, S. V., de Araújo Lobo, D. J., Pesquero, C. R., Assunção, J. V., de Andre, P. A., Saldiva, P. H. N., & Dolhnikoff, M. (2005). Decreased fertility in mice exposed to environmental air pollution in the city of Sao Paulo. Environmental Research, 98(2), 196–202. doi:10.1016/j.envres.2004.08.007

NTP. (2005). Multigenerational Reproductive Assessment by Continuous Breeding when Diethylhexylphthalate (CAS 117-81-7).

OECD. (2008). No 43: Guidance document on mammalian reproductive toxicity testing and assessment.

Ogata, R., Omura, M., Shimasaki, Y., Kubo, K., Oshima, Y., Aou, S., & Inoue, N. (2001). Two-generation reproductive toxicity study of tributyltin chloride in female rats. Journal of Toxicology and Environmental Health. Part A, 63(2), 127–44. doi:10.1080/15287390151126469

Olsen, J. (1994). Is human fecundity declining--and does occupational exposures play a role in such a decline if it exists? Scandinavian Journal of Work, Environment & Health, 20 Spec No, 72–7.

Schilling, K., Deckardt. K., Gembardt, Chr., and Hildebrand, B. (1999). Di-2-ethylhexyl phthalate – two-generation reproduction toxicity range-finding study in Wistar rats. Continuos dietary administration.

Schmidt, J.-S., Schaedlich, K., Fiandanese, N., Pocar, P., & Fischer, B. (2012). Effects of di(2-ethylhexyl) phthalate (DEHP) on female fertility and adipogenesis in C3H/N mice. Environmental Health Perspectives, 120(8), 1123–9. doi:10.1289/ehp.1104016

Takai, R., Hayashi, S., Kiyokawa, J., Iwata, Y., Matsuo, S., Suzuki, M., … Deki, T. (2009). Collaborative work on evaluation of ovarian toxicity. 10) Two- or four-week repeated dose studies and fertility study of di-(2-ethylhexyl) phthalate (DEHP) in female rats. The Journal of Toxicological Sciences, 34 Suppl 1(I), SP111–9.

Tyl, R. W., Myers, C. B., Marr, M. C., Fail, P. a, Seely, J. C., Brine, D. R., … Butala, J. H. (2004). Reproductive toxicity evaluation of dietary butyl benzyl phthalate (BBP) in rats. Reproductive Toxicology (Elmsford, N.Y.), 18(2), 241–64. doi:10.1016/j.reprotox.2003.10.006

Wolf, C., Lambright, C., Mann, P., Price, M., Cooper, R. L., Ostby, J., & Gray, L. E. (1999). Administration of potentially antiandrogenic pesticides (procymidone, linuron, iprodione, chlozolinate, p,p’-DDE, and ketoconazole) and toxic substances (dibutyl- and diethylhexyl phthalate, PCB 169, and ethane dimethane sulphonate) during sexual differen. Toxicology and Industrial Health, 15(1-2), 94–118. doi:10.1177/074823379901500109

Younis, J. S., Iskander, R., Fauser, B. C. J. M., & Izhaki, I. (2020). Does an association exist between menstrual cycle length within the normal range and ovarian reserve biomarkers during the reproductive years? A systematic review and meta-analysis. *Human Reproduction Update*, *26*(6), 904–928. https://doi.org/10.1093/humupd/dmaa013
