# Supplementary material for: AOP Report: Decreased ALDH1A (RALDH) activity leading to decreased fertility via disrupted meiotic initiation of fetal oogonia
Source: Curr Res Toxicol. 2025 Sep 4;9:100257. doi: 10.1016/j.crtox.2025.100257 (PMC12547181; doi:10.1016/j.crtox.2025.100257)
Supplement: Supplementary Data 2 [file mmc2.pdf]

Essentiality table (as suggested in handbook table 5)

Event	Direct evidence	Uncertainties, inconsistencies, and contradictory evidence
**MIE 1880**<br>Decreased, ALDH1A activity<br><br>LOW: There is direct experimental evidence from KO studies that ALDH1A is essential for atRA synthesis. Some studies on KO and inhibition of ALDH1A show an effect on initiation of meiosis in ovary but there are contradictory studies.	• KO of ALDH1A isoforms blocks atRA synthesis in vivo in mice (Niederreither et al, 1999, Dupé et al, 2003; Fan et al, 2003; Molotkov & Duester, 2003).<br>• KO of ALDH1A1 results in delayed germ cell meiosis in mouse fetal embryos (Bowles et al., 2016).<br>• Inhibition of ALDH1A2 in mouse ovary culture results in failure to induce expression of meiotic marker Stra8 and subsequent germ cell loss (Rosario et al, 2020).<br>• Inhibition of ALDH1A in mouse ovary cultures blocked germ cell meiotic entry (Mu et al, 2013).<br>• Inhibition of ALDH1 partially inhibits meiotic entry in human fetal ovaries (Le Bouffant et al, 2010)	KO of ALDH1A2 and double knockout of ALDH1A2 and ALDH1A3 showed no reduction of Stra8 expression in fetal ovary and ALDH1A2 knockout did not prevent meiotic initiation (Kumar et al., 2012).<br>Triple KO of ALDH1A1-3 showed a reduced Stra8 expression in fetal ovary but the knockout did not prevent meiotic initiation (Chassot,et al, 2020).<br>These results indicate that atRA synthesis is not the only determinant for initiation of meiosis in oocytes as discussed in Spiller & Bowles, 2022 and Shimada and Ishiguro, 2023.
**KE1881**<br>Decreased atRA concentration<br><br>LOW: Some studies show that atRA is essential for initiation of meiosis in ovary but there are	• Oocytes fail to enter meiosis in ovaries of vitamin A deficient rats due to atRA deficiency (Li & Clagett-Dame, 2009)<br>• Inhibition of RAR in mouse embryonic ovary cultures results in failure to induce Stra8 expression (Bowles et al, 2006; Koubova et al, 2006; Minkina et al, 2017).<br>• atRA activates meiosis-related gene network in mouse embryonic stem cells (Aoki & Takada, 2012), increases meiosis resumption in mouse oocytes (Tahaei et al, 2011), promote germ cell meiotic initiation in cultured fetal human ovaries (Le Bouffant et al, 2010), mouse ovaries (Livera et al, 2000), and chicken ovaries (Yu et al, 2013) and camel oocytes (Saadeldin	Triple knockout of RAR-α, -β, -γ showed a reduced Stra8 expression in fetal ovary but the knockout did not prevent meiotic initiation (Vernet et al, 2020).<br><br>Mutation of two retinoic acid response elements (RAREs) in the Stra8 promoter in mice reduced Stra8 expression in fetal ovary but did not prevent meiotic initiation (Feng et al, 2021).<br><br>These results, together with the studies on ALDH1A KO, described above indicate that atRA is not the only determinant for initiation of meiosis in oocytes as discussed in Spiller & Bowles, 2022 and Shimada & Ishiguro, 2023.

contradictory studies.	et al, 2019). RAR agonist accelerates meiotic entry in mouse fetal oocytes (Livera et al, 2000).	
**KE1882** Disrupted, meiotic initiation in oocytes<br><br>HIGH: There is direct evidence from experimental studies that disruption of meiosis in ovary results in reduced fertility.	• In mice, ablation of Stra8 prevents oocytes from entering meiosis in the fetal ovaries and mature females are infertile (Baltus et al, 2006; Zhou et al, 2008).<br>• Mutation in Atm, a gene involved in recombination during meiosis, results in complete loss of primary oocytes in mice, and greatly reduced follicle pool in humans (Adelfalk et al, 2011; Agamanolis & Greenstein, 1979; Aguilar et al, 1968; Xu et al, 1996).<br>• Mutation to Fanca and Fancd2 genes that are involved in recombination lead to oocyte degeneration and subfertility in mice (Cheng et al, 2000; Houghtaling et al, 2003; Wong et al, 2003).<br>• Mice with Lhx8 ablation display total loss of oocytes. Lhx8-/- mice maintain oocytes during fetal development, but loose the oocytes shortly after birth by autophagy, likely because the oocytes have failed to enter meiosis in utero (Choi et al, 2008; D'Ignazio et al, 2018).	
**KE1883** Decreased ovarian reserve<br><br>MODERATE: There is indirect evidence that chemicals that reduce the ovarian reserve also affect the ovarian cycle.	INDIRECT EVIDENCE<br>• In mice and rats, a chemically induced reduced follicle pool results in irregular cycles in vivo (Mayer et al, 2004, Lohff et al, 2005, Lohff et al, 2006, Mayer et al, 2002, Flaws et al, 1994, Hooser et al, 1994, Hu et al, 2018, Hannon et al, 2014, Xu et al, 2010).<br>• In humans, chemotherapy can affect ovarian reserve as well as the menstrual cycle (Jacobson et al, 2016; Meirow et al., 2010). Smoking, that reduces primordial follicles in mice (Tuttle et al, 2009) is also associated with irregular cycles in humans (El-Nemr et al, 1998; Sharara et al, 1994).	**Contradictory evidence:**<br>Several chemotherapy agents damage ovarian reserve and disrupt folliculogenesis. However, it has been shown that regular menses can resume upon treatment cessation (Jacobson et al, 2016). Therefore, in this case reduced ovarian reserve did not lead to permanent irregularities of ovarian cycle. In a systematic review and meta-analysis investigating the connection between the ovarian reserve and the length of the menstrual cycle, studies are mentioned where reduced ovarian reserve markers did not associate with irregular menstrual cycles (Younis et al, 2020). Several factors affect the impact of chemotherapy on ovarian health in humans, including the age at the treatment, size of ovarian reserve at treatment, and treatment regimen. However, late side effects of chemotherapy often include amenorrhea, premature ovarian insufficiency, and infertility.
**KE405**	INDIRECT EVIDENCE	

Disrupted, ovarian cycle<br><br>MODERATE: There is indirect evidence that chemicals that affect the ovarian cycle also cause impaired fertility.	• In mice and rats, a chemically induced cycle irregularity is associated with impaired fertility *in vivo* (Blystone et al., 2010, Takai et al., 2009, NTP, 2005)	

References

Adelfalk C, Ahmed EA, Scherthan H. Reproductive Phenotypes of Mouse Models Illuminate Human Infertility. J Reproduktionsmed Endokrinol 2011; 8 (6): 376–83.

Agamanolis DP, Greenstein JI. Ataxia-telangiectasia. Report of a case with Lewy bodies and vascular abnormalities within cerebral tissue. J Neuropathol Exp Neurol. 1979 Sep;38(5):475-89. doi: 10.1097/00005072-197909000-00003

Aguilar MJ, Kamoshita S, Landing BH, Boder E, Sedgwick RP. Pathological observations in ataxia-telangiectasia. A report of five cases. J Neuropathol Exp Neurol. 1968 Oct;27(4):659-76

Aoki T, Takada T. Bisphenol A modulates germ cell differentiation and retinoic acid signaling in mouse ES cells. Reprod Toxicol. 2012 Nov;34(3):463-70. doi: 10.1016/j.reprotox.2012.06.001

Baltus AE, Menke DB, Hu YC, Goodheart ML, Carpenter AE, de Rooij DG, Page DC. In germ cells of mouse embryonic ovaries, the decision to enter meiosis precedes premeiotic DNA replication. Nat Genet. 2006 Dec;38(12):1430-4. doi: 10.1038/ng1919

Blystone CR, Kissling GE, Bishop JB, Chapin RE, Wolfe GW, Foster PM. Determination of the di-(2-ethylhexyl) phthalate NOAEL for reproductive development in the rat: importance of the retention of extra animals to adulthood. Toxicol Sci. 2010 Aug;116(2):640-6. doi: 10.1093/toxsci/kfq147

Bowles J, Feng CW, Miles K, Ineson J, Spiller C, Koopman P. ALDH1A1 provides a source of meiosis-inducing retinoic acid in mouse fetal ovaries. Nat Commun. 2016 Feb 19;7:10845. doi: 10.1038/ncomms10845

Bowles J, Knight D, Smith C, Wilhelm D, Richman J, Mamiya S, Yashiro K, Chawengsaksophak K, Wilson MJ, Rossant J, Hamada H, Koopman P. Retinoid signaling determines germ cell fate in mice. Science. 2006 Apr 28;312(5773):596-600. doi: 10.1126/science.1125691

Budani MC, Tiboni GM. Ovotoxicity of cigarette smoke: A systematic review of the literature. Reprod Toxicol. 2017 Sep;72:164-181. doi: 10.1016/j.reprotox.2017.06.184

Chassot AA, Le Rolle M, Jolivet G, Stevant I, Guigonis JM, Da Silva F, Nef S, Pailhoux E, Schedl A, Ghyselinck NB, Chaboissier MC. Retinoic acid synthesis by ALDH1A proteins is dispensable for meiosis initiation in the mouse fetal ovary. Sci Adv. 2020 May 22;6(21):eaaz1261. doi: 10.1126/sciadv.aaz1261

Cheng NC, van de Vrugt HJ, van der Valk MA, Oostra AB, Krimpenfort P, de Vries Y, Joenje H, Berns A, Arwert F. Mice with a targeted disruption of the Fanconi anemia homolog Fanca. Hum Mol Genet. 2000 Jul 22;9(12):1805-11. doi: 10.1093/hmg/9.12.1805

Choi Y, Ballow DJ, Xin Y, Rajkovic A. Lim homeobox gene, lhx8, is essential for mouse oocyte differentiation and survival. Biol Reprod. 2008 Sep;79(3):442-9. doi: 10.1095/biolreprod.108.069393

Dupé V, Matt N, Garnier JM, Chambon P, Mark M, Ghyselinck NB. A newborn lethal defect due to inactivation of retinaldehyde dehydrogenase type 3 is prevented by maternal retinoic acid treatment. Proc Natl Acad Sci U S A. 2003 Nov 25;100(24):14036-41. doi: 10.1073/pnas.2336223100

D'Ignazio L, Michel M, Beyer M, Thompson K, Forabosco A, Schlessinger D, Pelosi E. Lhx8 ablation leads to massive autophagy of mouse oocytes associated with DNA damage. Biol Reprod. 2018 Apr 1;98(4):532-542. doi: 10.1093/biolre/iox184

El-Nemr A, Al-Shawaf T, Sabatini L, Wilson C, Lower AM, Grudzinskas JG. Effect of smoking on ovarian reserve and ovarian stimulation in in-vitro fertilization and embryo transfer. Hum Reprod. 1998 Aug;13(8):2192-8. doi: 10.1093/humrep/13.8.2192

Fan X, Molotkov A, Manabe S, Donmoyer CM, Deltour L, Foglio MH, Cuenca AE, Blaner WS, Lipton SA, Duester G. Targeted disruption of Aldh1a1 (Raldh1) provides evidence for a complex mechanism of retinoic acid synthesis in the developing retina. Mol Cell Biol. 2003 Jul;23(13):4637-48. doi: 10.1128/MCB.23.13.4637-4648.2003

Feng CW, Burnet G, Spiller CM, Cheung FKM, Chawengsaksophak K, Koopman P, Bowles J. Identification of regulatory elements required for *Stra8* expression in fetal ovarian germ cells of the mouse. Development. 2021 Mar 9;148(5):dev194977. doi: 10.1242/dev.194977

Flaws JA, Doerr JK, Sipes IG, Hoyer PB. Destruction of preantral follicles in adult rats by 4-vinyl-1-cyclohexene diepoxide. Reprod Toxicol. 1994 Nov-Dec;8(6):509-14. doi: 10.1016/0890-6238(94)90033-7

Hooser SB, Douds DP, DeMerell DG, Hoyer PB, Sipes IG. Long-term ovarian and gonadotropin changes in mice exposed to 4-vinylcyclohexene. Reprod Toxicol. 1994 Jul-Aug;8(4):315-23. doi: 10.1016/0890-6238(94)90047-7

Hu Y, Yuan DZ, Wu Y, Yu LL, Xu LZ, Yue LM, Liu L, Xu WM, Qiao XY, Zeng RJ, Yang ZL, Yin WY, Ma YX, Nie Y. Bisphenol A Initiates Excessive Premature Activation of Primordial Follicles in Mouse Ovaries via the PTEN Signaling Pathway. Reprod Sci. 2018 Apr;25(4):609-620. doi: 10.1177/1933719117734700

Houghtaling S, Timmers C, Noll M, Finegold MJ, Jones SN, Meyn MS, Grompe M. Epithelial cancer in Fanconi anemia complementation group D2 (Fancd2) knockout mice. Genes Dev. 2003 Aug 15;17(16):2021-35. doi: 10.1101/gad.1103403

Jacobson MH, Mertens AC, Spencer JB, Manatunga AK, Howards PP. Menses resumption after cancer treatment-induced amenorrhea occurs early or not at all. Fertil Steril. 2016 Mar;105(3):765-772.e4. doi: 10.1016/j.fertnstert.2015.11.020

Koubova J, Menke DB, Zhou Q, Capel B, Griswold MD, Page DC. Retinoic acid regulates sex-specific timing of meiotic initiation in mice. Proc Natl Acad Sci U S A. 2006 Feb 21;103(8):2474-9. doi: 10.1073/pnas.0510813103

Kumar S, Sandell LL, Trainor PA, Koentgen F, Duester G. Alcohol and aldehyde dehydrogenases: retinoid metabolic effects in mouse knockout models. Biochim Biophys Acta. 2012 Jan;1821(1):198-205. doi: 10.1016/j.bbalip.2011.04.004

Le Bouffant R, Guerquin MJ, Duquenne C, Frydman N, Coffigny H, Rouiller-Fabre V, Frydman R, Habert R, Livera G. Meiosis initiation in the human ovary requires intrinsic retinoic acid synthesis. Hum Reprod. 2010 Oct;25(10):2579-90. doi: 10.1093/humrep/deq195

Li H, Clagett-Dame M. Vitamin A deficiency blocks the initiation of meiosis of germ cells in the developing rat ovary in vivo. Biol Reprod. 2009 Nov;81(5):996-1001. doi: 10.1095/biolreprod.109.078808

Livera G, Rouiller-Fabre V, Valla J, Habert R. Effects of retinoids on the meiosis in the fetal rat ovary in culture. Mol Cell Endocrinol. 2000 Jul 25;165(1-2):225-31. doi: 10.1016/s0303-7207(00)00271-9

Lohff JC, Christian PJ, Marion SL, Arrandale A, Hoyer PB. Characterization of cyclicity and hormonal profile with impending ovarian failure in a novel chemical-induced mouse model of perimenopause. Comp Med. 2005 Dec;55(6):523-7

Lohff JC, Christian PJ, Marion SL, Hoyer PB. Effect of duration of dosing on onset of ovarian failure in a chemical-induced mouse model of perimenopause. Menopause. 2006 May-Jun;13(3):482-8. doi: 10.1097/01.gme.0000191883.59799.2e

Mayer LP, Devine PJ, Dyer CA, Hoyer PB. The follicle-deplete mouse ovary produces androgen. Biol Reprod. 2004 Jul;71(1):130-8. doi: 10.1095/biolreprod.103.016113

Mayer LP, Pearsall NA, Christian PJ, Devine PJ, Payne CM, McCuskey MK, Marion SL, Sipes IG, Hoyer PB. Long-term effects of ovarian follicular depletion in rats by 4-vinylcyclohexene diepoxide. Reprod Toxicol. 2002 Nov-Dec;16(6):775-81. doi: 10.1016/s0890-6238(02)00048-5

Minkina A, Lindeman RE, Gearhart MD, Chassot AA, Chaboissier MC, Ghyselinck NB, Bardwell VJ, Zarkower D. Retinoic acid signaling is dispensable for somatic development and function in the mammalian ovary. Dev Biol. 2017 Apr 15;424(2):208-220. doi: 10.1016/j.ydbio.2017.02.015

Molotkov A, Duester G. Genetic evidence that retinaldehyde dehydrogenase Raldh1 (Aldh1a1) functions downstream of alcohol dehydrogenase Adh1 in metabolism of retinol to retinoic acid. J Biol Chem. 2003 Sep 19;278(38):36085-90. doi: 10.1074/jbc.M303709200

Mu X, Wen J, Guo M, Wang J, Li G, Wang Z, Wang Y, Teng Z, Cui Y, Xia G. Retinoic acid derived from the fetal ovary initiates meiosis in mouse germ cells. J Cell Physiol. 2013 Mar;228(3):627-39. doi: 10.1002/jcp.24172

Niederreither K, Subbarayan V, Dollé P, Chambon P. Embryonic retinoic acid synthesis is essential for early mouse post-implantation development. Nat Genet. 1999 Apr;21(4):444-8. doi: 10.1038/7788

Oktem O, Oktay K. Quantitative assessment of the impact of chemotherapy on ovarian follicle reserve and stromal function. Cancer. 2007 Nov 15;110(10):2222-9. doi: 10.1002/cncr.23071

Rosario R, Stewart HL, Walshe E, Anderson RA. Reduced retinoic acid synthesis accelerates prophase I and follicle activation. Reproduction. 2020 Sep;160(3):331-341. doi: 10.1530/REP-20-0221

Saadeldin IM, Swelum AA, Elsafadi M, Mahmood A, Yaqoob SH, Alfayez M, Alowaimer AN. Effects of all-trans retinoic acid on the in vitro maturation of camel (Camelus dromedarius) cumulus-oocyte complexes. J Reprod Dev. 2019 Jun 14;65(3):215-221. doi: 10.1262/jrd.2018-073

Sharara FI, Beatse SN, Leonardi MR, Navot D, Scott RT Jr. Cigarette smoking accelerates the development of diminished ovarian reserve as evidenced by the clomiphene citrate challenge test. Fertil Steril. 1994 Aug;62(2):257-62. doi: 10.1016/s0015-0282(16)56875-7

Shimada R, Ishiguro KI. Cell cycle regulation for meiosis in mammalian germ cells. J Reprod Dev. 2023 Jun 6;69(3):139-146. doi: 10.1262/jrd.2023-010

Spiller C, Bowles J. Instructing Mouse Germ Cells to Adopt a Female Fate. Sex Dev. 2022;16(5-6):342-354. doi: 10.1159/000523763

Spiller CM, Bowles J, Koopman P. Regulation of germ cell meiosis in the fetal ovary. Int J Dev Biol. 2012;56(10-12):779-87. doi: 10.1387/ijdb.120142pk

Tahaei LS, Eimani H, Yazdi PE, Ebrahimi B, Fathi R. Effects of retinoic acid on maturation of immature mouse oocytes in the presence and absence of a granulosa cell co-culture system. J Assist Reprod Genet. 2011 Jun;28(6):553-8. doi: 10.1007/s10815-011-9579-8

Tuttle AM, Stämpfli M, Foster WG. Cigarette smoke causes follicle loss in mice ovaries at concentrations representative of human exposure. Hum Reprod. 2009 Jun;24(6):1452-9. doi: 10.1093/humrep/dep023

Vernet N, Condrea D, Mayere C, Féret B, Klopfenstein M, Magnant W, Alunni V, Teletin M, Souali-Crespo S, Nef S, Mark M, Ghyselinck NB. Meiosis occurs normally in the fetal ovary of mice lacking all retinoic acid receptors. Sci Adv. 2020 May 22;6(21):eaaz1139. doi: 10.1126/sciadv.aaz1139

Windham GC, Von Behren J, Waller K, Fenster L. Exposure to environmental and mainstream tobacco smoke and risk of spontaneous abortion. Am J Epidemiol. 1999 Feb 1;149(3):243-7. doi: 10.1093/oxfordjournals.aje.a009798

Wong JC, Alon N, Mckerlie C, Huang JR, Meyn MS, Buchwald M. Targeted disruption of exons 1 to 6 of the Fanconi Anemia group A gene leads to growth retardation, strain-specific microphthalmia, meiotic defects and primordial germ cell hypoplasia. Hum Mol Genet. 2003 Aug 15;12(16):2063-76. doi: 10.1093/hmg/ddg219

Xu Y, Ashley T, Brainerd EE, Bronson RT, Meyn MS, Baltimore D. Targeted disruption of ATM leads to growth retardation, chromosomal fragmentation during meiosis, immune defects, and thymic lymphoma. Genes Dev. 1996 Oct 1;10(19):2411-22. doi: 10.1101/gad.10.19.2411

Younis JS, Iskander R, Fauser BCJM, Izhaki I. Does an association exist between menstrual cycle length within the normal range and ovarian reserve biomarkers during the reproductive years? A systematic review and meta-analysis. Hum Reprod Update. 2020 Nov 1;26(6):904-928. doi: 10.1093/humupd/dmaa013

Yu M, Yu P, Leghari IH, Ge C, Mi Y, Zhang C. RALDH2, the enzyme for retinoic acid synthesis, mediates meiosis initiation in germ cells of the female embryonic chickens. Amino Acids. 2013 Feb;44(2):405-12. doi: 10.1007/s00726-012-1343-6

Zhou Q, Nie R, Li Y, Friel P, Mitchell D, Hess RA, Small C, Griswold MD. Expression of stimulated by retinoic acid gene 8 (Stra8) in spermatogenic cells induced by retinoic acid: an in vivo study in vitamin A-sufficient postnatal murine testes. Biol Reprod. 2008 Jul;79(1):35-42. doi: 10.1095/biolreprod.107.066795
